# Enlightening discriminative network functional modules behind Principal Component Analysis separation in differential-omic science studies

**DOI:** 10.1038/srep43946

**Published:** 2017-03-13

**Authors:** Sara Ciucci, Yan Ge, Claudio Durán, Alessandra Palladini, Víctor Jiménez-Jiménez, Luisa María Martínez-Sánchez, Yuting Wang, Susanne Sales, Andrej Shevchenko, Steven W. Poser, Maik Herbig, Oliver Otto, Andreas Androutsellis-Theotokis, Jochen Guck, Mathias J. Gerl, Carlo Vittorio Cannistraci

**Affiliations:** 1 Biomedical Cybernetics Group, Biotechnology Center (BIOTEC), Center for Molecular and Cellular Bioengineering (CMCB), Department of Physics, Technische Universität Dresden, Tatzberg 47/49, 01307 Dresden, Germany; 2 Lipotype GmbH, Tatzberg 47, 01307 Dresden, Germany; 3 Membrane Biochemistry Group, DZD Paul Langerhans Institute, Technische Universität Dresden, Tatzberg 47/49, 01307 Dresden, Germany; 4 Integrin Signalling Group, Fundación Centro Nacional de Investigaciones Cardiovasculares Carlos III, Melchor Fernández Almagro 3, 28029 Madrid, Spain; 5 MPI of Molecular Cell Biology and Genetics, Pfotenhauerstrstraße 108, 01307 Dresden, Germany; 6 Center for Regenerative Therapies Dresden (CRTD), Center for Molecular and Cellular Bioengineering (CMCB), Technische Universität Dresden, Fetscherstraße 105, 01307 Dresden, Germany; 7 Department of Internal Medicine III, University Hospital Carl Gustav Carus at the Technische Universität Dresden, Fetscherstr.74, 01307 Dresden, Germany; 8 Cellular Machines Group, Biotechnology Center (BIOTEC), Center for Molecular and Cellular Bioengineering (CMCB), Technische Universität Dresden, Tatzberg 47/49, 01307 Dresden, Germany; 9 Department of Stem Cell Biology, Centre for Biomolecular Sciences, Division of Cancer and Stem Cells, School of Medicine, University of Nottingham, Nottingham NG7 2RD, U.K

## Abstract

Omic science is rapidly growing and one of the most employed techniques to explore differential patterns in omic datasets is principal component analysis (PCA). However, a method to enlighten the network of omic features that mostly contribute to the sample separation obtained by PCA is missing. An alternative is to build correlation networks between univariately-selected significant omic features, but this neglects the multivariate unsupervised feature compression responsible for the PCA sample segregation. Biologists and medical researchers often prefer effective methods that offer an immediate interpretation to complicated algorithms that in principle promise an improvement but in practice are difficult to be applied and interpreted. Here we present PC-corr: a simple algorithm that associates to any PCA segregation a discriminative network of features. Such network can be inspected in search of functional modules useful in the definition of combinatorial and multiscale biomarkers from multifaceted omic data in systems and precision biomedicine. We offer proofs of PC-corr efficacy on lipidomic, metagenomic, developmental genomic, population genetic, cancer promoteromic and cancer stem-cell mechanomic data. Finally, PC-corr is a general functional network inference approach that can be easily adopted for big data exploration in computer science and analysis of complex systems in physics.

Omic sciences are contributing to revolutionize current biomedicine towards a precision and patient-tailored approach. Indeed, recent advances in high-throughput technologies have led to a growing amount of omic data in several branches of biomedicine, and consequently boosted the development of a large number of network-inference methods (also called reverse engineering methods), in order to seize the behaviour of the underlying biosystems^1^.

Inferring or ‘reverse-engineering’ biological networks can be defined as the process of probing interactions between molecular components from experimental data through computational analysis^2^. Reverse-engineering algorithms divide into many subtypes, however two of them play an important role in biomedicine^3^. *Physical interaction* approaches aim at predicting biophysical interactions among molecules, for instance structural protein-protein interactions^4^,^5^,^6^,^7^,^8^. *Influence or functional interaction* approaches aim at predicting associations such as *correlation* or *causality* among molecules. A *correlation network* is a widely used representation paradigm of the associations between the parts that compose a complex system. Each edge in this network is undirected and its weight indicates the level of correlation between the trends of two variables that are symbolically represented by two connected nodes. However, correlation does not imply causality and the correlation network does not represent relations of dependency between the variables (nodes) in the network. Such type of information would imply the inference of directionality on the edges leading to a directed graph representation of the system, which is also called *regulatory network*. Correlation networks are a very general representation paradigm, which is a point of strength for computational systems biology applications, in fact they can be extracted from any type of omic data regardless of the hypothesis of variable dependency, yet offering a very important information on the internal functional association and organization of the system parts. Their clear point of weakness is that they cannot describe the internal causality (conditioning or dependency) between the parts of the system. On the other hand, although regulatory networks offer a deeper characterization of the mechanisms from which complexity arises, they are a more specific representation paradigm that cannot be applied to all omic data *tout court*. The inference of regulatory networks is much more complicated - in many cases it requires more samples and higher data quality - to get a good estimation of the variable dependency (edge directionality).

In computational systems biology, the gene expression profiles are used to build gene co-expression networks, which are correlation networks where the nodes correspond to genes, and the edges between nodes represent significant co-expression relationships. Co-expression networks were found extremely useful for analysing microarray and RNA-Sequencing data, since they can identify functional gene modules, i.e. densely connected subnetworks of genes sharing similar patterns of expression^9^. Usually in co-expression networks all the genes present in a dataset are used to construct the network. However, for computational reasons, the network can be restricted to the most varying genes^10^, and genes with low variance can be filtered out. In the same way, a correlation network can be constructed from any omic dataset by using the most varying features. Nonetheless, it will not point out what features are more important for discriminating two or multiple conditions, for instance control versus disease samples in case-control studies. To this aim, a further development of this approach consists in using a univariate statistical test (for instance Mann-Whitney test) singularly for each omic feature, followed by multiple testing correction (for instance Benjamini*-*Hochberg adjustment). This procedure selects the features that are significantly different between two or more conditions in a *univariate* way (considering each feature one at a time), and then constructs the correlation network between the significant omic features that are discriminative. In practice, a *linear univariate-discriminative* correlation network is obtained. For simplicity, we will call the outcome of this method: P-value (correlation) network.

As a matter of fact, here we will focus our attention on universal methods for inference of correlation networks in omic data in general, whereas the development of methods for inference of regulatory networks is out of the scope of this study. For this interesting and different subject we refer to the DREAM project^11^, where the performance of regulatory network reverse-engineering was shown to vary both across species, and within the same category of inference methods. A plethora of methods are available for gene network reverse-engineering, but few methods were developed and extensively tested for revealing discriminative associations in omic systems in general. Among them, the P-value correlation network is the most employed for the analysis of omic data, because of its simple and fast application and the straightforward interpretation of the results. A first aim of this study is to offer a valid (but still easy to adopt and interpret) *multivariate* alternative to the P-value networks. Therefore, we will present a method for *linear multivariate-discriminative* correlation network reverse engineering that, thanks to its multivariate nature can help to stress and squeeze out the underlying combinatorial and multifactorial mechanisms that generate the differences between the studied conditions. Notably, the P-value network approach is hypothesis-driven, however our goal is to exploit a data-driven and unsupervised approach, because this can also reveal interesting but unknown sample patterns and can efficiently deal with a small sample size. Between the data-driven methods, the Principal Component Analysis (PCA) is without doubt one of the most used unsupervised linear multivariate algorithms for data exploration and visualization in omic science^12^,^13^. It reduces the data dimensionality while retaining most of the variation in the data, and can be used to detect hidden/unknown patterns of sample discrimination, that emerge according to new inferred variables (the principal components) that are linear combinations of the original variables^14^. Unfortunately, to the best of our knowledge, no algorithm available in literature is able to provide a network representation of the internal relations between the discriminative features that are associated to a PCA visualization in which a sample separation emerges. This could be extremely useful to juxtapose to a PCA plot also a diagram that accounts for the feature associations that contribute to generate the visualized sample segregation. Therefore, here, for the first time, we want to propose a method - which we named *PC-corr* – that uses the PCA loadings to perform unsupervised inference of a linear multivariate-discriminative correlation network.

To summarize, the goal to introduce the use of PC-corr networks is not to infer causality, but to offer a *multivariate alternative* to the P-value networks in order to explore the functional associations of the omic features at the systems’ level. The collection of the inferred associations between selected features creates a network where discriminative modules can be investigated by imposing different cut-offs to the interaction values. This process allows to explore the network structure and organization at different levels. Hence, the PC-corr networks can be – like P-value networks – thresholded at different levels in order to enlighten discriminative network modules of omic features that can be useful in gaining biomedical understanding and insight in support of the sample discrimination obtained using PCA. In practice, the PC-corr network can be a valid tool to add in many studies that use PCA to unveil the relational organization of the features that participate to the sample segregation in the projected space.

## Results

### The reasons to build a data-driven method based on unsupervised learning and PCA

For a general omic dataset, we developed a data-driven method to construct discriminative correlation networks relying on a prior unsupervised analysis and sample projection in a visualization space by PCA. The advantages to design our proposed data-driven approach based on an unsupervised (the labels are not used for learning the data projection) and parameter-free (the algorithm does not require tuning of any parameter for learning the projection) method such as PCA are many. First, regardless of the label availability or reliability, this can help to reveal the presence of hidden/unknown sample patterns in the data, with the additional benefit that the selected features are not biased by any supervised hypothesis, and, instead, they emerge naturally from the hidden multidimensional structure of the data. Second, unlike any supervised feature selection method, PCA also does not overfit the data^11^, a clear advantage for small sample-size dataset analysis – very frequent in biomedicine - or pilot studies in which knowledge uncertainty represents a significant gap. Third, conceptually it might not be clear how an unsupervised method could be better suited to discriminate between conditions compared to a suitable supervised approach, however a vast amount of recent literature proves and discusses this evidence. Smialowski *et al*.^12^ offered a solid in-silico proof of how PCA-loading-based feature selection can perform well preventing overfitting in contrast to supervised strategies. Cannistraci *et al*. proved how unsupervised techniques outperform support vector machine on proteomic and genomic datasets^14^. In a second study, Cannistraci *et al*. showed how unsupervised techniques could largely outperform supervised techniques for network-based prediction of protein interactions^5^. Zagar *et al*. in a very extensive study on stage prediction of embryonic stem cell differentiation from genome-wide expression data demonstrated on even 10 different datasets the supremacy of unsupervised on supervised techniques^15^. In particular and surprisingly, an unsupervised method such as PCA could outperform supervised methods such as partial least squares and support vector machine. The reason behind this apparent paradox finds an explanation in one outlandish expression coined by Richard E. Bellman in dynamic programming^16^: *curse of dimensionality*. Omics datasets are high-dimensional and present a larger number of features (variables) than samples. The fact that the multidimensional volume (in which the samples lie) grows very fast as a function of the number of features is called curse of dimensionality and represents an important problem in big data analysis because - roughly speaking - it means that the more features I have, the more samples I need for a ‘good’ analysis. To be more technical, the volume of the hyperspace increases so fast, as a function of the number of dimensions/features/variables, that the available data become sparse. This sparsity is problematic, in particular for supervised methods that strongly suffer of overfitting in a sparsity scenario, because the amount of data they need to support the prediction often grows exponentially with the dimensionality. In fact, model and parameter estimation becomes very difficult when the number of samples are much lower than the number of features, and this worsen because the data should be divided in a training test and validation set. In practice, the supervised learning is applied even on a smaller cohort of samples that constitute the training set. The curse of dimensionality effectively prevents supervised learning to be more efficient than unsupervised learning on many omics datasets where the sample to feature unbalance is often significant. On the contrary, it is empirically proven that PCA works fine also for big omic datasets (like the one we mentioned few lines above) even when the sample to feature ratio is larger than 1:1000, for instance Ringner^13^ provided a demonstration of this effectiveness on a dataset of 27,648 genes expressions in 105 breast tumour samples. Finally, as a matter of fact, at the basis of the current machine learning revolution initiated by means of deep learning there is unsupervised feature learning, as well as the advantages offered by unsupervised pre-training^17^.

### Overview of PC-corr network construction and its evaluation

PC-corr is a method for unsupervised and parameter-free inference of a linear multivariate-discriminative correlation network. The method is unsupervised, parameter-free, linear and multivariate, because it is based on the loadings of PCA, which is a linear multivariate unsupervised and parameter-free transformation. Furthermore, starting from the PCA loadings there are not additional requirements of parameter tuning to generate the PC-corr networks. In brief, the PC-corr network is a weighted graph, whose values are obtained by combining the loadings of the nodes that are adjacent to an edge with the Pearson correlation of the same nodes. The PC-corr value of an edge is high if and only if both the nodes’ loadings and the nodes’ Pearson correlation are simultaneously high, and this is achieved using a minimum operator (for details refer to the Methods section). It is important to mention that, in general, we can associate a PC-corr network to any principal component (PC), because the PC-corr network is based on the loadings of the selected PC. In particular, our algorithm is based on the fact that the PCA suggests, along one of its PCs, a sample discrimination that can be unknown (a new pattern is discovered) or known (the presence of a known pattern, for which the labels are given, is confirmed). In the absence of labels, the presence of a pattern based on a discrimination along one of the PCs can be detected directly by visual inspection (not automated procedure) or using clustering algorithms (automated procedure). This generates a hypothesis on the presence of a possible unknown sample separation (label inference) but it requires to be validated with external information. In this case, the method to project the data remains unsupervised and therefore also the PC-corr network associated to the discriminative PC is still the result of an unsupervised transformation. The only, but important, problem that remains open is to make sense of the inferred ‘potential’ sample labels. As a common practice, many researchers visually inspect the patterns that unsupervisedly emerge in the PC1-PC2 projection of PCA, and they exploit the presence of unknown sample segregations in order to generate new hypothesis on the origin of their patient differentiation^14^. The new labels, which coincide with the discovered sample segregation, are compared with the information available in biomedical or clinical databases on the patient’s conditions or, very often, are employed to design novel experiments to verify or validate the new hypotheses and intuitions that the sample pattern suggested to the researcher. A clear example of this process is provided in the section below, dedicated to the lipidomic dataset. On the other hand, in the presence of known labels, PCA is very useful to reduce overfitting in the feature selection and to have an unbiased confirmation of the presence of the sample discrimination^12^. Finally, to verify that the pattern discrimination along the selected PC is statistically significant, we suggest adopting a statistical test according to the inferred or the known labels. In the absence of any sample discrimination - suggested via PCA projection and confirmed by a statistical test - our method cannot be used. In addition, if the sample discrimination along a PC is statistically significant for inferred labels that do not match with any known meaningful biomedical or clinical information, the related PC-corr represents a last resort to speculate about the origin of the sample segregation and to generate hypothesis for new validation experiments.

The presence of a module in a network representation appears as a highly interconnected group of nodes, and in case of many modules hierarchically organized, the definition of modules is reserved for potentially overlapping dense subgraphs. The PC-corr network can be used to enlighten the presence of discriminative functional modules by increasing a threshold to remove weak links (connectivity with values lower than the threshold) from the network. Using this process, modules of nodes that are characterized by a strong discriminative correlation emerge in the PC-corr network. These modules are discriminative because they originate from the loadings associated to the discriminative PC, and since the nodes involved in each module have a common correlation, they potentially share a similar function.

**Figure 1:**
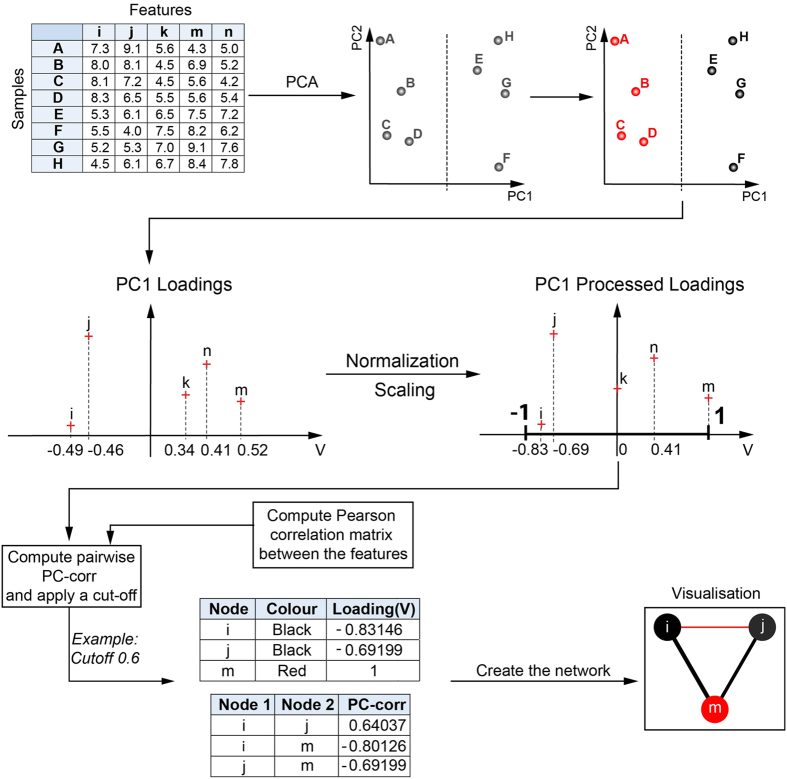
PC-corr network. The omic dataset is analysed unsupervisedly by PCA, which discriminates the samples in two groups (red and black) along PC1. The discriminative correlation network is constructed thereof. Indeed the features’ loadings of the most discriminative dimension (PC1 in the example) are normalized with the heuristic function and scaled, then they are combined together with the Pearson correlations on the features, for the calculation of the edge values 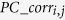. Finally, after applying a cut-off on the edge weights 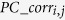, two tables (Edge weight/colour table and Node weight/colour table) are obtained in order to visualize the related network by a network visualizer such as Cytoscape.

In the explanatory Fig. 1, we give a general idea of the PC-corr method without entering in the algorithm details that, instead, are given in Supplementary Table S1. Figure 1 reports the case study where PCA is performed on the omic dataset and unsupervisedly separates the samples in two groups along PC1. We normally compute centered and non-centered PCA and we choose the one that offered better visualization result in sample discrimination. In case of similar performance, we opted for the centered PCA that is the most common employed in omic studies. There is no universal rule for when centering transformation should be used^18^,^19^. Nevertheless, non-centering has been shown to offer several advantages^18^,^19^. This is particularly evident in visualization tasks, when the set of points that form each cluster is distributed around the centre of the mass in the high-dimensional space^5^. Using the embedding in two dimensions after the centering transformation, the points tend to overlap around the origin of the first two dimensions. However, in most cases, executing the embedding without centering can reduce this crowding issue. As we said, the selection of the centering procedure is evidence based.

After that the presence of two groups is detected, they are attributable to two diverse (potential or already known) sample states or experimental conditions, which are accordingly visualized adopting two diverse colours, for instance red or black dots. Here, the separation in two groups is employed in order to provide a simple example, but more in general the unsupervised analysis could also provide a multiple group separation. Then the PC1-loadings - which represent the weights (importance) that each feature has in contributing to the sample separation on PC1 - are combined together with the features’ Pearson correlations (see Supplementary Table S1 for details on the mathematical formula) in order to generate the PC-corr edge weights. Hence, each pairwise feature interaction assumes a PC-corr weight: the higher is this value (in a scale between 0 and 1), the more the two features coparticipate in discriminating the two groups along PC1. The collection of the PC-corr weights, characterizing all pairwise feature interactions, is in practice a PC-corr weighted network having the features as nodes. We invite the reader to refer to Fig. 1 for a general overview of the method.

Summarizing for a general case: this easy, fast and novel method infers the discriminative linear associations existing between features, by combining the result of a preliminary unsupervised analysis (PCA), with the pairwise Pearson correlations between the features, to ultimately build and explore the discriminative network functional modules. The resulting network, that we called PC-corr network, pinpoints the *discriminative correlations*: correlations between those features that explain the discrimination of the samples along a principal component PCn. This is achieved by combining the PCn loadings (eigenvectors) and the pairwise Pearson correlation (for more details, see Methods).

Moreover, the structure of the interaction network is probed by applying different cut-offs, which allow to progressively reveal different discriminative network modules, that otherwise appear connected and continuous. There is not yet an automatic cut-off selection procedure, but different options are currently under investigation as a follow-up study. Notably, the free selection of the cut-off is the current most employed strategy in P-value network inspection. Hence, we suggest also in PC-corr networks that the operator tunes the cut-off in relation to his/her needs to explore the network structure and its organization at different levels. Nevertheless, a clear strategy to select one or multiple cut-offs in case of PC-corr networks is proposed in the next section entitled: *How to choose the cut-off in PC-corr networks*.

We compare our approach to the P-value network, i.e. a variation of the co-expression network that selects and represents only the Pearson correlations between significantly different features (as we already discussed in the Introduction). Additionally, we tested PC-corr network robustness by leave-one-out cross validation (LOOCV; the procedure is explained in the Methods section and results are shown in Supplementary information).

We decided to show the effectiveness of PC-corr networks on six datasets from different omic fields, ranging from lipidomics, to metagenomics, developmental genomics, population genetics, carcinoma cell-wide promoteromics and cancer stem cell mechanomics. As a final remark, in the first analysed dataset (coming from lipidomics) we compared PC-corr network with P-value mutual information (MI) network. Since MI is a measure of nonlinear association between two features, this analysis was oriented to provide a further comparison also versus nonlinear association networks.

### How to choose the cut-off in PC-corr networks

In this section, we discuss a ‘rule of thumb’ for selecting a cut-off. The optimal cutoff is indeed quite dataset dependent, but we suggest the general cut-off range to be from 0.5 to 1. The higher the cutoff, the more discriminative the features are and the higher (stronger) the correlation between them will be. We suggest the exploration of the network structure and organization from cut-off 0.5 since this requires the features’ Pearson correlation higher than 0.5, while below this threshold the features are considered weakly correlated. However, this does not indicate that applying a cut-off lower than 0.5 will not get meaningful outcomes, but it has to be kept in mind that there could be many relatively low discriminative features and weakly interactions could be found and represented in the network. In addition, we should clarify the concept of *frustration*. In the PC-corr network, a red edge links nodes that display the same behaviour (positive correlation) and have therefore the same colour, while a black edge connects nodes that are negatively correlated, hence nodes with antithetical colours. Frustration occurs when some edges in the network do not follow this pattern, i.e. they represent positive correlations between nodes with opposite behaviour (opposite colours) or negative correlations between nodes with identical behaviour (same colours).

Usually, in our analyses on six datasets from different omic sciences, we selected cut-off greater or equal than 0.5, and no frustration appeared. However if any frustration occurs, we suggest two possible solutions:

(a) discard the links if the percentage of frustration is less that <5%, i.e. the edges under frustration represent less than 5% of the total number of edges in the represented PC-corr network;

(b) increase the cut-off till no more frustration is present, i.e. the percentage of frustration is 0%.

In the next studies, we will adopt the degree of freedom offered by the cut-off to perform a percolation-based investigation of the network, the detection of weak links and the automatic inference of the hierarchical organization between the discriminative network modules.

### Lipidomics

**Figure 2:**
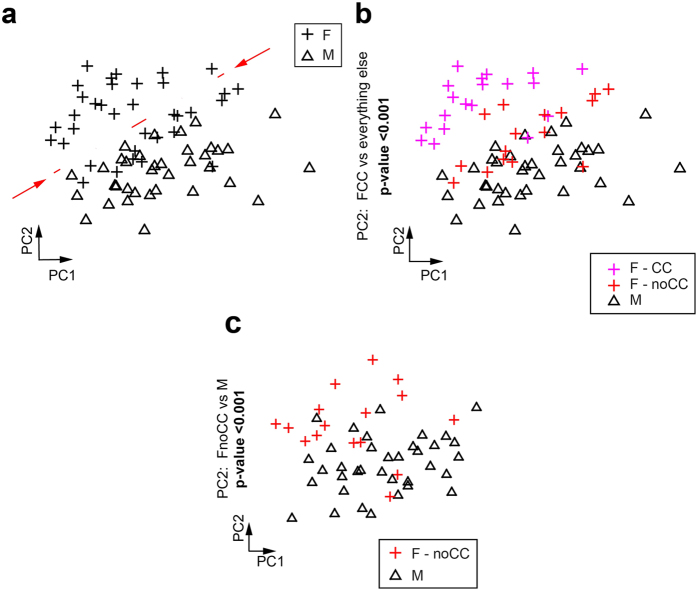
Lipidomics dataset: PCA results. (**a**) PCA discovered the presence of a well-defined dichotomy indicating a subpopulation inside the female cohort (black crosses). (**b**) PCA separates the females using contraceptive medication from the contraceptive-free females: females – contraceptive (F-CC, red crosses), females – no contraceptive (F-noCC, magenta crosses) and males (black triangles). In addition the separation between the two groups (FCC vs everything else) was significant. (**c**) Significant separation was proven along PC2 between the F-noCC and M.

Lipidomics is a rapidly growing omic field^20^. Absolute quantification of lipid abundance by shotgun mass spectrometry is generating high-throughput datasets^20^, which contain unveiled lipid signatures in a plethora of conditions and organisms. In particular, high-throughput human blood plasma lipidomics represents a new important tool for precision medicine, which can help to understand the causes and predict effective combinatorial biomarkers in metabolic disorders and beyond^21^. However, discovering and investigating the peculiarities of a healthy plasma lipidome is still an open task. Here we considered a lipidomic dataset composed by the systematic quantification of 281 lipid species from 27 major lipid classes in the human plasma samples of 71 healthy young Caucasians (36 males and 35 females)^22^. Since we did not have any verified a-priori information, but only a gender-based hypothesis on the possible segregation of the samples, we performed an unsupervised analysis of the whole dataset by PCA. This would allow finding hidden patterns that might visually emerge in the representation space composed by the first two-dimensions of embedding. Surprisingly, the unsupervised analysis revealed a dichotomy inside the female cohort, which was split in two distinct sub-populations (Fig. 2a). Since plasma lipids are strongly related to sex hormones, we postulated that the evident separation might be connected to the use of contraceptive drugs. Therefore, we asked to recover clinical information on the use of contraceptives by the female patients. This information proved that the significant separation on PC2 was contraceptive-driven (Fig. 2b). In fact, hormonal contraceptives may affect the overall change in lipid levels. However, in our analysis we focused on revealing the gender difference from the lipidomic point of view, excluding any bias related with the drug intake. Hence, we confronted the male cohort and the subpopulation of contraceptive-free females (denoted by FnoCC).

**Figure 3:**
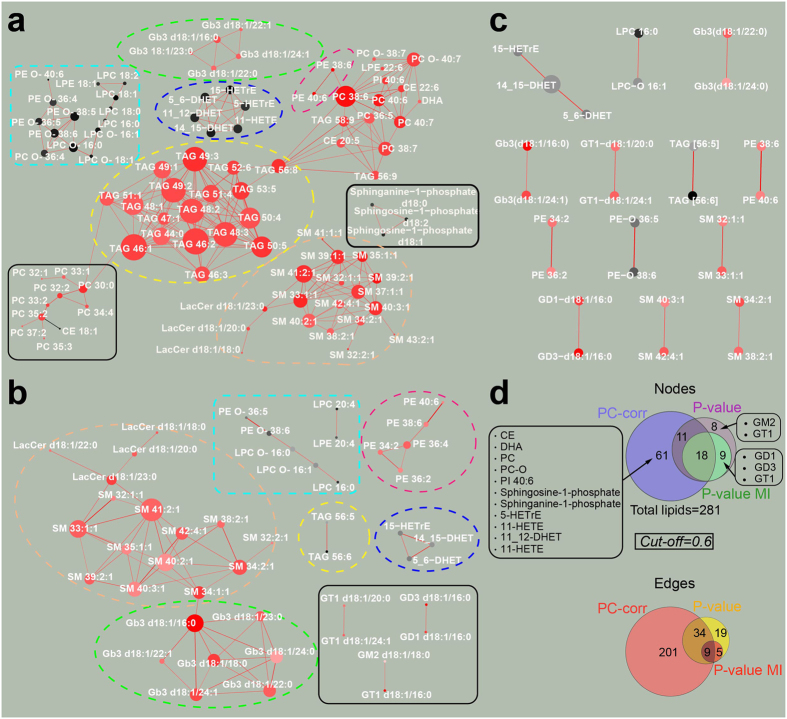
Lipidomics dataset: networks. (**a**) The PC-corr network was constructed according to the loadings of PC2 (Fig. 2c), since PCA could significantly (p < 0.001) separate F-noCC from M, therefore reflects discriminative network modules related to real (absence of contraceptive effect) gender difference. Red nodes indicate higher lipid abundance in F-noCC, while black nodes indicate higher lipid abundance in M. (**b**) The P-value network is a correlation network (Pearson-correlation cut-off = 0.6) of the lipids that are significantly different (Mann-Whitney test, Benjamin-adjusted p-value < 0.05) between F-noCC and M. Again, the node colour has the same meaning of plot in (**a**). Modules circled with a dashed line are present in both networks (**a,b**), while modules circled with a black solid line are present exclusively in one of the two networks. (**c**) The P-value MI network is a mutual information network (mutual-information cut-off = 0.6) of the lipids that are significantly different (Mann-Whitney test, Benjamin-adjusted p-value < 0.05) between F-noCC and M, similarly to the P-value network. Again, the node colour has the same meaning of plot in (**a,b**). In the P-value MI network, all the edges between lipids were already inferred by the P-value network, that contains even more lipid interactions. (**d**) For F-noCC vs M, the PC-corr network - constructed according to the PC2 significant discrimination of (Fig. 2c) - and the P-value and P-value MI networks (cut-off = 0.6) show differences in the numbers of nodes and edges, as illustrated in the proportional Venn diagrams. Some lipids are present only in the PC-corr network (as shown in the box on the left), while much less are present exclusively in the P-value network and, for some of them, also in the P-value MI network (as shown in the boxes on the right).

In this case also, significant separation was proven along the PC2 (Fig. 2c). This discrimination is explained by the network constructed with our approach (Fig. 3a, cut-off = 0.6), where the red lipids are more abundant (higher) in the FnoCC cohort while the black ones are higher in the male group. The PC-corr network could distinctly reveal discriminative lipid modules and interconnected sub-modules related to the gender separation. Lipids, which are highly connected in modules, belong mainly to the same lipid class. This is expected as these lipids share biosynthetic pathways and regulatory mechanisms. More interesting are the connections revealed by the network, which involve lipids of unrelated classes. While some of these connections can be explained (LPC O- 16:0 may be the lyso-product of PC O- 36:4), others are less obvious and may provide hints for future research directions.

Nonetheless, comparing (at the same cut-off level) the original PC-corr network (Fig. 3a) to the P-value network (which is the correlation network of the lipids that most significantly differentiate between the two groups, Fig. 3b) a noticeable difference emerges in the number of nodes and edges (Fig. 3d). In fact, the majority of the lipids in the PC-corr network are absent in the P-value network, while few lipids present in the P-value network are missing in the PC-corr network. This can also be observed in the modules, which appear ‘depleted’ in the P-value network in comparison to the PC-corr one. For instance, there is a clear difference in the common highlighted modules (circled in both networks with a dashed coloured line), as well as there are modules (circled in the network with a filled black line) which are present only in one of the networks. Substantial differences between the two networks consist in the presence of few triacylglyceride (TAG), the total absence of the Cholesteryl esters (CE lipids), the Sphinganine- Sphingosine and the Phosphatidylcholines modules in the P-value network (Fig. 3b). On the other hand, the PC-corr network lacks the presence of few isolated lipids such as the GD1, GD3, GM2, GT1. Even comparing (at the same 0.6 cut-off) the PC-corr network to the P-value MI network (i.e. the mutual information (MI) network of the lipids that most significantly differentiate between the two groups, similarly to the P-value network, Fig. 3c) (for details see the Methods section: *Construction of the P-value MI network*), no additional differences (to the ones described before) emerge, since all the interactions in the P-value MI network are contained in the P-value network (Fig. 3d).

All in all, our approach shows more diversity and completeness in the lipid module composition, and reveals the most discriminative functional lipid modules. While the P-value MI network contains single/double interactions and the P-Value network shows rather isolated modules, the PC-corr network also contains multiple connections between these modules. These connections are interesting points of complex metabolite interactivity which should be further investigated. It is a promising sign for a functional discriminative network to also include a major signalling sphingolipid (Sphingosine-1-phosphate).

Furthermore, we represented the possible/putative relation between the lipid chemical structural information and the connectivity of the nodes in the PC-corr network. In Supplementary Fig. S1, node colour diversity reflects the different lipid classes. This shows that the inferred discriminative modules reflect the known correlation between lipids of the same lipid class. However, also less obvious connections, as the degree of fatty acid unsaturation (caused by fatty acid desaturases), are conserved in the network. In Supplementary Fig. S2, node colours reflect the number of double bonds in each lipid species and show aggregations of similarly unsaturated lipids.

In a similar way, in Supplementary Fig. S3 the node colour gradient reflects the number of carbon atoms in the fatty acid chains and it appears that each module is internally characterized by a homogeneous colour gradient, hence by a certain similarity in the number of carbon atoms.

Since for this dataset the approach based on mutual information held a highly broken network with no interesting results, in the next datasets we continued to discuss merely the differences between the PC-corr and P-value networks.

### Metagenomics

Dyspepsia is a common gastric problem mainly characterized by upper abdominal pain or discomfort and heartburn^23^. The primary medicaments for the management of dyspeptic symptoms, which usually involves their long-term use, are over-the-counter antacid drugs known as Proton Pump Inhibitors (PPIs), that inhibit acid secretion in the stomach’s parietal cells, resulting in a higher intragastric pH. Although PPIs are widely prescribed, they are related to several adverse situations, in particular the development of corpus predominant atrophic gastritis in *Helicobacter pylori* positive patients, that is a precursor of gastric cancer, and a higher risk of enteric infections^24^,^25^. These last two conditions are related to the alteration of the gastrointestinal microbiota, therefore this issue has been progressively addressed from a metagenomic perspective^26^,^27^. The first question of clinical relevance is whether PPIs exert any effect on the gastric bacterial communities, and this was investigated in a study by Williams and collaborators that was still solely based on culturable gastric bacteria^28^. More recently, next-generation-sequencing-based metagenomic studies further confirmed that PPIs modify the gastroenteric human microbiota^29^,^30^,^31^,^32^,^33^.

Here, we re-analyzed a dataset by Amir and collaborators^29^, in order to verify whether the PC-corr method could enhance, in comparison to Amir *et al*.^29^, our understanding of the PPI-induced microbial perturbation. In particular, we were interested in promoting a systems biology perspective, pointing out the possible presence of discriminative network microbial alterations. Therefore, we considered the gastric fluid dataset, which comprises eight patients who were sampled at two different time points, that is before and after eight weeks of PPI treatment, for a total of 16 samples. We selected this specific dataset because it clearly shows that PPI treatment alters the gastric fluid microbial communities. Metagenomes were obtained by pyrosequencing fragments of the 16S r-RNA gene on the GS FLX system (Roche). Pyrosequencing has been widely used for genomic and metagenomic sequencing, because it is one of the first next generation sequencing platforms developed for the production of high throughput data and it can produce long reads (up to 800 bp).

PCA analysis of the dataset revealed that samples are separated in two groups according to treatment and their difference is significant (Mann-Whitney test p-value, Supplementary Fig. S4). Since non-centered PCA (ncPCA) offered a better discrimination than centered PCA (cPCA), we built the PC-corr networks using the ncPCA loadings of the second dimension (PC2). Nevertheless, it was not possible to build the P-value network, since the pairwise comparisons for each bacterium yielded no significant results after the Benjamini-Hochberg correction.

This finding represents an emblematic example of the need to complement and integrate the outcomes of P-value correlation networks by introducing the PC-corr method. As a matter of fact, in this very case the P-value network approach delivered no results, although there is clearly a difference between the two conditions being tested.

**Figure 4:**
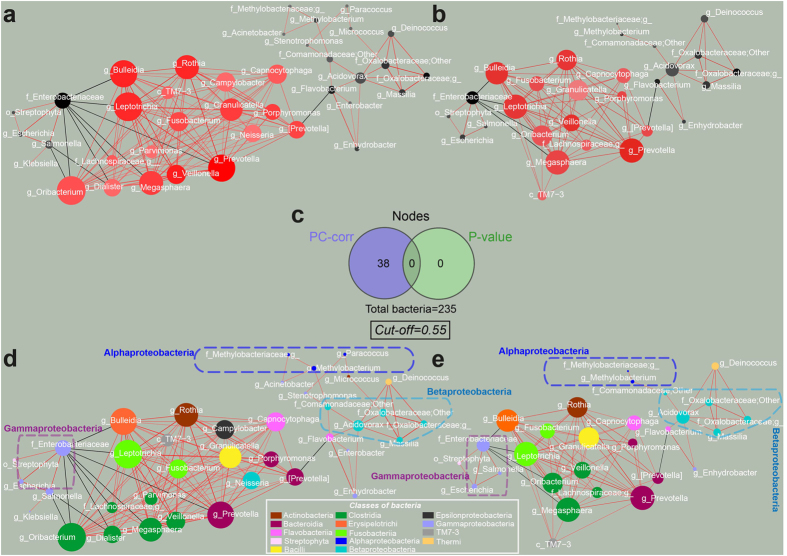
PC-corr networks at two different cut-offs. (**a**) The PC-corr network was constructed at cut-off 0.55 from PC2 loadings since the discrimination of samples before and after PPI treatment was significant along PC2 of PCA. (**b**) As in (**a**), the PC-corr network was also constructed at cut-off 0.6 from PC2 loadings. (**c**) The PC-corr network can find interactions between the bacteria while the P-value cannot, as illustrated in the Venn diagram for the number of nodes. (**d**) PC-corr network in (**a**) coloured according to class-level taxonomy. The coloured dashed lines highlight the classes of bacteria that constitute the black submodules. (**e**) PC-corr network in (**b**) coloured according to class-level taxonomy. The coloured dashed lines highlight the classes of bacteria that constitute the black submodules.

Figure 4 shows the networks obtained by considering two different cut-offs, that is 0.55 and 0.6. Both networks are represented with black and red nodes in the upper part of the figure (Fig. 4a,b), while at the bottom each network appears with its nodes colored according to class level (Fig. 4d,e). Red nodes are bacteria that are more abundant after treatment, while black nodes are bacteria that are more abundant in the samples collected before the treatment.

The bacteria with higher abundance in the untreated samples (black nodes) are split in two submodules which correlate negatively with the group of red nodes (bacteria that display higher abundance in treated samples). A black submodule is made of Gammaproteobacteria (lilac dashed line), represented by Enterobacteriaceae, while the other black submodule contains a group of Betaproteobacteria (light blue dashed line) with members from the Comamonadaceae (Massilia) and Oxalobacteraceae (Acidovorax) families, and a group of Alphaproteobacteria (violet dashed line), and also Betaproteobacteria.

Although clinical metagenomic studies usually consider the entire phylum of Proteobacteria and its overall abundance instead of exploring their class-level behavior, environmental studies generally analyze how the different classes of Proteobacteria change according to the type of habitat or environmental perturbation. In a study on acid mine drainage microbial diversity^34^ the authors found that Betaproteobacteria dominated in moderate pH conditions, while Alphaproteobacteria and Gammaproteobacteria had a strong preference for lower pH environments. Class-related behavior should be investigated also in biological systems, as our network suggests there may well be a pH-related trend in the bacterial composition of the sub-modules. Indeed, it is the main purpose of PPI treatment to increase the stomach pH, and the higher pH of treated patients is known to favor the growth of bacteria that usually reside in the mouth and esophagus and are not adapted to survive the normal gastric acidity^26^,^35^.

Interestingly, when comparing ours to Amir’s findings, the families that result to be significantly decreased following PPI treatment (Moraxellaceae, Flavobacteriaceae, Comamonadaceae and Methylobacteriaceae) actually belong to the black sub-module featuring the Gammaproteobacteria, that is to the group of nodes that have higher abundance before treatment. The Erysipelotrichaceae are instead significantly increased after PPI treatment and in the PC-corr network they accordingly appear in the red sub-module, represented by the genus *Bulleidia*. Other important hubs in the red sub-module are represented by the genera *Prevotella, Granulicatella, Oribacterium, Leptotrichia* and *Rothia*, which belong to the gastric and oral microbiota^36^,^37^,^38^,^39^.

Taken together these results confirm that, although univariate analysis (P-value network) cannot spot any microbial signature, the PC-corr discriminative network method is able to explain the clear and significant PPI-related sample discrimination detected by multivariate analysis (PCA).

### Developmental genomics

One of the key questions in developmental biology is to understand all the mechanisms that are responsible for tissue differentiation^40^. To develop a multicellular organism, cells must obviously differentiate during multiple stages to specialize for different functions.

Tissue specificity depends on spatiotemporal patterns of gene expression which in turn are driven by transcriptional regulatory networks^41^, that means interactions in which a given transcription factor (TF) regulates other TFs, or itself 42. Hence, combinatorial interactions among TFs would be critical to understand tissue-specific expression patterns.

To identify TF interaction networks involved in tissue development, we decided to reanalyse the dataset by Ravasi and collaborators^41^, that contains quantitative (normalized) mRNA profiles of 1,983 Human TFs using quantitative Reverse Transcription PCR (qRT-PCR) across 32 tissues (excluding monoblasts).

**Figure 5:**
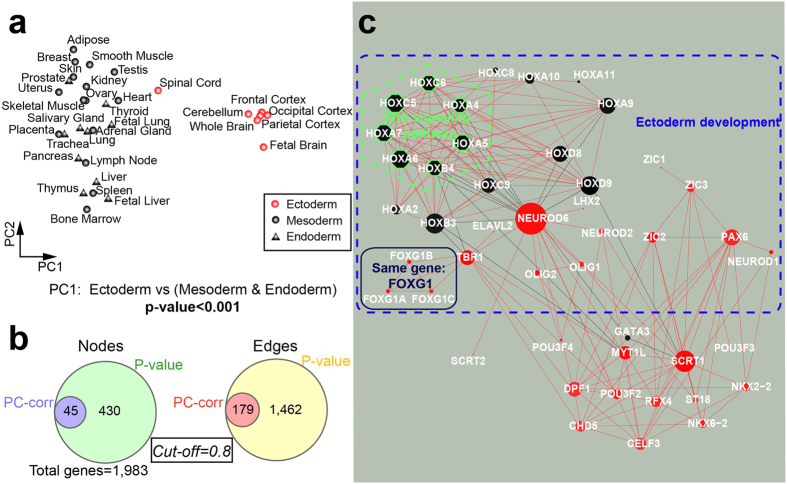
PCA analysis of human tissues according to their embryonic origin and the corresponding TF PC-corr network under cut-off 0.8. (**a**) Linear dimensionality reduction by PCA separates the human tissues derived from ectoderm (red) from the other tissues that arise from endoderm and mesoderm (in black, represented with different symbols) along PC1. (**b**) For cut-off = 0.8, the PC-corr network and the P-value network show differences in the nodes and edges, as illustrated in the proportional Venn diagrams. All the nodes present in the PC-corr network are also contained in the P-value network (figure not shown), as well as the edges. (**c**) Since PC1 is the most discriminative principal component, its loadings were used (after normalization with heuristic function and scaling) to obtain the edge weights of the PC-corr network. Some discriminative modules arose from the network under cut-off 0.8. Colored dashed lined highlight enriched GO biological terms.

Unsupervised analysis of the dataset pointed out two main different groups of tissues that were found consistent with the differences among the tissues at the embryo level (Fig. 5a). In fact, PCA could separate the tissues derived from the ectoderm from the other tissues that arise from the endoderm and mesoderm along PC1, suggesting that expression differences between endoderm and mesoderm are slighter than between these two germ-layers and the ectoderm (for germ layer derivations, see section ‘Germ layer attribution’ in Method). Likewise, PC2 could not differentiate the endoderm and mesoderm-derived tissues and their overlapping is confirmed by the fact that, during development, endoderm patterning is controlled by a series of reciprocal interactions with nearby mesoderm tissues^43^. However, in another study we showed that more advanced machine learning methods for nonlinear dimension reduction (such as minimum curvilinear embedding) could significantly differentiate also mesoderm from endoderm, confirming the presence of a complex and nonlinear relation between these two germ-layers that is difficult to untangle by classical linear transformations^14^.

Then the PC-corr network was constructed from PC1 loadings at 0.8 cut-off level to explain the discrimination of ectodermal tissues from endodermal and mesodermal tissue (Fig. 5c), and to identify co-expressed TF-encoding genes (TFs) in a particular layer. In fact, the red nodes represent TFs that are highly expressed in ectodermal tissues, while the black ones are highly expressed in endodermal and mesodermal tissues.

Using DAVID functional annotation tool we found a significantly enriched pathway (PANTHER pathway P00057, p-value after Benjamini-Hochberg correction <0.01), a Wnt signalling pathway that is crucial for embryonic development in all animal species^44^, and significantly enriched TF-encoding genes that play a specific role in the ectoderm development (PANTHER_BP_ALL, BP00246: ectoderm development, p-value after Benjamini- Hochberg correction <0.01) (Fig. 5c).

Additionally, it is important to notice the role of GATA3, that is highly expressed in mesodermal and endodermal tissues and interacts negatively with SCRT1 and the big hub NEUROD6, which are instead highly expressed in the ectodermal tissues. Previous studies revealed that the encoded protein by SCTR1 may promote neural differentiation (GeneCards database, http://www.genecards.org/), while the encoded protein by NEUROD6 may be involved in the development and differentiation of the nervous system (GeneCards database, http://www.genecards.org/), that is in concordance with the fact that the ectodermal tissues come from different part of the nervous system. Moreover, other middle-size TF-encoded genes share the same trend like PAX6, that is a homeobox gene and key regulatory gene of eye and brain development^45^, and TBR1, a T-box gene involved in the differentiation and migration of neurons and is required for normal brain development^46^,^47^.

Furthermore, the PC-corr network could identify in a triangular module three TF-encoding genes (FOXG1A, FOXG1B, FOXG1C) present in the dataset that actually represent three old names of the same gene, FOXG1^48^.

Comparing it to the P-value network at the same threshold (figure not shown), we note that our approach is able to focus on the main TFs that play an important role for the discrimination shown in Fig. 5a, whereas the P-value network is highly-interconnected (Fig. 5b).

Even if we cross-match the PC-corr network with the P-value network (approximately the same number of nodes), the P-value network (Supplementary Fig. S5) loses its connectivity and starts to break in single interactions, in addition to the absence of the above mentioned NEUROD6, SCRT1 and GATA3.

### Population genetics

Genetic diversity across human populations has been studied widely for many years and the field has progressed rapidly in the last few years^49^,^50^. Complex patterns of genetic variation in populations are the product of demographic and evolutionary events acting at different time scales, including populations migration, expansion, colonization, mutation, genetic drift and selection^50^,^51^.

Understanding human genetic variation is important to discover and describe the genetic contribution to many human diseases and understand the genetic differences within and among populations. In Asia the geographic structure of genetic variation remains enigmatic^52^. For this reason the HUGO Pan*-*Asian SNP consortium conducted the largest survey to date of human genetic diversity among Asians^52^,^53^.

In our analysis, we focused on the Japanese population, represented by 164 individuals who were genotyped at 54,794 autosomal SNPs loci.

**Figure 6:**
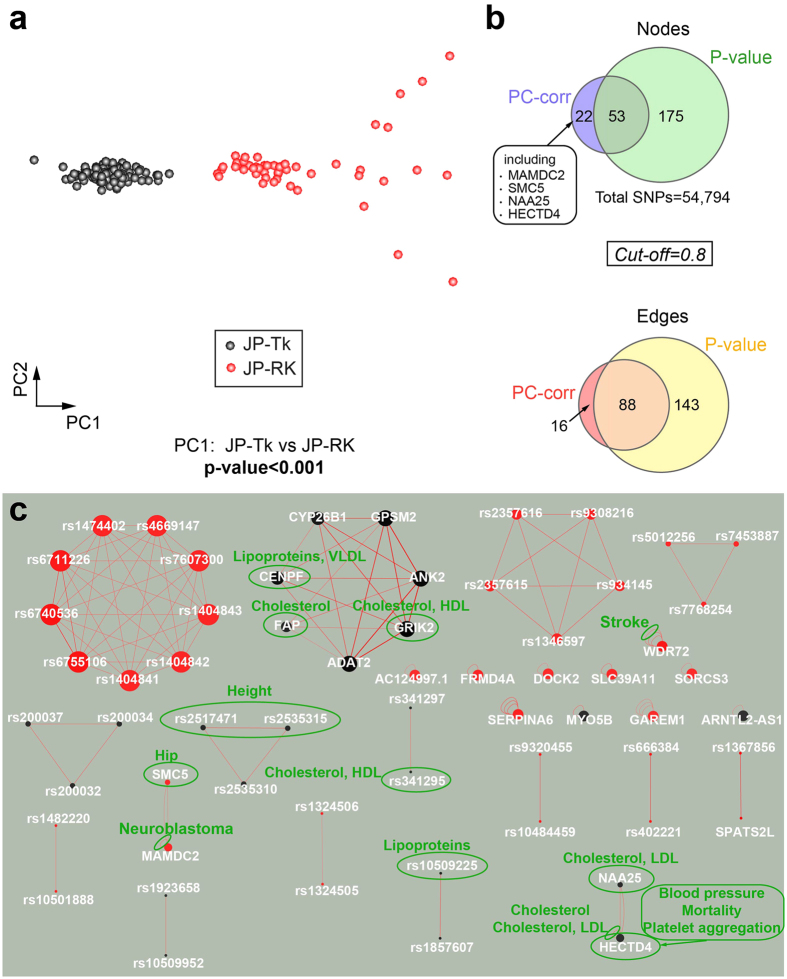
Genetic structure of the Japanese population based on autosomal SNP genotyping. **(a)** PCA discriminated the Japanese population into Tokyotas (JP-Tk) and Okinawans (JP-RK). **(b)** For cut-off = 0.8, the SNP difference between the PC-corr and P-value networks in the nodes and edges is illustrated by the proportional Venn diagrams. Some SNPs are only present in the PC-corr network, like the ones indicated by the genes that contain the mutation (box on the right). Some interactions among the SNPs are also missing in the P-value network. **(c)** As shown in (**a**), PCA detects the difference between JP-Tk and JP-RK along PC1, thus its loadings are employed for the construction of SNP PC-corr network. The SNP name (rs#, in white colour) is changed to the name of the gene that contains the mutation when it is an intron, missense or 3′ UTR variant. Otherwise, it is left unchanged. Some SNPs were significantly associated to phenotypes (green circles and text).

Replacing the missing values with the mode of each SNP^54^ allowed to remove nonlinear perturbations and linearize the data, revealing the separation of the samples into two clear subgroups: Japanese from Tokyo (JP-Tk, including the JPT and JP-ML ethnic groups) and Japanese Ryukyuan from Okinawa (JP-RK) along the first principal component (PC1) (Fig. 6a).

The PC-corr network was constructed at cut-off 0.8 (Fig. 6c), combining the PC1 loadings with the pairwise correlations between SNPs, to find the different patterns of genetic variation in these two Japanese subpopulations.

The red nodes are the SNPs with a higher frequency in the JP-RK, while the black ones have a higher frequency in the JP-Tk. In Fig. 6c the SNPs names are changed to the name of the gene that contains the mutation when it is an intron, missense or 3′ UTR variant. Otherwise, it is left unchanged.

The PC-corr network contains many single/double interactions, a few separated modules, and autocorrelating nodes when the SNPs map to the same gene.

It is important to note that all the modules and single/double interactions not involving SNPs mapped to genes, contain interacting SNPs that lie in the same region between two genes (Supplementary Fig. S6). In particular, the first red module which appears in Fig. 6c is composed of 9 SNPs belonging to the same linkage block. These mutations span over about 2,500 bases in chromosome 2 and exhibit a pairwise disequilibrium value D’ of 1, coupled with Pearson correlation coefficients r^2^ ranging between 0,978 and 1 (calculations were made using the web-based service provided by the Broad Institute SNAP v. 2.2; https://www.broadinstitute.org/mpg/snap/ldsearchpw.php). Disequilibrium and r^2^ values both indicate that these SNPs are in strong linkage disequilibrium, that is to say they co-occur on the same haplotype more often than is expected by chance and is a powerful signal of the population genetic processes^55^. This means there is a non-random association among those SNPs and they are inherited all together.

Moreover, the network identified phenotype-associated SNPs (checked in Ensembl variant database), including disease-related variants (Fig. 6c). For example, the second module on the right contains black nodes (they are related to the JP-Tk group) corresponding to SNPs that are significantly associated to cholesterol levels and lipoproteins. Other black SNPs also mapping to genes (NAA25, ECTD4; bottom right) were significantly associated to the same phenotypes, as well as to mortality and platelet aggregation.

SNPs rs10509225 and rs341295 too are associated to cholesterol, lipoproteins and disease state, while association to stroke and neuroblastoma was shown by two SNPs related respectively to WDR2 and MAMDC2 genes.

Interestingly, some SNPs were instead associated to two phenotype traits, that are height (JP-Tk group), and hip (JP-RK group). In fact, height is a trait that distinguishes the two sub-populations, since the Okinawans are shorter in average than mainland Japanese people^56^. In addition, Okinawans have about 20% fewer hip fractures than mainland Japanese do^57^.

The Japanese began to lose significantly more calcium from their bones than the Okinawans, suggesting the Okinawans preserve their bone density at healthy levels for longer periods of time than the other Japanese, due to protective lifestyle factors (high calcium intake in the food and water, high vitamin D from sun exposure, increased physical activities at older ages, high intake of dietary flavonoids from plant food) (Okinawa Centenarian Study, http://www.okicent.org/study.html).

All in all, the PC-corr network findings confirm that cholesterol levels and lipoproteins affect the JP-Tk population. Indeed, the Okinawan population, characterized by a shorter height than most Japanese, includes the largest proportion of centenarians^58^, who were found to have impressively young, clean arteries, low cholesterol, and low homocysteine levels when compared to Westerners (Okinawa Centenarian Study, http://www.okicent.org/study.html) and less prevalent cardiovascular disease risk factors (such as hypertension, diabetes, hypercholesterolemia and smoking) than most populations^58^,^59^,^60^. These factors help reduce their risk for coronary heart disease by up to 80% and keep stroke levels low (Okinawa Centenarian Study, http://www.okicent.org/study.html). This longest life expectancy was associated to “human longevity genes^61^”, like APOE and FOXO3 genes^58^,^62^,^63^, and lifestyle factors^58^, i.e. reduced energy intake, different composition of diet whose characteristics are low levels of saturated fat, high antioxidant intake, and low glycemic load^64^, level of physical activity and philosophy of life^65^,^66^.

However, we compared our approach to the P-value network at the same high cut-off (figure not shown), and the latter has many more SNPs and interactions between SNPs (Fig. 6b), and does not focus on the significant discriminative SNPs. Also, some SNPs of the PC-corr network are missing (indicated in the box of Fig. 6b by their corresponding accepting genes), like MAMDC2, SMC5, NAA25, HECTD4. Similarly, cross-matching the PC-corr network with the P-value network of approximately the same number of nodes (Supplementary Fig. S7) revealed that the P-value network cannot catch the SNPs that are significantly associated to hip, cholesterol, lipoproteins and disease.

### Carcinoma cell-wide promoteromic dataset

Here, we decided to focus our attention on Carcinoma^67^: an important and frequently occurring type of cancer that arises from epithelial cells originating from the ectoderm or the endoderm^68^. It can be classified into two broadly differentiated classes: small-cell (SCC) and non-small cell carcinoma (NSCC). The latter can be subdivided into many other subtypes, being adenocarcinoma (ADC) and squamous-cell carcinoma (SqCC) the ones with the highest incidence. In particular, SCC is characterized by small undifferentiated cells with a big nucleus and almost no cytoplasm. This type of carcinoma is highly aggressive and, in spite of its initial positive response to chemo and radiotherapy, it is recurrent and poorly prognosed^69^,^70^. SqCC is a type of cancer that begins in the squamous cells, which are a kind of epithelial cells, and can occur in a variety of tissues throughout the body, although it most often develops in sun-exposed areas^71^.

**Figure 7:**
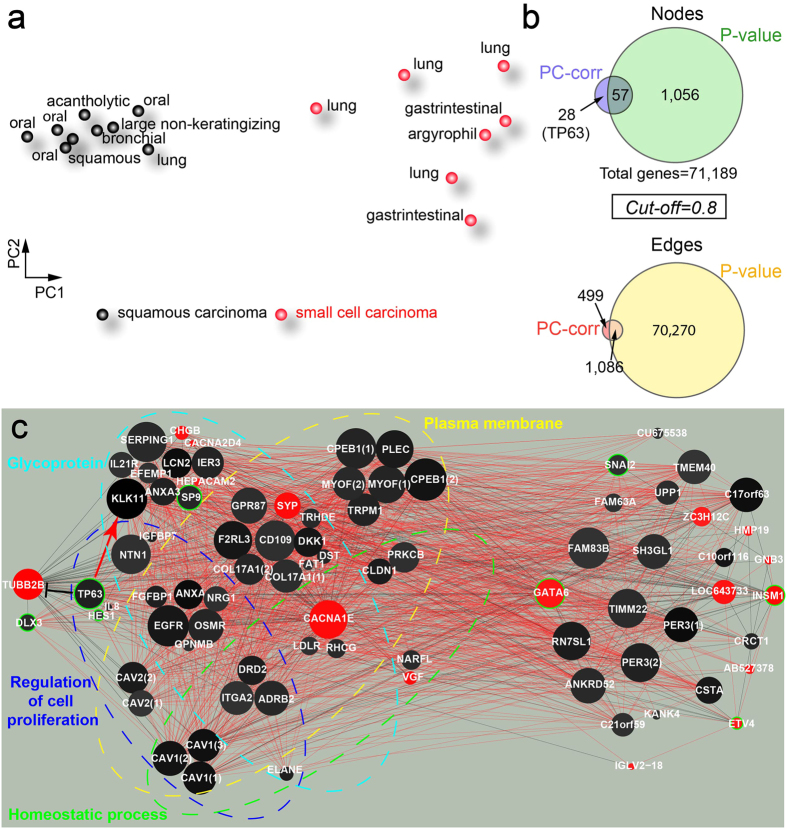
PCA separation of squamous cell carcinoma and small cell carcinoma, and the corresponding discriminative gene network using PC-corr under cut-off 0.8. (**a**) PCA separation of squamous cell carcinoma and small cell carcinoma. (**b**) For cut-off = 0.8, the PC-corr network and the P-value network show differences in the nodes and edges, as illustrated in the proportional Venn diagrams. Some transcripts are only present in the PC-corr network, like TP63. Some interactions too are missing in the P-value network. (**c**) Gene network produced using PC-corr algorithm, where the TF encoding genes have a green border. Black nodes are highly expressed genes in SqCC - black samples in PCA plot, panel (**a**) - and red nodes are highly expressed genes in SCC - red samples in PCA plot, panel (**a**). Genes with a number between brackets denote different CAGE peaks, which potentially indicate different transcripts of that gene. Coloured dashed ellipses highlight enriched GO biological terms.

The dataset to which we had access is a unique source of information because it is based on a particular type of sequencing called Cap analysis of gene expression (CAGE)^72^. CAGE is the method for genome-wide identification of transcription start sites (TSSs) adopted by the Functional Annotation of the Mammalian Genome (FANTOM) consortium, and it is the first technology developed to profile the activity of gene transcription at each promoter site. In particular it concurrently identifies the TSS and from these their promoters, and determines the expression level at each promoter, thus allowing the identification of regulatory elements^73^. The gene expression data we analysed in this work were generated in the FANTOM5 project^74^. In this huge project thousands of mouse and human samples from different cell lines were sequenced using CAGE^72^ with the final aim of understanding transcriptional regulation of gene expression and annotating its promoterome^74^. In our analysis we focused on human small-cell and squamous-cell carcinoma-derived cell line samples (respectively 7 and 9 samples), since these two types of cancers have the poorest prognosis according to latest research^75^,^76^. These samples have 184,827 ‘robust’ CAGE peaks which were obtained by decomposition-based peak identification (DPI)^74^.The peaks without explicit gene annotations were removed and we ended with a total of 71,189 gene features in the dataset. Then, performing dimensional reduction by PCA, we found that SqCC and SCC are clearly separated along the first principal component (PC1) regardless of the tissue the carcinoma lies in (Fig. 7a). This component was used to build the PC-corr network, and find the different patterns of gene expression in these two kinds of carcinoma.

In the network at 0.8 cut-off (Fig. 7c), most of the genes (the black nodes) are highly expressed in SqCC samples, while the remaining genes (the red nodes) are highly expressed in SCC samples, and some of them are transcription factors (TFs, highlighted in green). The EGFR is highly expressed in SqCC, which actually is one of the main targets of SqCC treatment^77^,^78^. We can see that most of the genes that are up-regulated in SCC are markers of neuroendocrine differentiation, for example, VGF^77^,^78^, SYP^79^, CHGB^80^, INSM1^81^,^82^, while HMP19, TUBB2B, CACNA2D4 and GNB3 are highly expressed in neuroendocrine tissues (GeneCards database, http://www.genecards.org/). In contrast, many of the up-regulated genes in SqCC are transmembrane proteins in the plasma membrane, which are responsible for the way the cell interacts with the extracellular matrix, and could play a role in the different migration behavior of both carcinoma subtypes.

With the aid of DAVID^83^,^84^ for functional annotation, we found a group of enriched genes that are annotated as regulating cell proliferation (biological process, GO:0042127, p-value after Benjamini-Hochberg correction <0.05). We also found two more groups of enriched genes, annotated as cell junction (cell component, GO:0030054) and plasma membrane (cell component, GO:0005886), and a protein family characterized as glycoprotein (p-values after Benjamini-Hochberg correction <0.05). These enriched genes were shown to be highly correlated with cancer progression^85^,^86^, together with the genes that regulate cell proliferation. Furthermore, the TF-encoding gene TP63 is highly expressed in SqCC, while the TF-encoding gene INSM1 is highly expressed in SCC (Fig. 7c), which is confirmed by previous studies^82^,^87^. In literature INSM1 is expressed predominantly in small cell lung carcinoma and is highly expressed in tumors of neuroendocrine origin^82^. We further investigated the target genes of each TF, to verify whether they interacted in the network with their corresponding TF. Interestingly, TUBB2B and KLK11 are the target genes of TP63, more specifically, TP63 activates KLK11 and represses TUBB2B^88^. Moreover, TP63 and CSTA play an important role in epithelial tissue maintenance and development, and they are markers of squamous differentiation^89^,^90^,^91^. We know that GATA6 is a TF particular important in mammalian cell differentiation and molecular programs that mediate normal cell differentiation are required for oncogenesis and tumor cell survival in certain cancers^92^. Interestingly, GATA6 emerges as an important hub at the centre of our PC-corr network and it is highly expressed in SCC, hence we speculate that its role can represent a new finding suggested by the PC-corr method for the identification of key factors that explain the strong malignancy of SCC. Our hypothesis seems also supported by the current literature^93^ on the GATA6 oncogenic properties. Indeed GATA6, which we found to be overexpressed in small cell carcinoma cell lines, is also frequently amplified or overexpressed in gastric, esophageal and pancreatic tumor cell lines^93^ that are very aggressive types of cancer.

In general, the PC-corr network provides valuable information in terms of clear expression patterns and functional discriminative modules identification. Instead, the P-value network at the same cut-off (figure not shown) contains too much information and is extremely difficult to investigate, due to the presence of a large amount of nodes and edges (see Venn diagrams in Fig. 7b), and TP63 is absent and consequently its TF module.

However, we compared the P-value and PC-corr networks at cut-offs that allow having approximately the same number of nodes, and we observed that the P-value network (Supplementary Fig. S8) is mainly characterized by single and double interactions, thus losing its module interconnectivity. Moreover, it doesn’t contain CST and the genes related to neuroendocrine differentiation (excluded INSM1), in addition to TP63 and its targeting gene TUBB2B. Therefore, this dataset is an instance where our approach performs better in finding discriminative genes and functional modules. Finally, the PC-corr method in this application showed the power to reverse engineering discriminative network modules, which are clearly functional related, and that represent potential reference for defining new combinatorial network-based multigene markers for carcinoma subtypes categorization.

### Cancer stem cell mechanomics

The regenerative properties of tumors are mainly accounted by cancer stem cells (CSCs), which manifest as recurrence and metastasis. CSCs appear to have multiple growth options, which allow them to adopt different states and, subsequently, contribute to their ability to evade therapy. Therefore, it is crucial to concurrently characterize the molecular and mechanical differences of CSCs under different culture conditions, as a strategy towards precision medicine. As a matter of fact, patient-derived primary cell cultures can represent a reliable benchmark for *in vitro* testing of tailored-to-patient targeted therapies.

Here, cancer stem cell lines derived from the same patients with glioblastoma multiforme were cultured in three different states (FGFJI, EGF and Serum) with the aim to maintain them in different signal transduction states^94^. The dataset consists of 9 samples for each cell culture condition for a total of 27, and nearly 40.000 genes whose expression was assessed by means of genome-wide RNA-seq.

**Figure 8:**
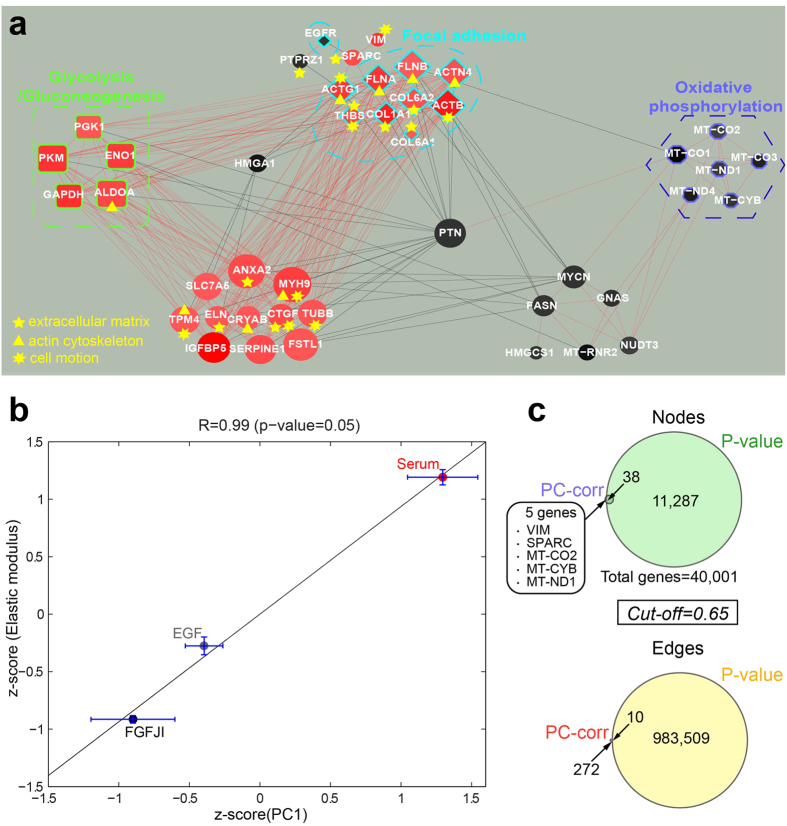
Cancer stem cells gene expression pattern and correlation with cell mechanics. (**a**) PC-corr gene network constructed using cut-off 0.65. The three looped-dashed lines indicate three significant pathways, while yellow different symbols highlight the enriched genes classified as GO terms. (**b**) Linear regression plot of z-score of PC1 and z-score of elastic modulus for each cell culture condition. Each point represent the PC1-score-average vs the elastic-modulus-average of all the samples in the given cell culture condition. The standard error for each computed average is reported as an error bar. (**c**) For cut-off = 0.65, the gene difference between the PC-corr and P-value networks in the nodes and edges are illustrated in the proportional Venn diagrams. Some genes are only present in the PC-corr network, like the genes reported in the box on the left. Only 10 gene interactions present in the PC-corr are present in the P-value network too.

Then PCA analysis was performed and, as shown in suppl. Fig. S9, the three culture conditions were patently distanced from each other along the PC1. Notably, the Serum group (red circles in Fig. S9) is less homogeneous (more unstable conditions) than the two others. To investigate the cause of the discrimination from the systems molecular point of view and detect discriminative network modules related with the PC1, we applied the PC-corr method. Figure 8a shows the PC-corr network (cut-off 0.65), where the red genes are highly expressed in Serum, while the gradient from grey to black indicates genes highly expressed in EGF and FGFJI.

To validate the biological reliability of the gene network modules emerged from the PC-corr analysis, we performed functional annotation analysis (DAVID functional annotation tool, p-value after Benjamini correction <0.05 to detect significant categories) of the genes involved in the modules. The result of this validation was the identification of significantly enriched genes in GO terms and in three KEGG pathways: oxidative phosphorylation, glycolysis/gluconeogenesis and focal adhesion that is known to be associated with cell mechanics. In addition, many cell mechanics enriched genes related with cytoskeleton activity were present (genes labels with yellow triangles in Fig. 8a).

Inspired by the enrichment of the inferred discriminative module for cell mechanics related genes, the morphological variations of the cells under mechanical stress were also measured by means of real-time deformation cytometry (RT-DC) technique^95^. The cells elastic moduli were calculated and in Fig. 8b we investigated the level of linear (Pearson) correlation of PC1 (representing the dimension of discrimination that generated the discriminative network modules) and elastic modulus of the cells (representing cell mechanic changes). Since the mechanical assessment was not done for all the biological replicates and to have generalized indicators, we plotted the PC1 score (x-axis) and elastic modulus (y-axis) as average values (+/− standard error) of all the samples for each culture condition. The same operation was repeated separately for each of the three culture conditions, hence we got three average indicators with standard errors in the Fig. 8b. The final values were z-score-transformed to have a scale-invariant representation. The high correlation (0.99 with p-value = 0.05) demonstrates that the network we extracted accounts well for the cell morpho-mechanics variations, and we suggest that in future experiments it could be proven (by gene silencing or overexpression) to which extent the detected network modules participate to determine the mechanical differences of these cells in the three culture conditions.

In addition, the comparison (using the same cut-off value for both the methods) of the P-value (figure not shown) and PC-corr networks was performed, and the node and edge Venn diagrams show that the P-value network cannot catch two genes (SPARC and VIM) involved in cell motion and extracellular matrix and three genes (MT-CO2, MT-CYB, MT-ND1) of the oxidative phosphorylation pathway (Fig. 8c). On the other hand, although the P-value network is large (11,287 nodes) and highly interconnected (983,509 interactions), it cannot catch almost all the gene interactions present in the PC-corr network. However, if we cross-match the PC-corr network versus a P-value network with a comparable number of genes (only the first 38 genes with the highest p-value selected), the P-value network loses interconnectivity due to a majority of single interactions between couple of genes (Fig. S10). Taken together these results obtained in cancer systems mechanomics show how the PC-corr method can help to unveil molecular discriminative modules that set a connection across different scales in the cell: form the underlying molecular interactions to the macroscopic mechanical phenotypes.

## Discussion

Networks are the method of choice to explore the interactions among several kinds of features (genes, bacteria, metabolites, etc.). Here we proposed a new approach for network inference that merges the commonly used correlation-based association of the features with an unsupervised *multivariate* analysis, i.e. PCA. Exploiting an unsupervised approach allowed to discover hidden patterns in the data without any a priori information on the samples, or to provide an unbiased and unsupervised confirmation of the groups’ differences. Hence, we proposed to clarify the groups’ differences visualized by PCA by means of the PC-corr network inference and the analysis of the functional modules emerging from the PC-corr network structure.

Considering different datasets should be regarded as a point of strength in a study that is addressed to the large community of omic science. We believe that, in order to test how a new approach performs on real omic data, we have to verify its outcome in different fields. Therefore, each section of this study was dedicated to a different omic dataset, and we opted to compare the P-value and PC-corr networks. For the lipidomics dataset, we compared them also to the P-value Mutual Information (MI) network, that appeared highly broken (nonlinear measures are more difficult to be inferred in case of small sample datasets) and characterized by single/double interactions (Fig. 3c), already spotted by the P-value network (Fig. 3d). Since for the first analysed dataset the MI-based inference method did not work in identifying intra-connected or inter-connected lipid sub-modules/modules like in the PC-corr and P-value networks (Fig. 3a,b), we continued comparing the P-value and PC-corr approaches, in relation to the systems biology evidences achieved by the methods’ results in multiple datasets.

We adopted this evidence-based approach on multiple real datasets because unfortunately, to the best of our knowledge, at the state-of-the-art there are not available theoretical models able to simulate different types of artificial omic datasets generated by different technologies. This could be a very interesting project for another study by itself, but it is off topic for this article. Different technological platforms are used to generate omics datasets from different fields (for instance shotgun mass spectrometry for lipidomics, next generation sequencing for metagenomics and SNP data, etc.) and it was crucial to verify if our approach can give meaningful biological results in all these different fields. Finally, the goal of the article is also to represent a point of reference for biologists that want to find a clear example of how to complement their P-value correlation network analysis. We suggest a multivariate method that can extend the PCA sample visualization they frequently adopt, and for which they want to gain a meaningful explanation from the omic feature perspective. We really hope that biologists, interested in fast and easily interpretable methods that clarify the functional omic modules to associate to their PCA sample separation, could use our approach. For example, in the population genetics dataset, PCA could reveal a substructure in the Japanese population that was in reality split in the Tokyotas and Okinawans. It is this ability of PCA to spot the features that play a major role in the discrimination of the samples in the reduced two-dimensional (2D) space (which are responsible for the discrimination in the data) that was transferred in the network structure (PC-corr) and combined with the Pearson correlation-based interactions. Thus, in the PC-corr network, the undirected graph displays edges that account for two levels of meaning, discrimination and correlation, while the nodes represent the features that cause the separation of the samples. This is why we have called it discriminative correlation network, that was proven by LOOCV to be a robust approach because of its unsupervised nature.

Our goal was to test how our discriminative network approach would perform on different *omic* datasets, ranging from lipidomics, to transcriptomics, to metagenomics and genomics. We found modules that can have a biological explanation. Some of them could be expected in the light of the hypothesis that was being tested: in the lipidomic dataset, for example, most of the lipid species are arranged in modules according to their lipid class, and each module presents a specific range in the number of carbon atoms in the fatty acid chains. In some cases modules can be unexpected and become a starting point for new insights, and therefore be a support for decision making in designing new experiments or addressing pilot studies.

The PC-corr networks were always compared to P-value networks. The first strategical difference lies in the way features are selected: while the PC-corr adopts a multivariate approach, i.e. it uses a combination of features that are responsible for the sample discrimination, in the P-value network the discriminating features are singly selected (one by one) with each Mann-Whitney test (followed by Benjamini-Hochberg procedure). The second strategical difference lies in the generation of the correlation weights in the network. PC-corr combines in parallel and at the same time in a unique formula the discrimination power of the PC-loadings and the association power of the Pearson correlation, directly providing in output discriminative omic associations. These are generated using a robust (because we use as merging factor the minimum operator, which is a very penalizing operator) mathematical trade-off between two important factors: multivariate discriminative significance and correlation association. In addition, as mentioned above, the minimum operator works as an AND logical gate in a digital circuit, therefore in order to have a high link weight in the PC-corr network, both the discrimination (the PC-loadings) and the association (the Pearson correlations) of the nodes adjacent to the link should be simultaneously high. Instead, the P-value procedure begins with the pre-selection of the significant omic features and, only in a second separated step, computes the associations between these features. Therefore, in P-value networks, the interaction weights are the result neither of multivariate discriminative significance, nor of a discrimination/association interplay. Considering the different rationale behind these two approaches, we consider them complementary. Sometimes the PC-corr network was able to achieve results when the P-value network was not, like in the carcinoma cell-wide promoteromic dataset. There we discovered a known TF module, that was absent in the other network. Interestingly in the metagenomic example, the pairwise statistical test performed on each bacterium (univariate statistics) gave no significant results, therefore the P-value network by definition did not exist. On the contrary, and surprisingly, a well-defined PC-corr network emerged from the same data. The reason lies also in the ‘multivariate core’ of the PCA that was able to extract a collection of features, which linearly combined all together in the PC2. This discriminative set of feature associations spotted significant differences between the samples before and after PPI treatment. In this case too, like in the lipidomic dataset, we could identify some sub-modules where the nodes (here bacteria) belonged to the same class. Likewise the P-value network sometimes can highlight groups of interacting features that are missing in the PC-corr network, as in the case of the lipids GD1, GD3, GM2, GT1 (Fig. 2f). Interestingly, across the different case studies, in some of them PC-corr networks include much more nodes, in others, P-value networks includes much more nodes. The factor that influences this difference lies in the different methods (univariate against multivariate) that are used to build the correlation networks. In addition, the P-value networks consider the features that are significantly different after Benjamini correction. This multiple hypothesis adjustment depends from the number of features and is an additional factor that generates instability in the number of selected nodes appearing in the P-value networks.

As a strategy to mine the network structure, the operator can display the different PC-corr network modules emerging for different cut-offs and adopt the network representations that better fit the needs and the aim of his/her biological questions. However, like for P-value network thresholding, there is not a general rule to choose the optimal cut-off. For instance, choosing a lower threshold (=0.5) offers a general overview of the discriminative (native) network organization, while a high cut-off (>0.6) allows a better resolution of the discriminative network functional modules. That is to say a focus on the most discriminative associations of features that act as main factors in the generation of the different states of the system under analysis.

The PC-corr network is pruned at a threshold that is specifically informative of the biomedical concept behind the network organization. This is both a weakness and a strength: on one hand, we do not have an impartial criterion for cut-off selection, on the other hand this is the starting point for the development of a new tool. As a matter of fact, the next stage will be to use this tuning parameter (the cut-off) to infer the hierarchy of the modules in the network structure, that is exploring how modules rearrange and/or disaggregate and/or disappear at different cut-offs, following a strategy very similar to percolation analysis in complex network theory. Finally, our datasets did not demonstrate particular problems with sample outliers and in general the pre-processing, using data normalization, can significantly help to reduce the impact of outliers on the PCA projection. However, a future interesting development could be also to consider the option to adopt robust PCA^96^ instead of standard PCA.

The core advantage of our algorithm is to be the first and innovative tool to provide a multivariate-discriminative correlation network associated to PCA analysis. Nowadays, there are many algorithms tailored to reverse engineer a functional network from genomic data but not for omics data in general such as PC-corr does. Network reconstruction techniques can be generally divided into two broad classes: biological knowledge-driven (such as STRING^97^,^98^) or data-driven (such as the above-mentioned *minet*^99^, which for instance can be applied to expression data). However, both these methods only focus on the features’ interactions, neglecting their role in discriminating two or multiple conditions, for instance control versus disease samples in case-control studies. The P-value network approach illustrated in the paper can be used to address this question by building correlation networks between significant features, but it lacks the multivariate selection of discriminating features and, from the results shown in this paper, proved to miss interesting functional modules pointed by PC-corr. In contrast, our proposed approach integrates the discriminative significance of the commonly applied multivariate analysis named PCA with the widely used correlation association by Pearson correlation, to make PC-corr unique in inferring multivariate-discriminative correlation networks. As a result, the obtained network can be of great aid for the user to enlighten the most discriminative and functionally relevant features’ associations, linked to the PCA discrimination of groups of samples. Its main disadvantage is that it only investigates the features’ linear relationships (associations) and neglects the discovery of non-linear relationships between the features. We are currently designing a new algorithm that should overcome this limitation by decoupling linear and nonlinear discriminative associations.

In conclusion, the PC-corr approach results particularly useful for small datasets or pilot studies, where supervised approaches are unfeasible. It proposes to shift the strategy of analysis from the univariate selection of single discriminative features and their correlation, to the multivariate identification of discriminative and collective associations between omic features. PC-corr can thus represent a new tool for the definition of combinatorial and multiscale biomarkers in precision medicine^100^ and for the investigation of complex omic data in systems biomedicine. For instance, in a mechanomic pilot study composed of a small sample-size dataset, PC-corr allowed to detect important functional modules of genes directly correlated to variations of the elastic moduli of cancer cells. This result associates functional modules that emerge at the molecular systems biology micro-scale-level with the cellular mechanic properties that arise at phenotypical meso-scale-level, and represents a significant innovation in computational multiscale systems biology. Likewise, given its machine-learning based computational layout, PC-corr is a general inference-based approach that can be easily adopted for big data exploration in computer science and analysis of complex systems in physics.

## Methods

### Dataset Description

#### Lipidomics

The lipidomic dataset contains absolute (molar) concentrations of 281 lipid species from 27 major lipid classes in the human plasma samples of 36 male, and 35 female healthy young Caucasians, together with their clinical parameters. In addition, information reflecting the use of hormonal contraceptives to the female cohort was provided: the female-contraceptive cohort comprises 19 females^22^.

Lipid identification and quantification was acquired by shotgun lipidomics and liquid chromatography tandem mass spectrometry (LC-MS/MS)^22^.

#### Metagenomics

The metagenomic dataset was generated by Amir and colleagues^29^, and is public and available in the MG RAST database (http://metagenomics.anl.gov/linkin.cgi?project=5732). It comprises eight patients, whose gastric fluid was sampled at two different time points, that is before PPI treatment, and after eight weeks of PPI treatment, for a total of 16 samples. Metagenomes were obtained by pyrosequencing fragments of the 16S r-RNA gene on the GS FLX system (Roche). Then the data were processed, replicating the bioinformatics workflow followed by Amir and colleagues^29^ in order to obtain the matrix of the bacterial absolute abundance.

Sequence reads were analysed with the pipeline QIIME Quantitative Insights into Microbial Ecology v. 1.6.0^101^ using default parameters: sequences were removed if shorter than 200 nt, if they contained ambiguous bases or uncorrectable barcodes, or if the primer was missing. Operational Taxonomic Units (OTUs), that is clusters of sequences showing a pairwise similarity no lesser than 97%, were identified using the UCLUST algorithm (http://www.drive5.com/usearch/). The most abundant sequence in each cluster was chosen as the representative of its OTU, and this representative set of sequences was then used for taxonomy assignment by means of the Bayesian Ribosomal Database Project classifier^102^ and aligned with PyNAST^103^. Chimeras, that is PCR artifacts, were identified using ChimeraSlayer^104^ and removed.

The Greengenes database, which was used for the annotation of the reads, additionally identifies groups of bacteria that are supported by whole genome phylogeny, but are not yet officially recognized by the Bergeys taxonomy, which is the reference taxonomy, and is based on physiochemical and morphological traits. This results in a special annotation for some taxa, like *Prevotella* (Fig. 3), that thus appears both with the general annotation, that is *Prevotella*, and with the special annotation, that is between square brackets, [*Prevotella*].

#### Developmental genomics

The developmental genomic dataset contains quantitative (normalized) mRNA profiles of all Human TFs using quantitative Reverse Transcription PCR (qRT-PCR) across 32 tissues (excluding monocyte cell lines)^105^. In PCA, the tissues were labelled according to the predominant germ layer they derive from (as explained in the Germ layer attribution section): ectoderm, mesoderm or endoderm.

#### Population genetics

The genotype data used in this paper was taken from the Pan-Asian SNP Consortium Database (PanSNPdb), comprising 75 populations (71 Pan-Asian and 4 from the HapMap Project) with 1,928 individuals and 54,794 SNPs on autosomal chromosomes^52^,^53^. We focused on the Japanese population, that comprises 164 individuals from two sub-populations: 115 individuals from Tokyo (the JPT and JP-ML ethnic groups) indicated with JP-Tk, and 49 Japanese Ryukyuan from Okinawa (the JP-RK ethnic group)^54^. Each individual was analysed using a set of 54,794 autosomal SNPs. SNPs data were converted to a matrix by recoding genotypes as follows: 0 for homozygous wild-types, 1 for heterozygotes, 2 for homozygous variant types and 3 for missing values.

Missing values were replaced in each of the 54,794 SNP by the mode of each specific SNP, as previously done in ref. 52, since the proportion of missing values was small with respect to the data size (0.73%)^54^,^106^.

#### Carcinoma cell-wide promoteromic dataset

The complete expression dataset was downloaded from the repository of the FANTOM5 project (download date: 6 October 2014). FANTOM5 carried out the Cap analysis of gene expression (CAGE) across 975 human and 399 mouse samples, including primary cells, tissues and cell lines, using single-molecule sequencing^74^. CAGE is a high throughput molecular biology sequencing technique that measures RNA expression by sequencing small fragments or tags of about 27 nucleotides of the 5′ end of capped RNA molecules^72^. In our analysis, we focused on human small-cell and squamous-cell carcinoma-derived cell line samples (respectively 7 and 9 samples), with 184,827 ‘robust’ CAGE peaks which were obtained by decomposition-based peak identification (DPI). Only 131,950 of these peaks were expressed in at least one of these 16 carcinoma samples. By removing the peaks without explicit gene annotations, 71,189 peaks were obtained, corresponding to 22,224 known genes since multiple CAGE peaks point to one gene. Our PCA and the subsequent network construction were carried out on these 71,189 different CAGE peaks, focusing on human small-cell and squamous-cell carcinoma-derived cell lines (7 and 9 samples respectively).

#### Cancer stem cell mechanomics

Three different cancer stem cell lines from three patients with glioblastoma multiforme were cultured in three different states (FGFJI, EGF and Serum)^93^, each characterized by different levels of STAT3 phosphorylation and Hes3 expression. The cell lines were previously established from acutely resected human tumor tissues and all human tissues were obtained during surgical resections from patients with newly diagnosed or recurrent tumors. After cell culture in the three states, genome-wide RNA-seq was performed for each cell line of every culture state with triple replicate (totally 27 samples) on an Illumina HiSeq 2500, resulting in 40,001 sequenced genes. Normalization of the raw read counts of each gene was based on the library size. Real-time deformability cytometry (RT-DC)^94^ experiments were also performed for each cell line. RT-DC allows for continuous mechanical characterization of cells and the cell’s elastic modulus was calculated applying an analytical model assuming the cell as a linear elastic object^95^.

#### Construction of discriminative correlation networks based on the PC-corr formula

In order to find discriminative network modules in high-dimensional datasets based on an unsupervised analysis, we developed a data-driven method to construct correlation networks according to a prior Principal Component Analysis (PCA)^13^,^107^, that reveals which features are responsible for the discrimination of two or multiple conditions. This easy and fast method, explained in Figure 1 (algorithm shown in Supplementary Table S1), infers discriminative linear associations between features and can underline discriminative network modules applying different thresholds to the network interactions.

#### Unsupervised analysis by PCA and processing of the loadings for the PC-corr network

Since this new approach is a merging of PCA analysis and correlation network, the first step for the network construction consists in performing PCA (centred or non-centred) for dimensionality reduction, after normalizing the dataset, to discover samples’ linear relationships hidden in the multidimensional space. In PCA, the dataset is transformed into a new coordinate system through an orthogonal linear transformation and can be visualized in the two-dimensional space, where the first dimension (Principal component 1 or PC1) and the second dimension (Principal component 2 or PC2) are linear combinations of the original features with a certain weight or loading for each feature^13^,^107^.

PCA can unsupervisedly detect two (or more) distinct groups of samples and, after assigning the labels, such grouping can coincide with the different conditions (for example control and disease samples). In some cases, the distinct cohorts are clearly separated along PC1 or PC2 (PCn) while, when there is no clear separation, a pairwise non-parametric test like Mann-Whitney can be performed for both dimensions, to assess whether there is a significant discrimination.

Briefly, if the separation into two or more groups is significant along a PCn, or PCA can clearly reveal their presence, we extract the loadings from the most discriminative dimension for the construction of the network. Since the discriminative correlation network couples a preliminary analysis by PCA with the correlation between features to explore discriminative feature modules, it was named the PC-corr network.

The PCn loadings should be pre-processed with a procedure so that their distribution can be comparable with the Pearson correlation coefficients of the features. The issue is not trivially solved by scaling the loadings in the interval [−1,1]. Therefore, we investigated this by experimentally using the data from the different datasets to which the algorithm was applied. Since in omics science we have high-dimensional datasets, we found that the pairwise Pearson correlation coefficients of the features are approximately normal in distribution. For instance, with only 100 features we already have 4,950 pairwise correlations, and for the law of large numbers we noticed that the related distribution has a negligible skewness and is well spread in the interval [−1,1]. On the other hand, we noticed that the distribution of the PC loadings was varying significantly across the datasets as well as the related skewness. In addition, since the sum of the squared loadings of a PC is equal to one, the original PC loading values are not scaled between [−1,1]. This distribution diversity is relevant because these two factors (loadings and Pearson correlation) are combined using a minimum operator and, depending on how these two factors are distributed over the interval, one might have a stronger influence when combining them. Here, we discuss the different solutions that we explored:

1. We tried to normalize both the PC loadings and the pairwise correlations using the z-score function. The goal was to center both the distributions around their mean value and to scale them in the same range of variance. This strategy did not work because it did not stabilize the high skewness variability of the PC loadings distribution across different datasets.
2. We applied Box-Cox transformation to the PC-loadings, followed by their scaling in the interval [−1,1]. The Box-Cox transforms non-normally distributed data to a set of data that has approximately normal distribution. The Box-Cox transformation is a family of power transformations. This strategy did not gain stable results: on some datasets working fine and on others giving unsatisfactory performance.
3. We tried to apply a quantile-normalization-like function to force the two distributions to be identical. In addition, we also considered to use bootstrapping in order to normalize the theoretical bootstrapping-adjusted distribution and improve the accuracy in the normalization. This strategy was effective but it required some tricks to adjust the size of the loadings and Pearson correlation vectors that were originally of different length. The issue was solved using a nonparametric Kernel smoothing function for the estimation of the theoretical distributions (as we mentioned above we tried using also bootstrapping) and applying the quantile normalization to the inferred distribution. The problem of this strategy was the computational cost that for large datasets with many features was overwhelming in comparison to the expected performance.
4. We designed a *heuristic function*, inspired to a lognormal function, to normalize the original PC loading values with the aim to reduce their skewness, approximate and force their distribution towards normality, stabilize their variance. Then we applied a second function for scaling the values in the entire interval [−1,1]. This was very effective, we gained results similar to the strategy of point 3 above with the advantage of a significantly reduced computational cost and time.

All these tests were done at the beginning of the project and took around two months of investigations.

The heuristic function discussed at point 4 above is:


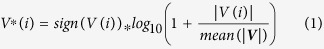


where ***V*** represents the vector of all loadings with the i-th element *V*(*i*), corresponding to the i-th feature, and V^*^(*i*) is the normalized loading of the i-th feature. Precisely, 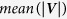 is the average of all the absolute loadings’ values. This is a lognormal inspired function with the introduction of a scaling factor that is automatically tuned using the 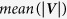.

Secondly, we need to scale all the normalized loadings to distribute the new loadings *V*^*new*^ on the interval[−1,1], as follows:


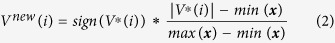


where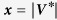 (the vector containing all the absolute values of the normalized loadings).

#### Construction of the PC-corr network

In the PC-corr network, the edge’s weight is based not only on the Pearson correlation coefficient between two features, like in co-expression networks, but also on the PCA loadings of the two interacting features in order to search for discriminative correlations. However, its sign rather reflects the positive or negative correlation between two features.

Hence, for two given nodes *i* and *j* we calculated their edge value as


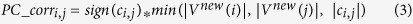


where *c*_*i,j*_ is the Pearson correlation coefficient of features *i* and *j* and 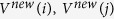 are the normalized and scaled loadings of features *i* and *j* respectively.

Thence, we impose a cut-off between 0 and 1 (usually larger or equal to 0.5) to select the edges that have 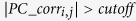, and construct a weighted adjacency matrix after removing all single nodes (without interactions). Note that the cut-off is selected by the operator in relation to his/her needs to explore the network structure and organization at different levels.

With regard to the network’s nodes, each one represents a feature and is associated to a value, its corresponding processed loading *V*^*new*^ that can be negative or positive and be associated to a node colour. Lastly, the method outputs two tables that are required to visualize the network: the network table with the PC-corr values for the edges’ attributes, and a loadings’ and colour table associated to the nodes. Then the resulting network, thus composed by discriminative multifactor feature modules that were derived from PCA, can be displayed by a default visualization tool, or by an open source platform such as Cytoscape (http://www.cytoscape.org/)^108^,^109^ that was used for this study.

In the network representation, the size of each node is proportional to the degree, i.e. to the number of its interactions with other nodes. Nodes’ colours range from black or red to white, with a gradient depending on the loading’s weight. The node colour recalls the colours used in the PCA plot: red nodes indicate the features that have higher values in the samples represented by red dots, while black nodes correspond to the features that have higher values in the samples represented by black dots.

A node is red as specified by the node colour, and shifts to lighter shades of red when its loading weight approaching 0, that corresponds to white nodes; likewise a black node shifts to lighter shades of grey as the loading value comes nearer to 0. The node colour concurrently expresses the behaviour of the features in the system under analysis: antithetical colours, i.e. red and black, indicate features that behave in opposite ways, while nodes characterised by the same colour range (red to pink or black to grey) correspond to features that display the same behaviour (for instance their abundance either increases or decreases).

Moreover, a red edge links nodes that display the same behaviour (positive correlation) and have therefore the same colour, while a black edge connects nodes that are negatively correlated (nodes with antithetical colours).

#### Construction of the P-value network

The P-value network is a variation of the classical correlation-based network: it is a correlation network, but considers only the features that are significantly different when comparing the groups of samples under analysis. A pairwise Mann-Whitney test is performed for each feature on the normalized dataset (the same normalization used before applying PCA), and p-values are adjusted using Benjamini*-*Hochberg correction. Only the most significant features (p-values after correction < = 0.05) are considered for the construction of the correlation network.

Hence, in the P-value network the edge value corresponds to the Pearson correlation coefficients between significantly selected features, while the node colour is given by the difference between the medians of the features in the two groups, in accordance with the Mann-Whitney test (test on medians) performed for each feature.

Finally, the network is cut at different thresholds, i.e. at the same cut-off of the PC-corr network, and, for big networks, it is also cut at the cut-off level that gives approximately the same number of nodes of the PC-corr network.

For big datasets, the P-value network with the same cut-off of the PC-corr network is not shown due to the excessively high number of nodes and interactions. We created Venn diagrams of the nodes and of the edges for every dataset, to emphasize the similarities and differences between the two types of networks.

#### Construction of the P-value MI network

The P-value MI network is a mutual information (MI) network, but considers only the features that are significantly different when comparing the groups of samples under analysis.

Analogously to the P-value network, a pairwise Mann-Whitney test is performed for each feature on the normalized dataset (the same normalization used before applying PCA), and p-values are adjusted using Benjamini*-*Hochberg correction. Only the most significant features (p-values after correction < = 0.05) are considered for the construction of the MI network.

The MI network between significantly selected features was then constructed using *minet*^92^ (https://www.bioconductor.org/packages/release/bioc/html/minet.html), a R/Bioconductor package which provides a set of functions to infer MI networks from a dataset. We considered all the inference algorithms (CLR, ARACNE, MRNET, MRNETB), using for MI computation Pearson correlation (and other default values).

As in the P-value network, the inferred MI network has coloured nodes colour based on the difference between the medians of the features in the two groups, in accordance with the Mann-Whitney test (test on medians) performed for each feature.

Finally, they are cut at the same cut-off of the PC-corr and P-value networks and just the algorithm giving the higher number of interactions, that we called P-value MI, was studied in comparison to the other two approaches.

Since for the lipidomics dataset the P-value MI network (obtained with the CLR algortithm) showed no interesting result, we continued our analysis by comparing the PC-corr network to the P-value network.

#### Functional annotation analysis by DAVID

The David^80^,^81^ functional annotation tool (https://david.ncifcrf.gov/) was used to provide a functional interpretation of the large list of genes obtained from the genomic studies under analysis.

Some enriched biological themes, especially KEGG and PANTHER pathways and GO terms, (p-value after Benjamini-Hochberg correction <0.05 or 0.01) we identified were included in the representation of the PC-corr network. We performed the enrichment analysis using the entire gene list of the dataset as background and not all the genes were recognized.

#### Ensembl

All information regarding single nucleotide polymorphisms (SNPs), including type of variant, genomic position, gene consequences and significant associations to phenotypes, were acquired through Ensembl (http://www.ensembl.org).

The Ensembl Variation database stores, for several species, the areas of the genome that differ between individual genomes (“variants”) and, where available, associated phenotype information (traits and disease).

#### Germ layer attribution

The following background information was considered when analysing the developmental genomic dataset. The embryo of all triploblastic animals presents three distinct primary layers of cells, called germ layers, that give rise to specific tissues and organ systems. Overall ectoderm, the outermost germ layer, produces the surface layer of the skin (epidermis) and forms the brain and the nervous system; endoderm, the inner layer, differentiates to form the epithelial lining of the digestive and respiratory tracts with their associated organs, and the prostate, the thyroid and parathyroid glands, the thymus gland, the urinary bladder and part of the urethra^110^,^111^.

Differently mesoderm, the middle germ layer, gives rise to the circulatory system (including the heart and the spleen), blood and lymphatic vessels, muscles (smooth, cardiac and skeletal muscles), connective tissues, adipose tissue, bones, dermis and subcutaneous layer of the skin, the kidneys and gonads^112^. However, tissues can have more than one attribution, like the skin, which has both mesodermal (dermis) and ectodermal (epidermis) contributions^113^.

#### Leave-one-out-cross-validation

To verify the robustness of our approach we used leave-one-out cross-validation (LOOCV).

1. First we analysed all the samples by PCA and obtained the loadings of the most discriminative dimension, which were used for the construction of the PC-corr network.
2. PCA analysis was then repeated iteratively over the total number of samples for each leave-one-out cross-validation of the dataset. At each iteration one sample was removed from the normalized dataset before performing PCA, and then put back after the analysis was done.
3. During the procedure, a loadings matrix was created: the first column contained the loadings of the most discriminative dimension obtained from the PCA of the original data, while the remaining columns were filled with the loadings of the most discriminative dimension of the PCA performed after removing each time a different sample.
4. Finally, after applying the PC-corr expression to the PCA loadings and the features’ correlation coefficients of each iteration, the PC-corr matrix obtained from the original omic dataset was combined with all the PC-corr matrices created upon sample removal.
5. The different approaches used to combine the PC-corr networks were: average, median and minimum (in absolute value), where the latter approach actually represents the intersection network. Specifically, the combined network contains an edge, whose value is given by the average/median/minimum (in absolute value for the minimum) of the values of that interaction in all the PC-corr matrices previously obtained.
6. As a result, the average/median/minimum network is obtained under the cut-off adopted in the original PC-Corr.
7. Venn diagrams were created to compare the four networks with respect to number of nodes and edges, using the interacting tool Venny 2.1.0^114^ (http://bioinfogp.cnb.csic.es/tools/venny/).

In addition, in order to reduce the time of computation, we selected only the features whose maximum absolute value in the loading matrix was higher than the chosen cut-off.

### Ethics approval and consent to participate

 Not applicable because the used datasets have been generated by previous biomedical studies, for which ethics approvals and consents were formerly collected.

### Availability of data and materials

 The PC-corr MATLAB code and its user guide is available at the following GitHub repository: https://github.com/biomedical-cybernetics/PC-corr_net. Creation of R and Pyton codes are in progress. A user-friendly on-line web service running the PC-corr algorithm is under construction.

## Additional Information

**How to cite this article**: Ciucci, S. *et al*. Enlightening discriminative network functional modules behind Principal Component Analysis separation in differential-omic science studies. *Sci. Rep.* **7**, 43946; doi: 10.1038/srep43946 (2017).

**Publisher's note:** Springer Nature remains neutral with regard to jurisdictional claims in published maps and institutional affiliations.

## Supplementary Material

Supplementary Information

## References

[b1] Marbach D. . Revealing strengths and weaknesses of methods for gene network inference. Proc. Natl. Acad. Sci. USA. 107, 6286–91 (2010). 20308593 10.1073/pnas.0913357107 PMC2851985

[b2] Bansal M., Belcastro V., Ambesi-Impiombato A. & di Bernardo D. How to infer gene networks from expression profiles. Mol. Syst. Biol. 3, 78 (2007). 17299415 10.1038/msb4100120 PMC1828749

[b3] Gardner T. S. & Faith J. J. Reverse-engineering transcription control networks. Phys. Life Rev. 2, 65–88 (2005). 20416858 10.1016/j.plrev.2005.01.001

[b4] Cannistraci C. V., Alanis-Lobato G. & Ravasi T. From link-prediction in brain connectomes and protein interactomes to the local-community-paradigm in complex networks. Sci. Rep. 3, 1–13 (2013). 10.1038/srep01613 PMC3619147 23563395

[b5] Cannistraci C. V., Alanis-Lobato G. & Ravasi T. Minimum curvilinearity to enhance topological prediction of protein interactions by network embedding. Bioinformatics 29, 199–209 (2013). 10.1093/bioinformatics/btt208 PMC3694668 23812985

[b6] Daminelli S., Thomas J. M., Durán C., Vittorio Cannistraci C. & Alessio M. Common neighbours and the local-community-paradigm for topological link prediction in bipartite networks. New J. Phys. 17, (2015).

[b7] Isik Z., Baldow C., Cannistraci C. V. & Schroeder M. Drug target prioritization by perturbed gene expression and network information. Sci. Rep. 5, 17417 (2015). 26615774 10.1038/srep17417 PMC4663505

[b8] Cannistraci C. V. . Pivotal role of the muscle-contraction pathway in cryptorchidism and evidence for genomic connections with cardiomyopathy pathways in RASopathies. BMC Med. Genomics 6, 5 (2013). 23410028 10.1186/1755-8794-6-5 PMC3626861

[b9] Ruan J., Dean A. K. & Zhang W. A general co-expression network-based approach to gene expression analysis: comparison and applications. BMC Syst. Biol. 4, 8 (2010). 20122284 10.1186/1752-0509-4-8 PMC2829495

[b10] Zhang B. & Horvath S. A general framework for weighted gene co-expression network analysis. Stat. Appl. Genet. Mol. Biol. 4, Article17 (2005). 10.2202/1544-6115.1128 16646834

[b11] Marbach D., Costello J., Küffner R. & Vega N. Wisdom of crowds for robust gene network inference. Nat. Methods 9, 796–804 (2012). 22796662 10.1038/nmeth.2016 PMC3512113

[b12] Smialowski P., Frishman D. & Kramer S. Pitfalls of supervised feature selection. Bioinformatics 26, 440–443 (2009). 19880370 10.1093/bioinformatics/btp621 PMC2815655

[b13] Ringnér. What is principal component analysis? Nat. Biotechnol. 26, 303–304 (2008). 18327243 10.1038/nbt0308-303

[b14] Cannistraci C. V., Ravasi T., Montevecchi F. M., Ideker T. & Alessio M. Nonlinear dimension reduction and clustering by minimum curvilinearity unfold neuropathic pain and tissue embryological classes. Bioinformatics 26, 531–539 (2010). 10.1093/bioinformatics/btq376 PMC2935424 20823318

[b15] Zagar L. . Stage prediction of embryonic stem cell differentiation from genome-wide expression data. Bioinformatics 27, 2546–2553 (2011). 21765096 10.1093/bioinformatics/btr422

[b16] Bellman R. Dynamic Programming. Princeton University Press: Princeton New Jersey, 70 (1957).

[b17] Erhan D., Courville A. & Vincent P. Why Does Unsupervised Pre-training Help Deep Learning? J. Mach. Learn. Res. 11, 625–660 (2010).

[b18] Basnet K. Centering of data in Principal Component Analysis in Ecologicol Ordination. Tribhuvan University Journal 16 (1993).

[b19] Jolliffe I. T. Principal Component Analysis, Second Edition. Springer Ser. Stat. 98, 487 (2002).

[b20] Shevchenko A. & Simons K. Lipidomics: coming to grips with lipid diversity. Nat. Rev. Mol. Cell Biol. 11, 593–598 (2010). 20606693 10.1038/nrm2934

[b21] Surma M. A. . An automated shotgun lipidomics platform for high throughput, comprehensive, and quantitative analysis of blood plasma intact lipids. Eur. J. Lipid Sci. Technol. 117, 1540–1549 (2015). 26494980 10.1002/ejlt.201500145 PMC4606567

[b22] Sales S. . Gender, Contraceptives and Individual Metabolic Predisposition Shape a Healthy Plasma Lipidome. Sci. Rep. 6, 27710 (2016). 27295977 10.1038/srep27710 PMC4906355

[b23] Bytzer P. & Talley N. J. Dyspepsia. Ann. Intern. Med. 134, 815–822 (2001). 11346316 10.7326/0003-4819-134-9_part_2-200105011-00004

[b24] Lodato F. . Adverse effects of proton pump inhibitors. Best Pract. Res. Clin. Gastroenterol. 24, 193–201 (2010). 20227032 10.1016/j.bpg.2009.11.004

[b25] Chapman D. B., Rees C. J., Lippert D., Sataloff R. T. & Wright S. C. Adverse effects of long-term proton pump inhibitor use: A review for the otolaryngologist. J. Voice 25, 236–240 (2011). 20144536 10.1016/j.jvoice.2009.10.015

[b26] Vesper B. J. . The effect of proton pump inhibitors on the human microbiota. Curr. Drug Metab. 10, 84–89 (2009). 19149516 10.2174/138920009787048392

[b27] Wu W. M., Yang Y. S. & Peng L. H. Microbiota in the stomach: new insights. J. Dig. Dis. 15, 54–61 (2014). 24245792 10.1111/1751-2980.12116

[b28] Williams C. & McColl K. E. L. Review article: Proton pump inhibitors and bacterial overgrowth. Aliment. Pharmacol. Ther. 23, 3–10 (2006). 10.1111/j.1365-2036.2006.02707.x 16393275

[b29] Amir I., Konikoff F. M., Oppenheim M., Gophna U. & Half E. E. Gastric microbiota is altered in oesophagitis and Barrett’s oesophagus and further modified by proton pump inhibitors. Environ. Microbiol. 16, 2905–2914 (2014). 24112768 10.1111/1462-2920.12285

[b30] Tsuda A. . Influence of Proton-Pump Inhibitors on the Luminal Microbiota in the Gastrointestinal Tract. Clin. Transl. Gastroenterol. 6, e89 (2015). 26065717 10.1038/ctg.2015.20 PMC4816248

[b31] Jackson M. A. . Proton pump inhibitors alter the composition of the gut microbiota. Gut 65, 749–756 (2016). 26719299 10.1136/gutjnl-2015-310861 PMC4853574

[b32] Rosen R. . 16S community profiling identifies proton pump inhibitor related differences in gastric, lung, and oropharyngeal microflora. J. Pediatr. 166, 917–923 (2015). 25661411 10.1016/j.jpeds.2014.12.067 PMC4380592

[b33] Paroni Sterbini F. . Effects of Proton Pump Inhibitors on the Gastric Mucosa-Associated Microbiota in Dyspeptic Patients. Appl. Environ. Microbiol. 82, 6633–6644 (2016). 27590821 10.1128/AEM.01437-16 PMC5086557

[b34] Kuang J.-L. L. . Contemporary environmental variation determines microbial diversity patterns in acid mine drainage. ISME J. 7, 1038–50 (2013). 23178673 10.1038/ismej.2012.139 PMC3635239

[b35] Sanduleanu S., Jonkers D., De Bruine A., Hameeteman W. & Stockbrügger R. W. Non-Helicobacter pylori bacterial flora during acid-suppressive therapy: Differential findings in gastric juice and gastric mucosa. Aliment. Pharmacol. Ther. 15, 379–388 (2001). 11207513 10.1046/j.1365-2036.2001.00888.x

[b36] Bik E. M. Molecular analysis of the bacterial microbiota in the human stomach. Proc. Natl. Acad. Sci. USA 103, 732–737 (2006). 16407106 10.1073/pnas.0506655103 PMC1334644

[b37] Li X. X. . Bacterial microbiota profiling in gastritis without Helicobacter pylori infection or non-steroidal anti-inflammatory drug use. PLoS One 4, e7985 (2009). 19956741 10.1371/journal.pone.0007985 PMC2776972

[b38] Costalonga M. & Herzberg M. C. The oral microbiome and the immunobiology of periodontal disease and caries. Immunol. Lett. 162, 22–38 (2014). 25447398 10.1016/j.imlet.2014.08.017 PMC4346134

[b39] Belda-Ferre P. . The oral metagenome in health and disease. ISME J. 6, 46–56 (2012). 21716308 10.1038/ismej.2011.85 PMC3246241

[b40] Yu X., Lin J., Zack D. J. & Qian J. Computational analysis of tissue-specific combinatorial gene regulation: Predicting interaction between transcription factors in human tissues. Nucleic Acids Res. 34, 4925–4936 (2006). 16982645 10.1093/nar/gkl595 PMC1635265

[b41] Ravasi T. . An Atlas of Combinatorial Transcriptional Regulation in Mouse and Man. Cell 140, 744–752 (2010). 20211142 10.1016/j.cell.2010.01.044 PMC2836267

[b42] Neph S. . Circuitry and dynamics of human transcription factor regulatory networks. Cell 150, 1274–1286 (2012). 22959076 10.1016/j.cell.2012.04.040 PMC3679407

[b43] Zorn A. M. & Wells J. M. Vertebrate endoderm development and organ formation. Annu. Rev. Cell Dev. Biol. 25, 221–51 (2009). 19575677 10.1146/annurev.cellbio.042308.113344 PMC2861293

[b44] Komiya Y. & Habas R. Wnt signal transduction pathways. Organogenesis 4, 68–75 (2008). 19279717 10.4161/org.4.2.5851 PMC2634250

[b45] Osumi N., Shinohara H., Numayama-Tsuruta K. & Maekawa M. Concise review: Pax6 transcription factor contributes to both embryonic and adult neurogenesis as a multifunctional regulator. Stem Cells 26, 1663–72 (2008). 18467663 10.1634/stemcells.2007-0884

[b46] Hevner R. F. . Tbr1 Regulates Differentiation of the Preplate and Layer 6. Neuron 29, 353–366 (2001). 11239428 10.1016/s0896-6273(01)00211-2

[b47] Corti V. . Protein fingerprints of cultured CA3-CA1 hippocampal neurons: comparative analysis of the distribution of synaptosomal and cytosolic proteins. BMC Neurosci. 9, 36 (2008). 10.1186/1471-2202-9-36 PMC2324106 18402664

[b48] Bredenkamp N., Seoighe C. & Illing N. Comparative evolutionary analysis of the FoxG1 transcription factor from diverse vertebrates identifies conserved recognition sites for microRNA regulation. Dev. Genes Evol. 217, 227–233 (2007). 17260156 10.1007/s00427-006-0128-x

[b49] Garte S. Human population genetic diversity as a function of SNP type from HapMap data. Am. J. Hum. Biol. 22, 297–300 (2010). 19743305 10.1002/ajhb.20984

[b50] Jobling M. A., Hurles M. E. & Tyler-Smith C. Human Evolutionary Genetics: origins, peoples and disease. Am. J. Hum. Genet. 76, 1087–1088 (2003).

[b51] Balaresque P. L., Ballereau S. J. & Jobling M. A. Challenges in human genetic diversity: Demographic history and adaptation. Hum. Mol. Genet. 16, R134–9 (2007). 17911157 10.1093/hmg/ddm242

[b52] Material S. O., Web S., Press H., York N. & Nw A. Mapping Human Genetic Diversity in Asia. Science (80-.). 1541, 1541–1546 (2011).

[b53] Ngamphiw C. . PanSNPdb: The Pan-Asian SNP Genotyping Database. PLoS One 6, 1–7 (2011). 10.1371/journal.pone.0021451 PMC3121791 21731755

[b54] Alanis-Lobato G., Cannistraci C. V., Eriksson A., Manica A. & Ravasi T. Highlighting nonlinear patterns in population genetics datasets. Sci Rep 5, 8140 (2015). 25633916 10.1038/srep08140 PMC4311249

[b55] Wall J. D. & Pritchard J. K. Haplotype blocks and linkage disequilibrium in the human genome. Nat. Rev. Genet. 4, 587–597 (2003). 12897771 10.1038/nrg1123

[b56] Samaras T. T. & Elrick H. Height, body size, and longevity: is smaller better for the human body? West. J. Med. 176, 206–8 (2002). 12016250 10.1136/ewjm.176.3.206 PMC1071721

[b57] Ross P. D. . A comparison of hip fracture incidence among native Japanese, Japanese Americans, and American Caucasians. Am J Epidemiol 133, 801–809 (1991). 2021147 10.1093/oxfordjournals.aje.a115959

[b58] Willcox B. J., Willcox D. C. & Suzuki M. Demographic, Phenotypic, and Genetic Characteristics of Centenarians in Okinawa and Japan: Part 1 − Centenarians in Okinawa. Mech. Ageing Dev. doi: 10.1016/j.mad.2016.11.001 (2016). 27845177

[b59] Suzuki M., Wilcox B. J. & Wilcox C. D. Implications from and for food cultures for cardiovascular disease: Longevity. Asia Pac. J. Clin. Nutr. 10, 165–171 (2001). 11710359 10.1111/j.1440-6047.2001.00219.x

[b60] Chan Y. C., Suzuki M. & Yamamoto S. Dietary, anthropometric, hematological and biochemical assessment of the nutritional status of centenarians and elderly people in Okinawa, Japan. J. Am. Coll. Nutr. 16, 229–235 (1997). 9176829 10.1080/07315724.1997.10718679

[b61] Willcox D. C., Willcox B. J., Hsueh W. C. & Suzuki M. Genetic determinants of exceptional human longevity: Insights from the Okinawa centenarian study. Age 28, 313–332 (2006). 22253498 10.1007/s11357-006-9020-x PMC3259160

[b62] Donlon T. A. . FOXO3 gene variants and human aging: Coding variants may not be key players. Journals Gerontol. - Ser. A Biol. Sci. Med. Sci. 67, 1132–1139 (2012). 10.1093/gerona/gls067 PMC3668389 22459618

[b63] Morris B. J., Willcox D. C., Donlon T. A. & Willcox B. J. FOXO3: A Major Gene for Human Longevity - A Mini-Review. Gerontology 61, 515–525 (2015). 25832544 10.1159/000375235 PMC5403515

[b64] Willcox D. C., Willcox B. J., Todoriki H. & Suzuki M. The Okinawan diet: health implications of a low-calorie, nutrient-dense, antioxidant-rich dietary pattern low in glycemic load. J. Am. Coll. Nutr. 28 Suppl, 500S–516S (2009). 20234038 10.1080/07315724.2009.10718117

[b65] Everitt A. V. . Dietary approaches that delay age-related diseases. Clinical interventions in aging 1, 11–31 (2006). 18047254 10.2147/ciia.2006.1.1.11 PMC2682451

[b66] Willcox B. J., Willcox C. D. & Suzuki M. The Okinawa Way: How to improve your health and longevity dramatically. (Penguin: UK, 2013).

[b67] Álvarez-Fernández S. M. . Serological immune response against ADAM10 pro-domain is associated with favourable prognosis in stage III colorectal cancer patients. Oncotarget, doi: 10.18632/oncotarget.11181 (2016). 10.18632/oncotarget.11181 PMC5346771 27517630

[b68] Berman J. J. Tumor classification: molecular analysis meets Aristotle. BMC Cancer 4, 10 (2004). 15113444 10.1186/1471-2407-4-10 PMC415552

[b69] Hann C. L. & Rudin C. M. Management of small-cell lung cancer: incremental changes but hope for the future. Oncol. (willist. Park). 22, 1486–1492 (2008). PMC4124612 19133604

[b70] Murray N. & Turrisi A. T. A Review of First-Line Treatment for Small-cell Lung Cancer. J. Thorac. Oncol. 1, 270–278 (2006). 17409868 10.1016/s1556-0864(15)31579-3

[b71] Weinberg R. A. The Biology of Cancer. Garland Science, Taylor & Francis Group (Garland science, 2007).

[b72] Kodzius R. . CAGE: cap analysis of gene expression. Nat. Methods 3, 211–222 (2006). 16489339 10.1038/nmeth0306-211

[b73] Takahashi H., Kato S., Murata M. & Carninci P. In Methods in Molecular Biology (eds. Deplancke B. & Gheldof N.) 786, 181–200 (Humana Press, 2012). 21938627 10.1007/978-1-61779-292-2_11 PMC4094367

[b74] The FANTOM Consortium and the RIKEN PMI and CLST (dgt). A promoter-level mammalian expression atlas. *Nature* **507,** 462–470 (2014). 10.1038/nature13182 PMC4529748 24670764

[b75] Kawase A. . Differences between squamous cell carcinoma and adenocarcinoma of the lung: Are adenocarcinoma and squamous cell carcinoma prognostically equal? Jpn. J. Clin. Oncol. 42, 189–195 (2012). 22210923 10.1093/jjco/hyr188

[b76] Noguchi M. . Small adenocarcinoma of the lung: Histologic characteristics and prognosis. Cancer 75, 2844–2852 (1995). 7773933 10.1002/1097-0142(19950615)75:12<2844::aid-cncr2820751209>3.0.co;2-#

[b77] Lindström A. K. . Discrepancies in expression and prognostic value of tumor markers in adenocarcinoma and squamous cell carcinoma in cervical cancer. Anticancer Res. 29, 2577–2578 (2009). 19596931

[b78] Ladanyi M. & Pao W. Lung adenocarcinoma: guiding EGFR-targeted therapy and beyond. Mod. Pathol. 21 Suppl 2, S16–22 (2008). 18437168 10.1038/modpathol.3801018

[b79] Wiedenmann B., Franke W. W., Kuhn C., Moll R. & Gould V. E. Synaptophysin: a marker protein for neuroendocrine cells and neoplasms. Proc. Natl. Acad. Sci. USA 83, 3500–3504 (1986). 3010302 10.1073/pnas.83.10.3500 PMC323544

[b80] Nobels F. R. E., Kwekkeboom D. J., Bouillon R. & Lamberts S. W. J. Chromogranin A: Its clinical value as marker of neuroendocrine rumours. Eur. J. Clin. Invest. 28, 431–440 (1998). 9693933 10.1046/j.1365-2362.1998.00305.x

[b81] Lan M. S., Russell E. K., Lu J., Johnson B. E. & Notkins a L. IA-1, a new marker for neuroendocrine differentiation in human lung cancer cell lines. Cancer Res. 53, 4169–71 (1993). 8364910

[b82] Lan M. S. & Breslin M. B. Structure, expression, and biological function of INSM1 transcription factor in neuroendocrine differentiation. FASEB J. 23, 2024–2033 (2009). 19246490 10.1096/fj.08-125971 PMC2704596

[b83] Huang D. W., Lempicki R. a. & Sherman B. T. Systematic and integrative analysis of large gene lists using DAVID bioinformatics resources. Nat. Protoc. 4, 44–57 (2009). 19131956 10.1038/nprot.2008.211

[b84] Huang D. W., Sherman B. T. & Lempicki R. A. Bioinformatics enrichment tools: Paths toward the comprehensive functional analysis of large gene lists. Nucleic Acids Res. 37, 1–13 (2009). 19033363 10.1093/nar/gkn923 PMC2615629

[b85] Knights A. J., Funnell A. P. W., Crossley M. & Pearson R. C. M. Holding Tight: Cell Junctions and Cancer Spread. Trends Cancer Res. 8, 61–69 (2012). 23450077 PMC3582402

[b86] Leth-Larsen R., Lund R. R. & Ditzel H. J. Plasma membrane proteomics and its application in clinical cancer biomarker discovery. Mol. Cell. Proteomics 9, 1369–82 (2010). 20382631 10.1074/mcp.R900006-MCP200 PMC2938092

[b87] Sethi I. . A global analysis of the complex landscape of isoforms and regulatory networks of p63 in human cells and tissues. BMC Genomics 16, 584 (2015). 26251276 10.1186/s12864-015-1793-9 PMC4528692

[b88] Barton C. E. . Novel p63 target genes involved in paracrine signaling and keratinocyte differentiation. Cell Death Dis. 1, e74 (2010). 21151771 10.1038/cddis.2010.49 PMC3000738

[b89] Pallier K. . DeltaN TP63 reactivation, epithelial phenotype maintenance, and survival in lung squamous cell carcinoma. Tumor Biol. 33, 41–51 (2012). 10.1007/s13277-011-0239-5 21986963

[b90] Yan W., Wistuba I. I., Emmert-Buck M. R. & Erickson H. S. Squamous Cell Carcinoma - Similarities and Differences among Anatomical Sites. Am. J. Cancer Res. 1, 275–300 (2011). 21938273 PMC3175764

[b91] Barbieri C. E. & Pietenpol J. A. P63 and epithelial biology. Exp. Cell Res. 312, 695–706 (2006). 16406339 10.1016/j.yexcr.2005.11.028

[b92] Cheung W. K. C. . Control of alveolar differentiation by the lineage transcription factors GATA6 and HOPX inhibits lung adenocarcinoma metastasis. Cancer Cell 23, 725–738 (2013). 23707782 10.1016/j.ccr.2013.04.009 PMC3697763

[b93] Sulahian R. . An integrative analysis reveals functional targets of GATA6 transcriptional regulation in gastric cancer. Oncogene 33, 5637–48 (2014). 24317510 10.1038/onc.2013.517 PMC4050037

[b94] Park D. M. . Hes3 regulates cell number in cultures from glioblastoma multiforme with stem cell characteristics. Sci. Rep. 3, 1095 (2013). 23393614 10.1038/srep01095 PMC3566603

[b95] Otto O. . Real-time deformability cytometry: on-the-fly cell mechanical phenotyping. Nat. Methods 12, 199–202, 4 p following 202 (2015). 25643151 10.1038/nmeth.3281

[b96] Candès E. J., Li X., Ma Y. & Wright J. Robust principal component analysis? J. ACM 58, 11 (2011).

[b97] Szklarczyk D. . STRING v10: Protein-protein interaction networks, integrated over the tree of life. Nucleic Acids Res. 43, D447–D452 (2015). 25352553 10.1093/nar/gku1003 PMC4383874

[b98] Snel B., Lehmann G., Bork P. & Huynen M. A. STRING: a web-server to retrieve and display the repeatedly occurring neighbourhood of a gene. Nucleic Acids Res. 28, 3442–4 (2000). 10982861 10.1093/nar/28.18.3442 PMC110752

[b99] Meyer P. E., Lafitte F. & Bontempi G. minet: A R/Bioconductor package for inferring large transcriptional networks using mutual information. BMC Bioinformatics 9, 461 (2008). 18959772 10.1186/1471-2105-9-461 PMC2630331

[b100] Ammirati E. . Identification and predictive value of interleukin-6+ interleukin-10+ and interleukin-6-interleukin-10+ cytokine patterns in st-elevation acute myocardial infarction. Circ. Res. 111, 1336–1348 (2012). 22931953 10.1161/CIRCRESAHA.111.262477

[b101] Caporaso J. G. . QIIME allows analysis of high-throughput community sequencing data. Nat. Methods 7, 335–6 (2010). 20383131 10.1038/nmeth.f.303 PMC3156573

[b102] Wang Q., Garrity G. M., Tiedje J. M. & Cole J. R. Naïve Bayesian classifier for rapid assignment of rRNA sequences into the new bacterial taxonomy. Appl. Environ. Microbiol. 73, 5261–5267 (2007). 17586664 10.1128/AEM.00062-07 PMC1950982

[b103] Caporaso J. G. . PyNAST: A flexible tool for aligning sequences to a template alignment. Bioinformatics 26, 266–267 (2010). 19914921 10.1093/bioinformatics/btp636 PMC2804299

[b104] Haas B. J. . Chimeric 16S rRNA sequence formation and detection in Sanger and 454-pyrosequenced PCR amplicons. Genome Res. 21, 494–504 (2011). 21212162 10.1101/gr.112730.110 PMC3044863

[b105] Ravasi T. . An Atlas of Combinatorial Transcriptional Regulation in Mouse and Man. Cell 140, 744–752 (2010). 20211142 10.1016/j.cell.2010.01.044 PMC2836267

[b106] Donders A. R. T., van der Heijden G. J. M. G., Stijnen T. & Moons K. G. M. Review: A gentle introduction to imputation of missing values. J. Clin. Epidemiol. 59, 1087–1091 (2006). 16980149 10.1016/j.jclinepi.2006.01.014

[b107] Jolliffe I. T. Principal Component Analysis. (Wiley Online Library, 2002).

[b108] Christmas Rowan, Avila-Campillo Iliana, Bolouri Hamid, Schwikowski Benno, Anderson Mark, Kelley Ryan, Landys Nerius, Workman Chris, Ideker Trey, Cerami Ethan, Sheridan Rob, Bader Gary D. & Sander C. Cytoscape: a software environment for integrated models of biomolecular interaction networks. Am. Assoc. Cancer Res. Educ. B. 13, 12–16 (2005).

[b109] Bell G. W. & Lewitter F. [22] Visualizing Networks. Methods Enzymol. 411, 408–421 (2006). 16939803 10.1016/S0076-6879(06)11022-8

[b110] Gilbert S. In Developmental Biology doi: 10.1016/j.ydbio.2007.08.033 (2003).

[b111] Ben Pansky Review of Medical Embryology (1982).

[b112] Dudek R. W. High-yield histology. (Williams & Wilkins, 2000).

[b113] Coalson R. E. & Tomasek J. J. In Embryology 1–8 (Springer, 1992).

[b114] Oliveros J. C. V. E. N. N. Y. An interactive tool for comparing lists with Venn Diagrams. BioinfoGP of CNB-CSIC at http://bioinfogp.cnnb.csic.es/tools/venny/index.html (2007).

