# Supplementary material for: Enlightening discriminative network functional modules behind Principal Component Analysis separation in differential-omic science studies
Source: Sci Rep. 2017 Mar 13;7:43946. doi: 10.1038/srep43946 (PMC5347127; doi:10.1038/srep43946)
Supplement: Supplementary Information [file srep43946-s1.docx]

## **Enlightening discriminative network functional modules behind Principal Component Analysis separation in differential-omic science studies.**

Sara Ciucci^1,2,†^, Yan Ge^1,†^, Claudio Durán^1,†^, Alessandra Palladini^1,2,3,†^, Víctor Jiménez-Jiménez^4^, Luisa María Martínez-Sánchez^1^, Yuting Wang^5,6^, Susanne Sales^5^, Andrej Shevchenko^5^, Steven W. Poser^7^ , Maik Herbig^8^,  Oliver Otto^8^,  Andreas Androutsellis-Theotokis^6,7,9^, Jochen Guck^8^, Mathias J. Gerl^2^ and Carlo Vittorio Cannistraci ^1,*^

^1^Biomedical Cybernetics Group, Biotechnology Center (BIOTEC), Center for Molecular and Cellular Bioengineering (CMCB), Department of Physics, Technische Universität Dresden, Tatzberg 47/49, 01307 Dresden, Germany

^2^Lipotype GmbH, Tatzberg 47, 01307 Dresden, Germany

^3^Membrane Biochemistry Group, DZD Paul Langerhans Institute, Technische Universität Dresden, Tatzberg 47/49, 01307 Dresden, Germany

^4^Integrin Signalling Group, Fundación Centro Nacional de Investigaciones Cardiovasculares Carlos III, Melchor Fernández Almagro 3, 28029 Madrid, Spain

^5^MPI of Molecular Cell Biology and Genetics, Pfotenhauerstrstraße 108, 01307 Dresden, Germany

^6^Center for Regenerative Therapies Dresden (CRTD), Center for Molecular and Cellular Bioengineering (CMCB), Technische Universität Dresden, Fetscherstraße 105, 01307 Dresden, Germany

^7^Department of Internal Medicine III, University Hospital Carl Gustav Carus at the Technische Universität Dresden, Fetscherstr.74, 01307 Dresden, Germany

^8^Cellular Machines Group, Biotechnology Center (BIOTEC), Center for Molecular and Cellular Bioengineering (CMCB), Technische Universität Dresden, Tatzberg 47/49, 01307 Dresden, Germany

^9^Department of Stem Cell Biology, Centre for Biomolecular Sciences, Division of Cancer and Stem Cells, School of Medicine, University of Nottingham, Nottingham NG7 2RD, U.K.

*Correspondence should be addressed to: [kalokagathos.agon@gmail.com](mailto:kalokagathos.agon@gmail.com)

†The first four authors should be regarded as joint First Authors.

**Supplementary information**

| Discriminative correlation network based on PC-corr formula |
| --- |
| Input:  Data matrix, with samples on the rows and features on the columns;  Feature Names, for example, gene names, lipid names;  Sample labels, identifying to which group the samples belong;  Sample names;  Output:  PCA results file with respective evaluators in Excel format;  Network file and Node property file in Excel format;  Description:   1. Ask the user the type of labels that represent the data: ranked labels (either discrete or continuous) or class labels; 2. Remove from the dataset features that have the same identical value across all the samples; 3. Normalization of the obtained dataset considering 11 different kinds of normalizations (the list is given in the below section) plus the original non-normalized dataset; 4. List of PCA options: either centred or not centred, pre-processed by the different normalizations or non-normalized; 5. Quantitative evaluation of PCA discrimination along each dimension by different *evaluators*: p-value, AUC, AUPR, correlation (see the PC-corr user guide for details); 6. User choice of the *evaluator*, with respect to which the list of PCA options will be ordered; 7. Ordered PCA options’ results returned on the screen and as an excel table; 8. User selection of the dataset normalization, centred or non-centred PCA, selected discriminative dimension and the cut-off (or multiple cut-offs) for PC-corr network. 9. Plot of the selected PCA in the 2D space, where the first dimension is the chosen one; 10. Generation of the Figure associated to the PCA plot: top panel reports the selected evaluator for each principal components; bottom panel reports the explained variance accounted by each dimension; 11. Get the PCA loadings (*V*) of the chosen dimension; 12. Heuristic function: $V^{*}\left( i \right)=sign\left( V\left( i \right) \right)*{log}_{10}\left( 1+\frac{\left\vert V\left( i \right) \right\vert}{mean\left( \left\vert\boldsymbol{V} \right\vert\right)} \right)$ ; 13. Scaling: $V^{new}\left( i \right)=sign \left( V^{*}\left( i \right) \right)*\frac{\left\vert V^{*}\left( i \right) \right\vert-\min\left( \boldsymbol{x} \right)}{\max\left( \boldsymbol{x} \right)-\min\left( \boldsymbol{x} \right)}$ where $\boldsymbol{x}\mathbf{=}\left\vert\boldsymbol{V}^{\mathbf{*}} \right\vert\mathbf{;}$   {Note: After scaling, the $V^{new}(i)$ will range from -1 to 1}   1. Pearson correlation coefficients ${-1<c}_{i,j}<1$ on all the features; 2. Calculate the PC-corr: ${PC\_corr}_{i,j}=sign(c_{i,j})*min(\vert V^{new}(i)\vert,\vert V^{new}(j)\vert, \vert c_{i,j}\vert)$ ; 3. Apply the selected cut-off between 0 and 1:${\vert PCcorr}_{i,j}\vert>cutoff$ ;   Example: ${\vert PC\_corr}_{i,j}\vert>0.6$;  {Note: the cut-off is selected by the operator in relation to his/her needs to explore the network structure and organization at different levels}   1. Remove the single nodes (without any interaction) and get the network table and node colour table; 2. Visualisation of the PC-corr network; 3. If more than one cut-off were chosen, repeat steps from 12 to 18 for every cut-off. 4. Return Table in Excel format with separate sheets: network table and node colour table.   For more than one cut-off, network and node colour table for each cut-off are present in separate sheets. |

**Table S1.** Description of the algorithm for the construction of the PC-corr network from a general omic dataset. Legend: $\boldsymbol{V}$ vector of all loadings with the i-th element, $V\left( i \right) corresponding to the i-th feature$; $V^{*}\left( i \right)$ normalized loading of the i-th feature with the heuristic function; $V^{new}(i)$ normalized and scaled loading of the i-th feature; $\left| \cdot\right|$: absolute value or modulus; $sign(\cdot)$**:** sign function (signum function); $c_{i,j}$: Pearson correlation coefficient of features $i$ and $j$; $min(\cdot)$**:** minimum operator; ${PC\_corr}_{i,j}$: PC-corr edge value for nodes $i$ and $j$.

**List of Normalizations:**

1. DSOR: dividing by the sum over the samples;
2. DSOC: dividing by the sum over the features;
3. LOG: logarithm with base 10 of each data element plus 1 (to avoid problems with 0 values). In case the data have negative values, remember to scale the minimum data value to 0 before to perform this normalization.
4. ZSCORE: z-score for each data element such that the features are centred to have mean 0 and scaled to have standard deviation 1;
5. QUANTILE T: quantile normalization over the samples;
6. QUANTILE: quantile normalization over the features;
7. ZSCORE T: z-score for each data element such that the samples are centred to have mean 0 and scaled to have standard deviation 1;
8. PLUS(ABS(MIN)): adding to each data element the minimum present in the data matrix, in absolute value.
9. PARETO SCALING: each feature is centred to have mean 0 and scaled by the square root of the standard deviation of the feature's values;
10. SQRT: square root of each data element;
11. MANORM: scaling the values in each feature, dividing by the mean of the feature.


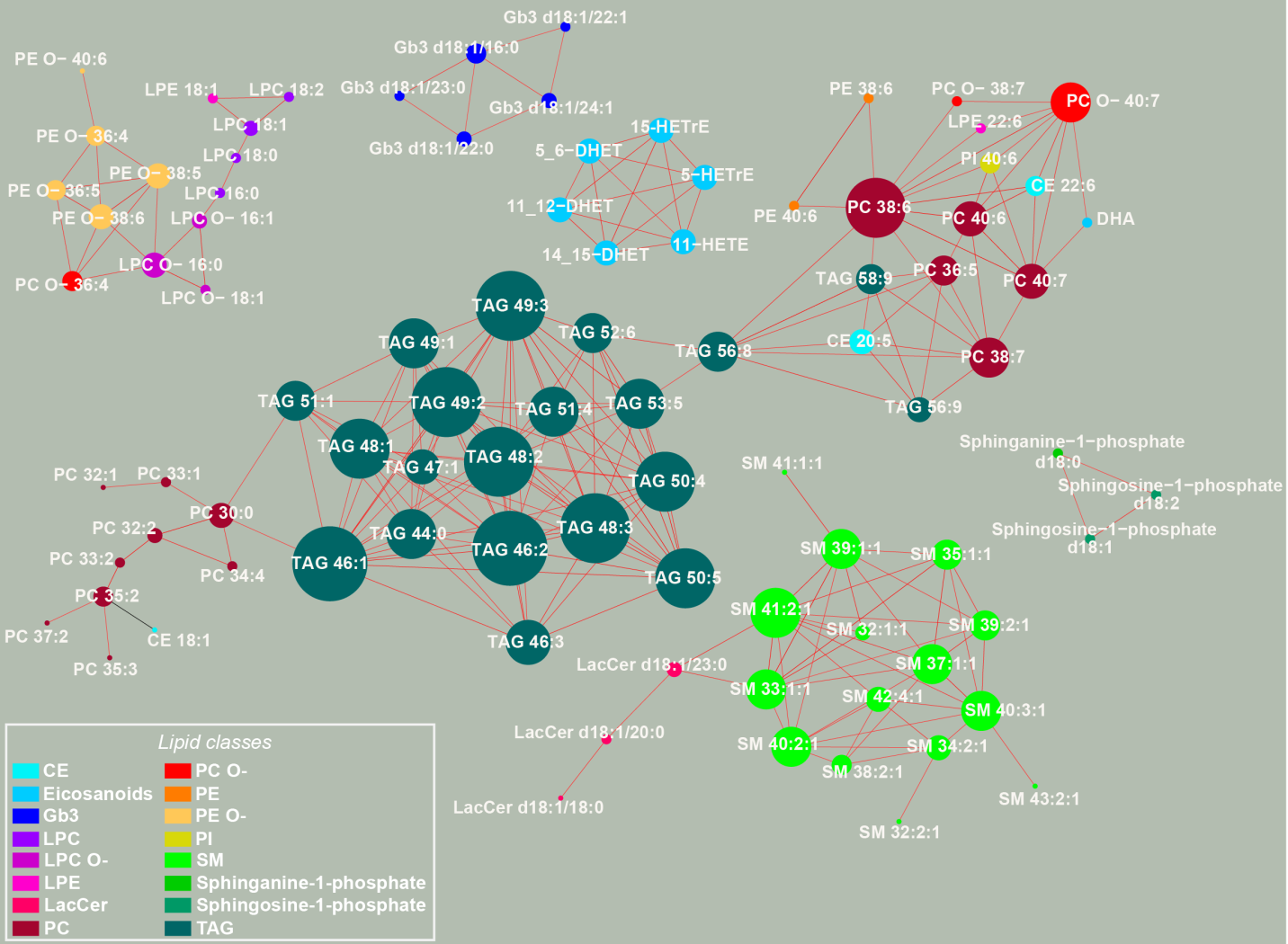


Figure S1. PC-corr network of the lipidomic dataset (cut-off 0.6) where each node colour corresponds to a lipid class.


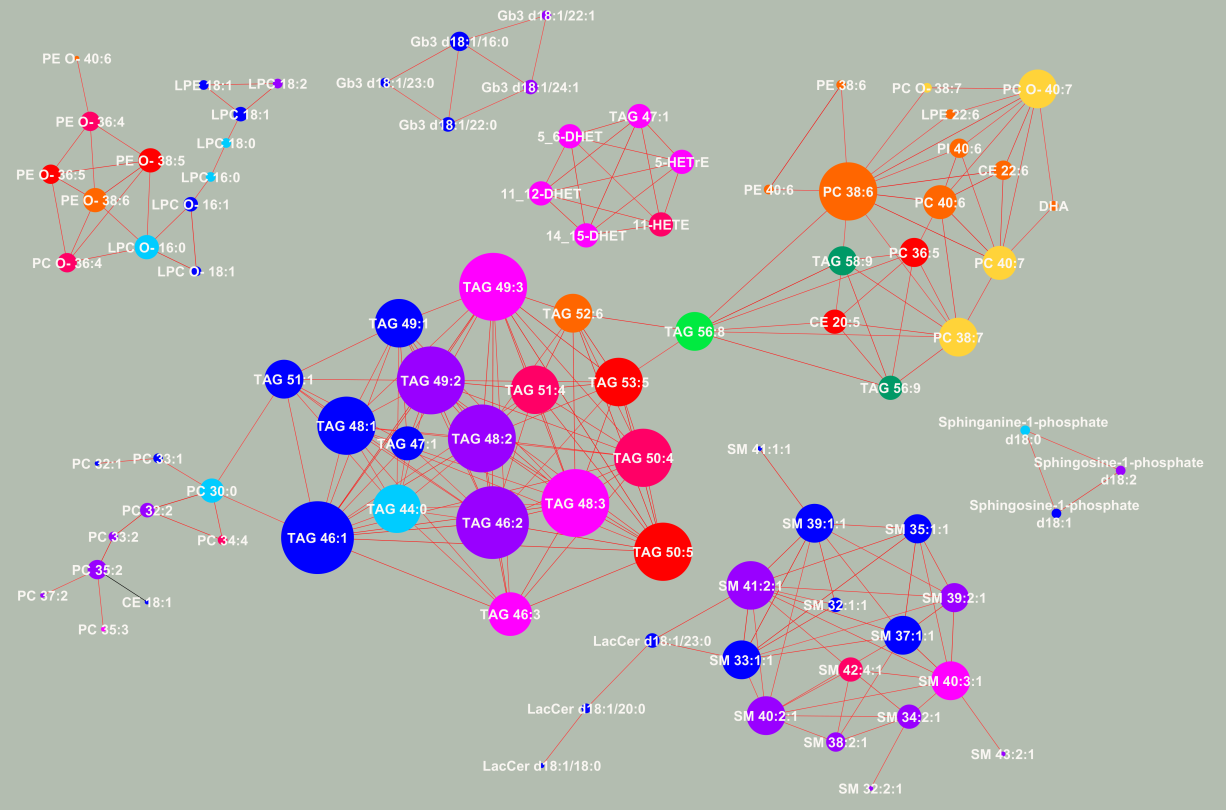

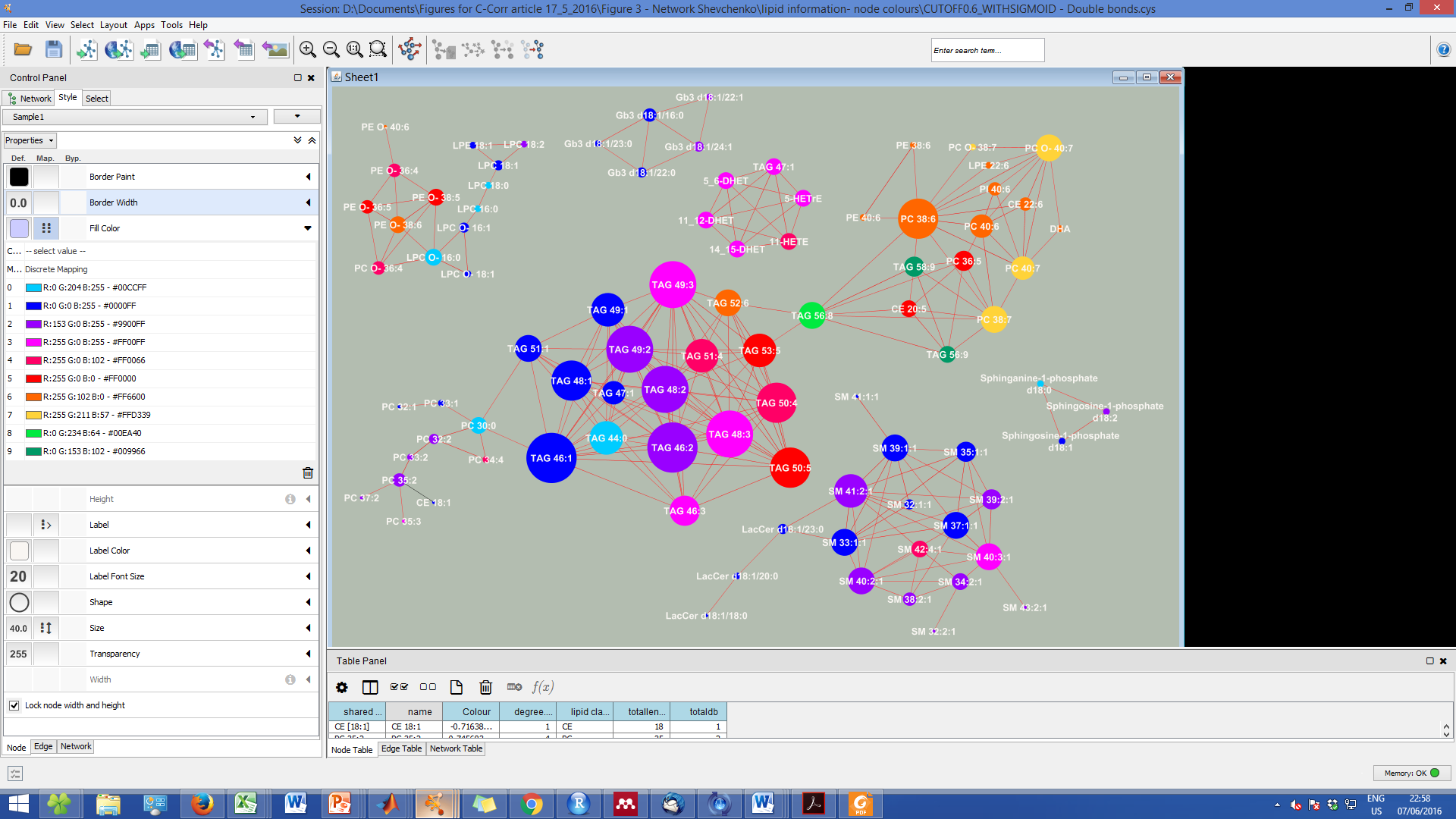


Figure S2. PC-corr network of the lipidomic dataset (cut-off 0.6) where each node colour corresponds to the number of double bonds in its hydrocarbon moieties.


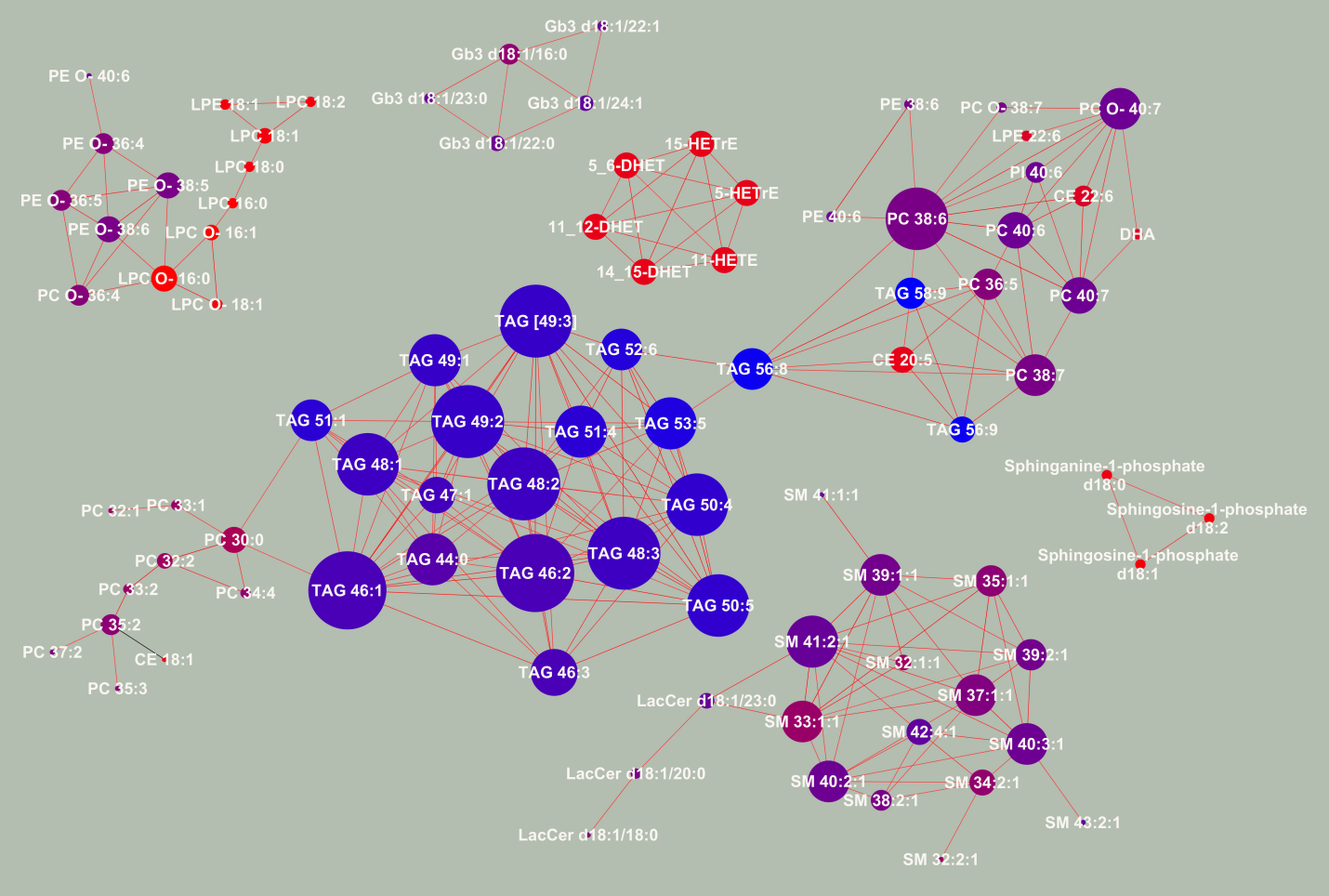

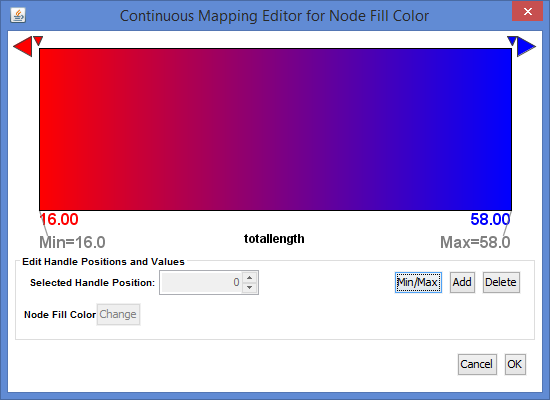


Figure S3. PC-corr network of the lipidomic dataset (cut-off 0.6) where each node colour corresponds to the number of carbon atoms in the fatty acid chains.


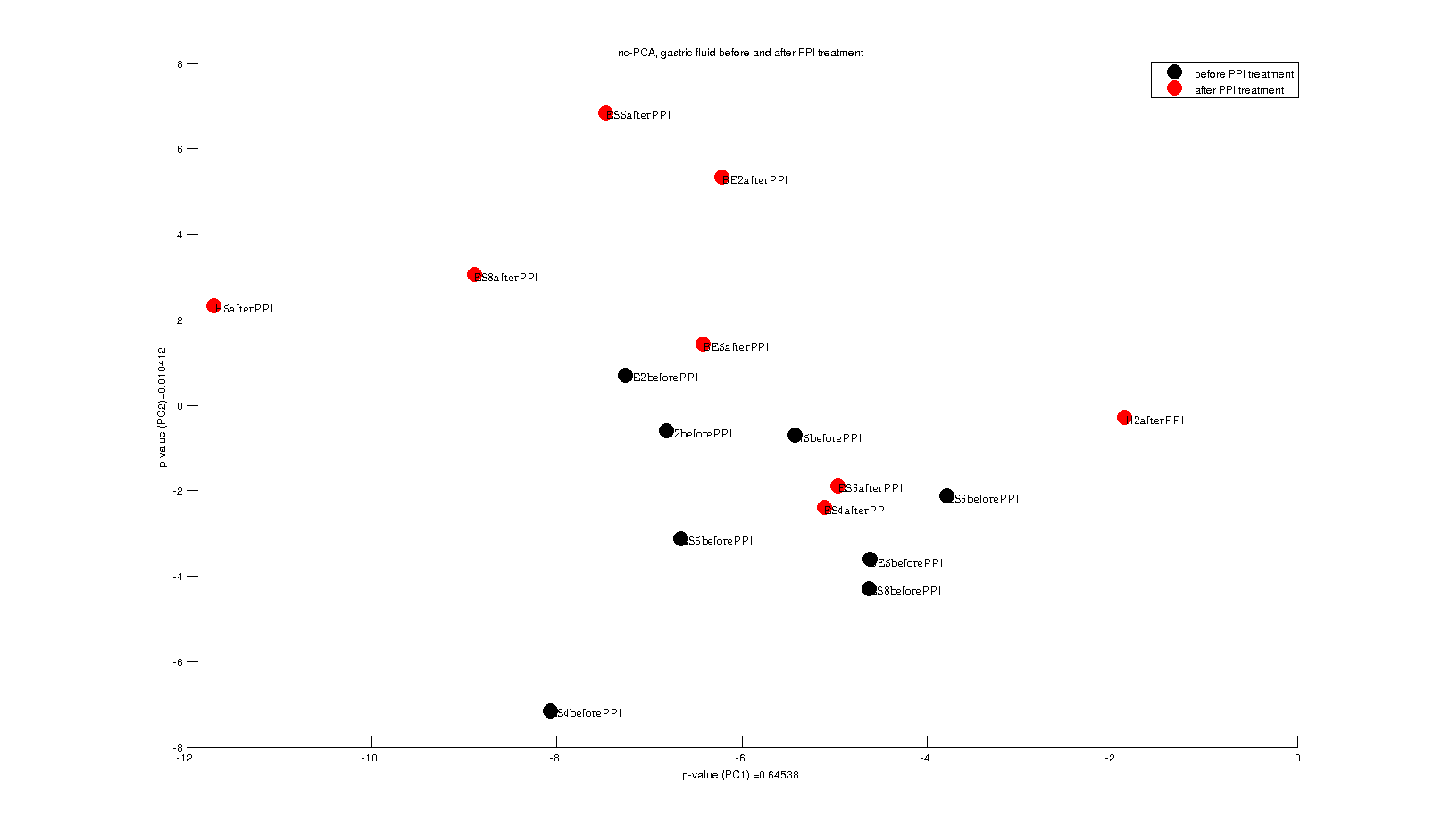


b

a


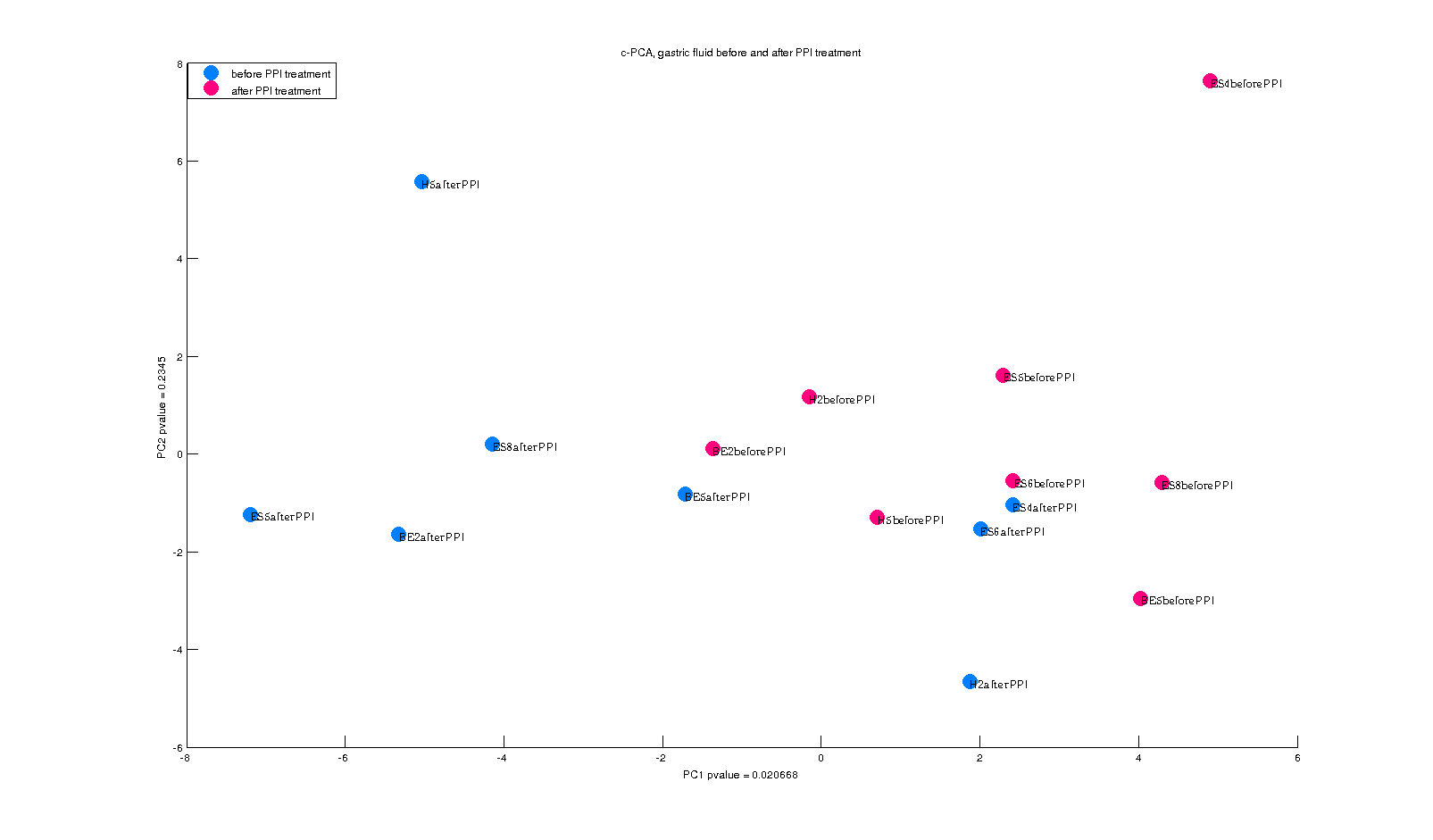


Figure S4. PCA plots of the metagenomic dataset. PCA was performed in its non-centered (a) and centered (b) versions on the gastric fluid metagenomes. (a) ncPCA offers a better discrimination (Mann-Whitney test p-value of PC2= 0.01) of the samples before (black dots) and after (red dots) PPI treatment than cPCA, and was therefore used to build the PC-corr network. (b) cPCA discriminates samples before (blue dots) and after (pink dots) PPI treatment along PC1, and with a higher Mann-Whitney test p-value (0.02) than ncPCA’s.


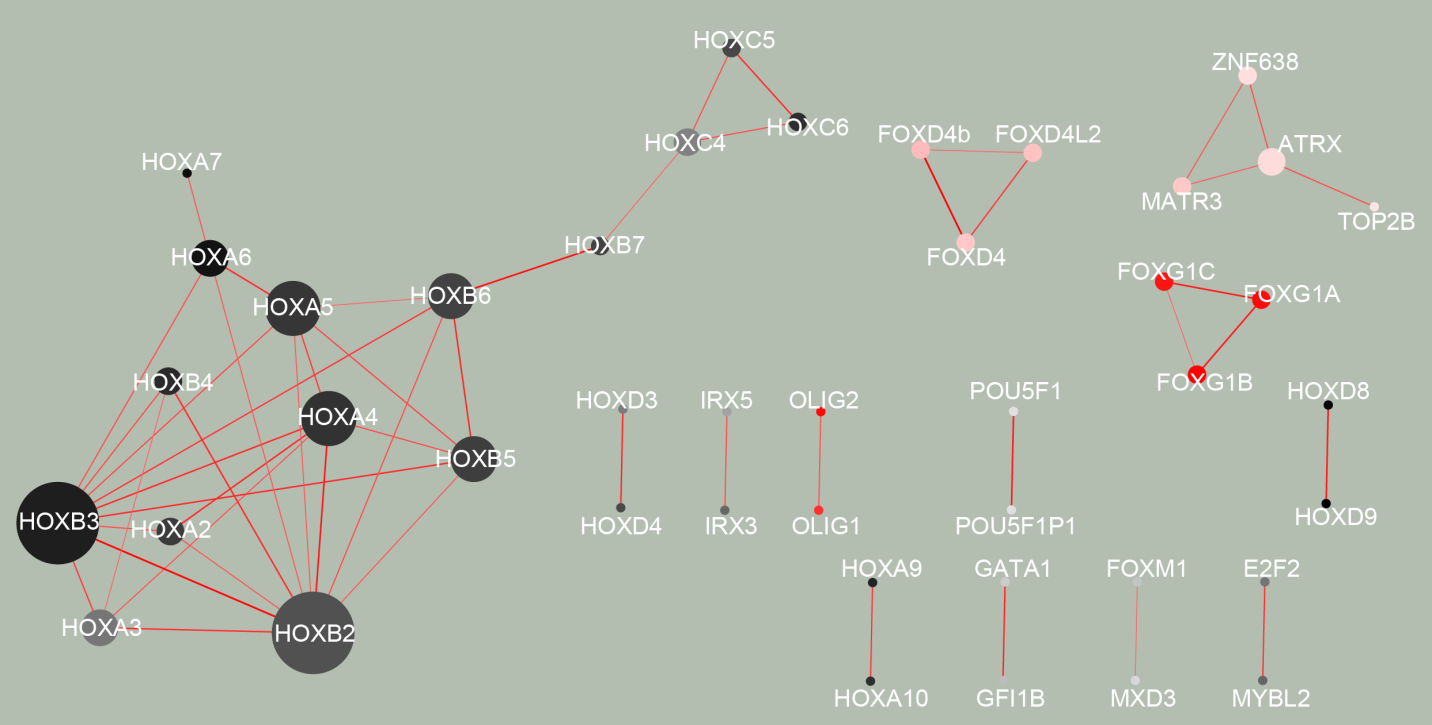


Figure S5. P-value network of the developmental genomic dataset at cut-off = 0.927. For the developmental genomic dataset, the P-value network with approximately the same number of nodes of the PC-corr (Fig. 5c) was constructed (cut-off=0.927).


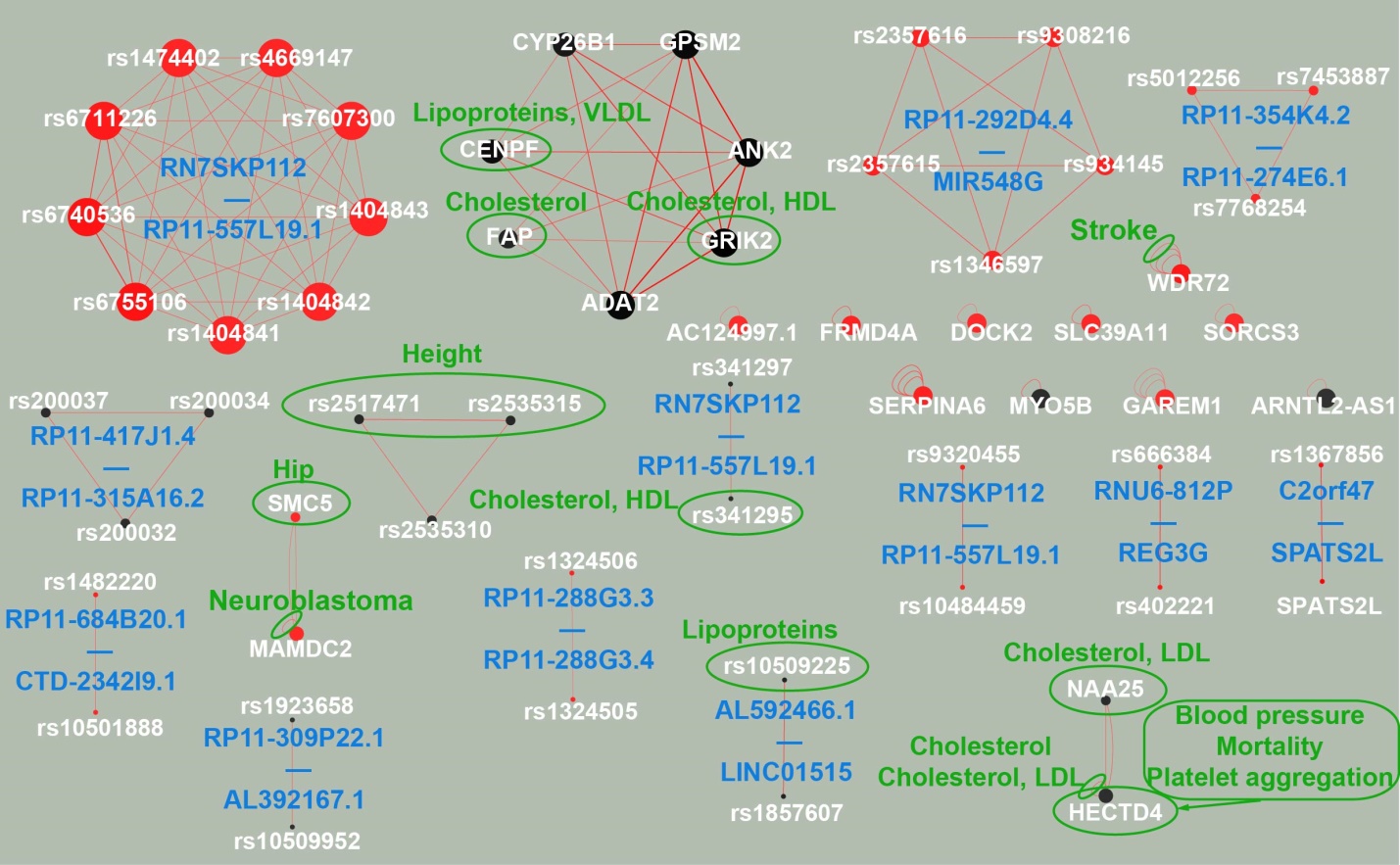


**Figure S6. PC-corr** **network of the population genetic dataset.** As fig. 6c, it shows the SNP PC-corr network at 0.8 cut-off, where the SNP name (rs#, in white colour) is changed to the name of the gene that contains the mutation when it is an intron, missense or 3’ UTR variant. When this is not the case, the SNP name is left unchanged and, additionally, it is reported (in blue colour) the region between two genes where the SNP lies. Moreover, as in fig. 6c, the green circles highlight phenotype-associated SNPs, including disease related variants.


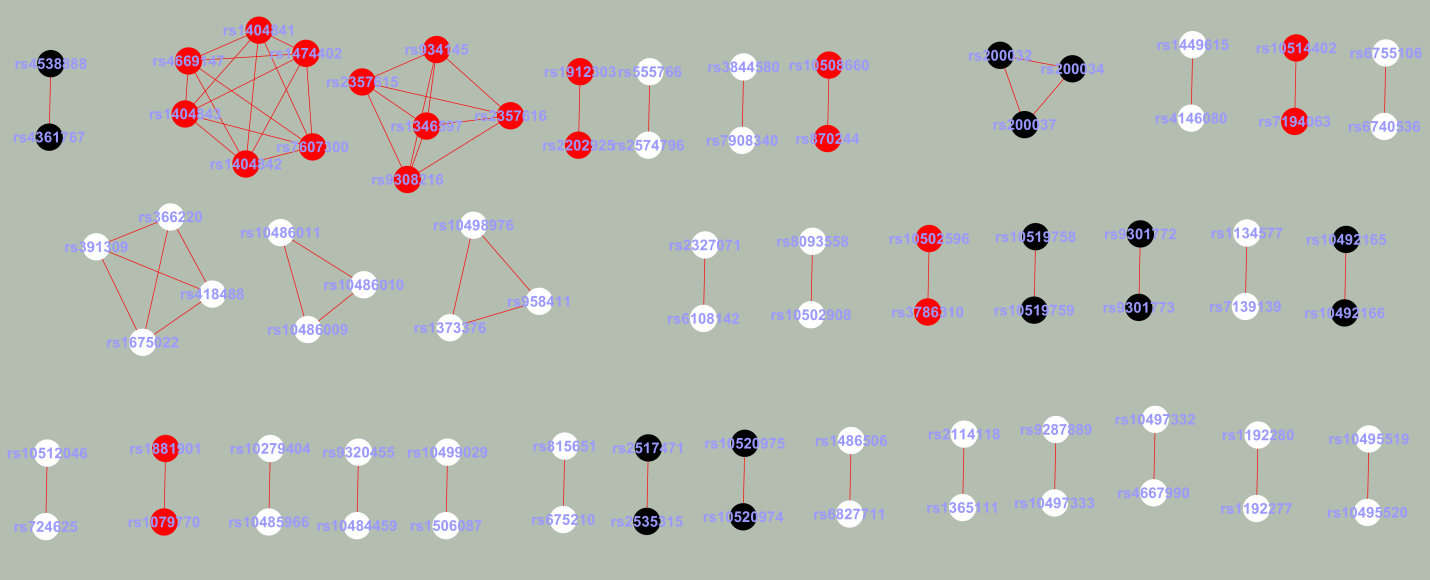


Figure S7. P-value network of the population genetic dataset at cut-off = 0.998. For the population genetic dataset, the P-value network with approximately the same number of nodes of the PC-corr (Fig. 6c) was constructed (cut-off=0.998).


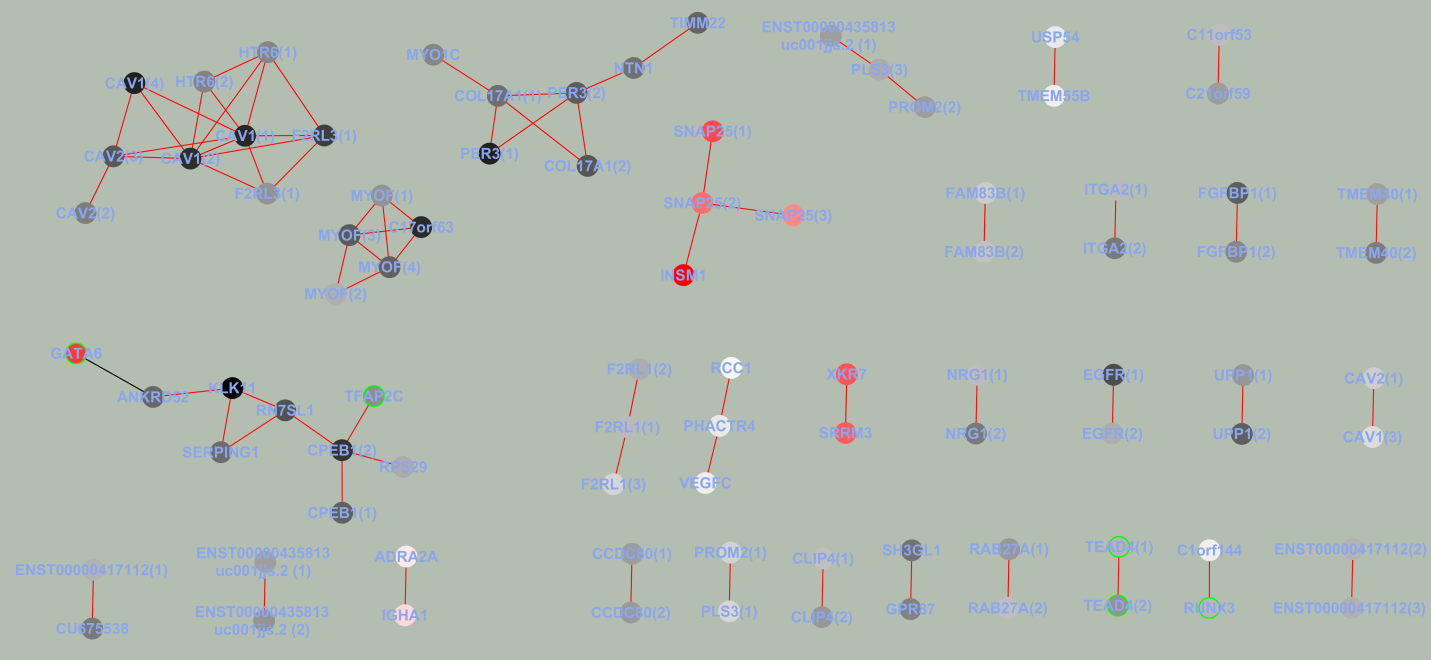


Figure S8. P-value network of the carcinoma cell-wide promoteromic dataset at cut-off = 0.973. For the carcinoma cell-wide promoteromic dataset, the P-value network with approximately the same number of nodes of the PC-corr (Fig. 7c) was constructed (cut-off=0.973).


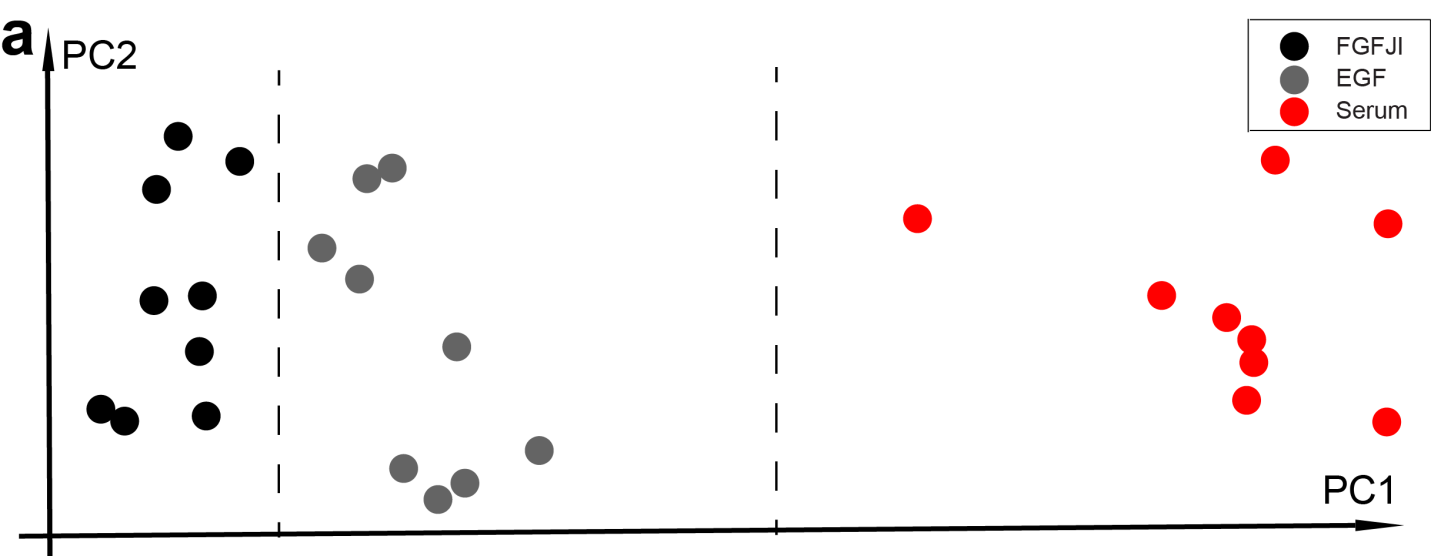


Figure S9. PCA of the cancer stem cell mechanomic dataset. Three different colours represent the same cells cultured in three different conditions.


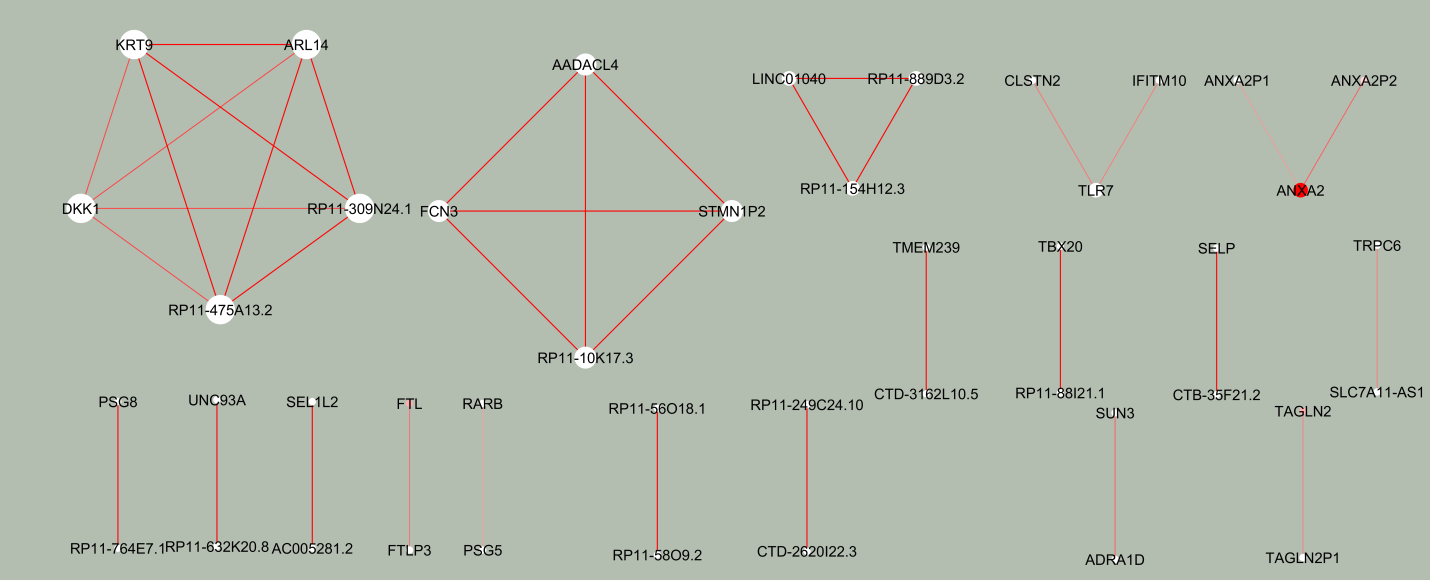


Figure S10. P-value network of the cancer stem cell mechanomic dataset at cut-off = 0.996. For the cancer stem cell mechanomic dataset, the P-value network with approximately the same number of nodes of the PC-corr (Fig. 8a) was constructed (cut-off=0.996).

**Leave-one-out-cross-validation (LOOCV)**

**Lipidomics**

a

**
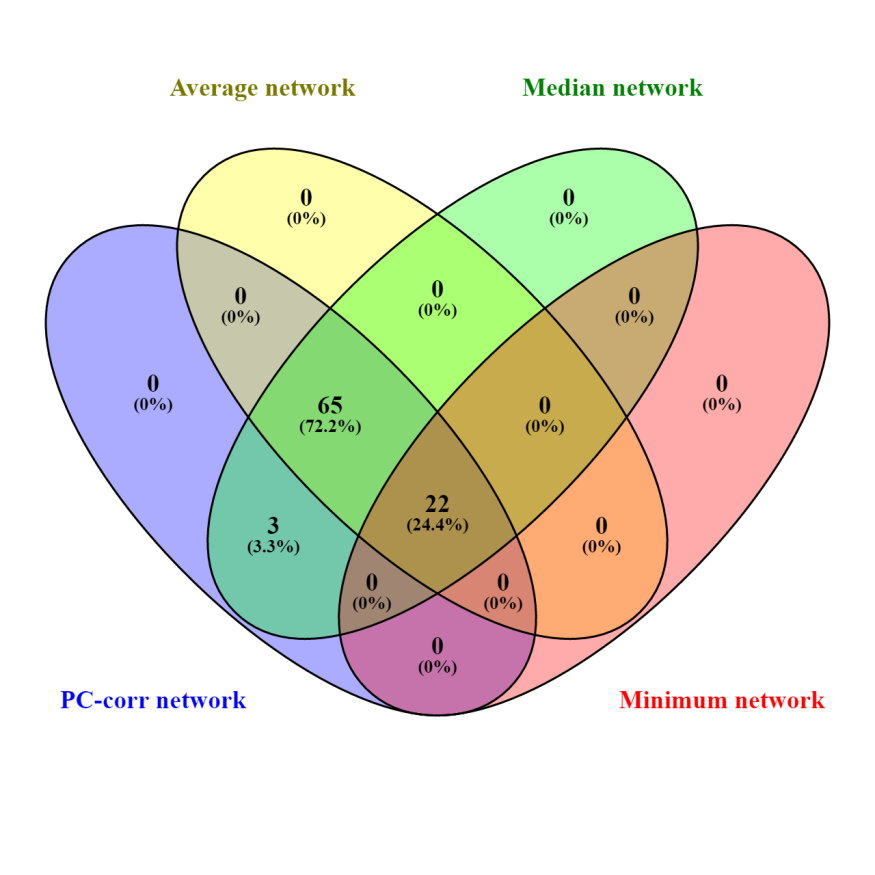
**

**Nodes**

**
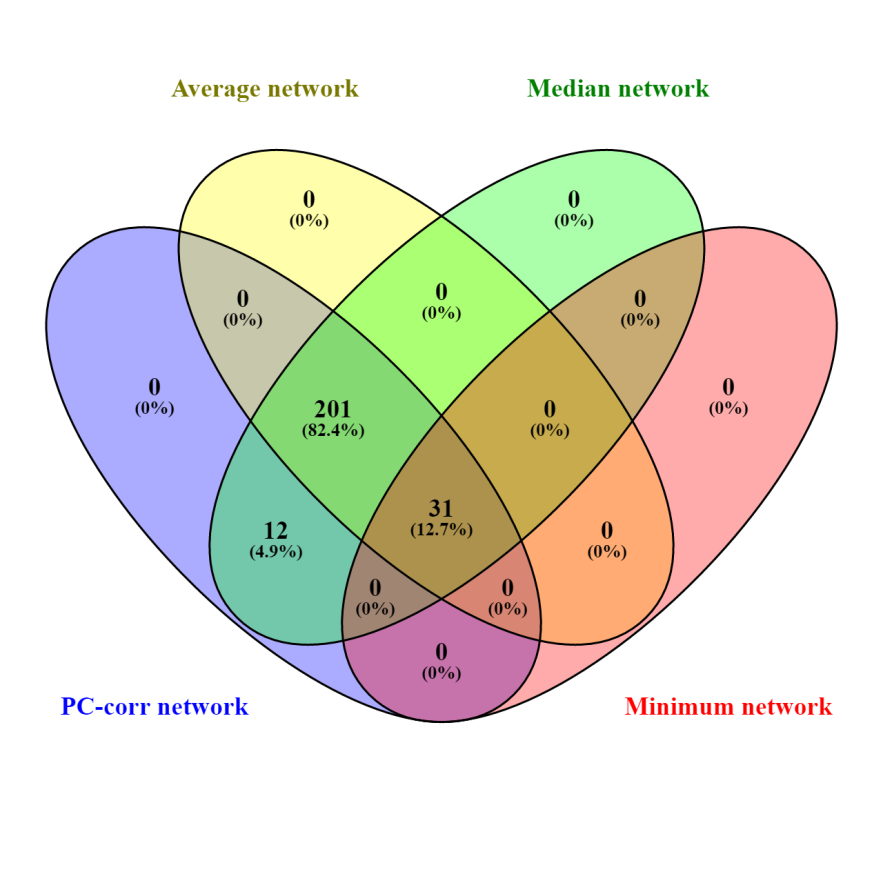
**

b

**Edges**

Figure S11. Comparison of the PC-corr network and the three networks obtained from LOOCV under cut-off 0.6. (a) Venn diagrams that show the node differences between the PC-corr network and the average, minimum and median networks obtained from LOOCV under cut-off 0.6. (b) Venn diagrams that show the edge differences between the PC-corr network and the average, minimum and median networks obtained from LOOCV under cut-off 0.6.

Metagenomics (cut-off 0.55)

**
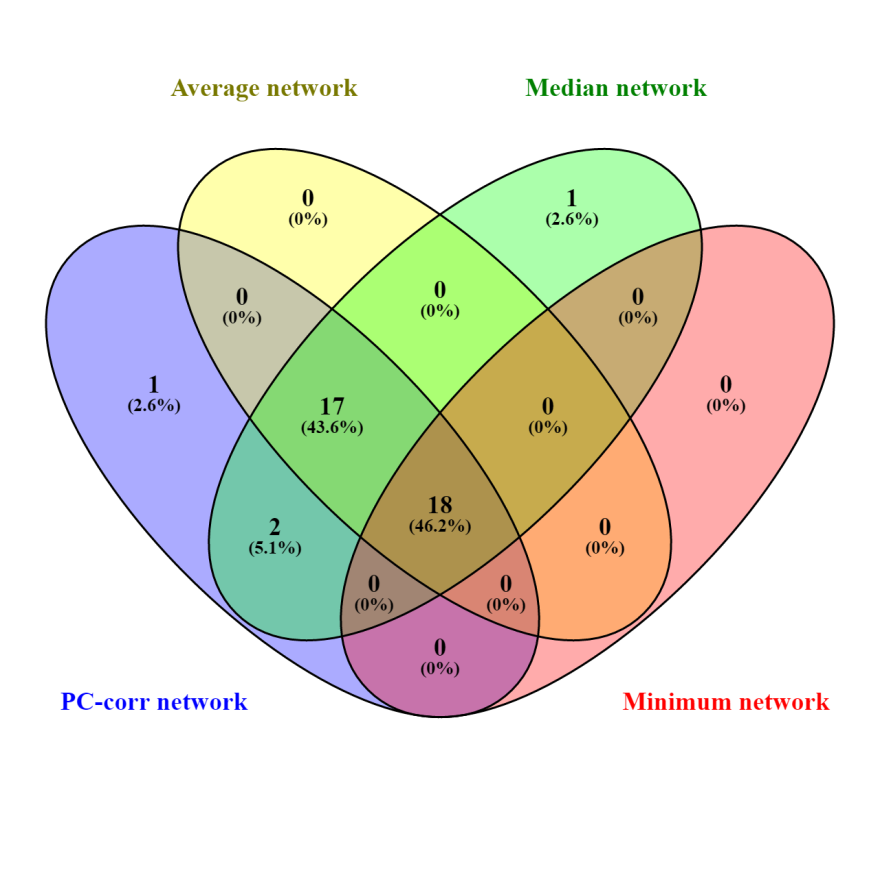
**

a

**Nodes**

**
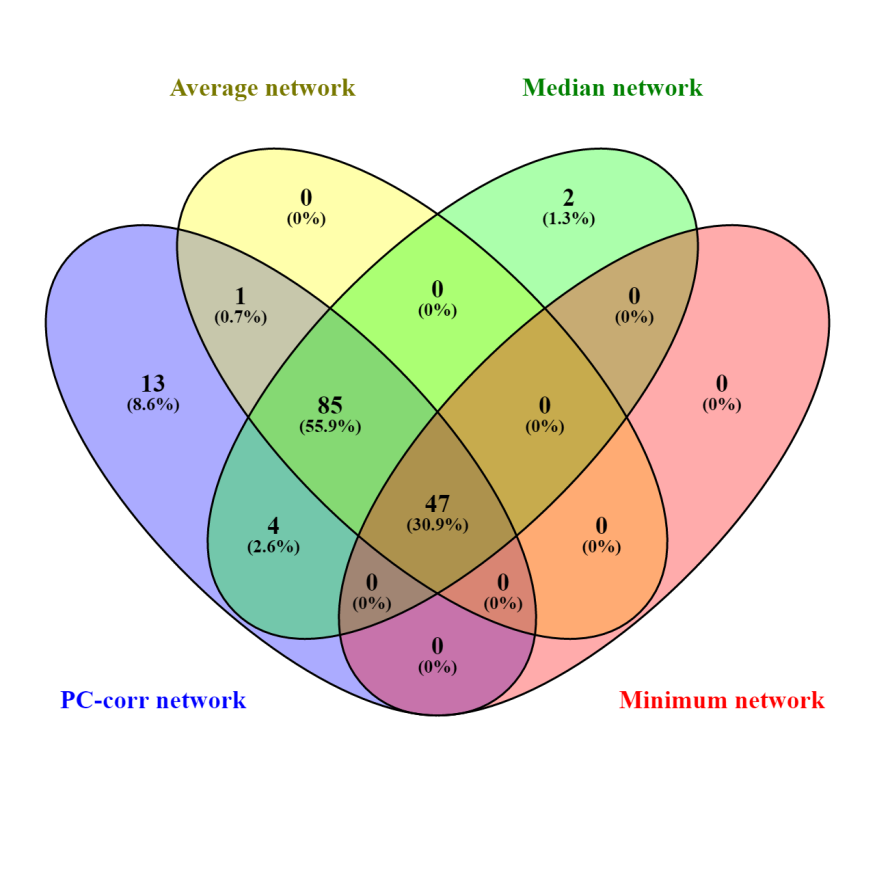
**

b

**Edges**

Figure S12. Comparison of the PC-corr network and the three networks obtained from LOOCV under cut-off 0.55. (a) Venn diagrams that show the node differences between the PC-corr network and the average, minimum and median networks obtained from LOOCV under cut-off 0.55. (b) Venn diagrams that show the edge differences between the PC-corr network and the average, minimum and median networks obtained from LOOCV under cut-off 0.55.

**Metagenomics (cut-off 0.6)**

**
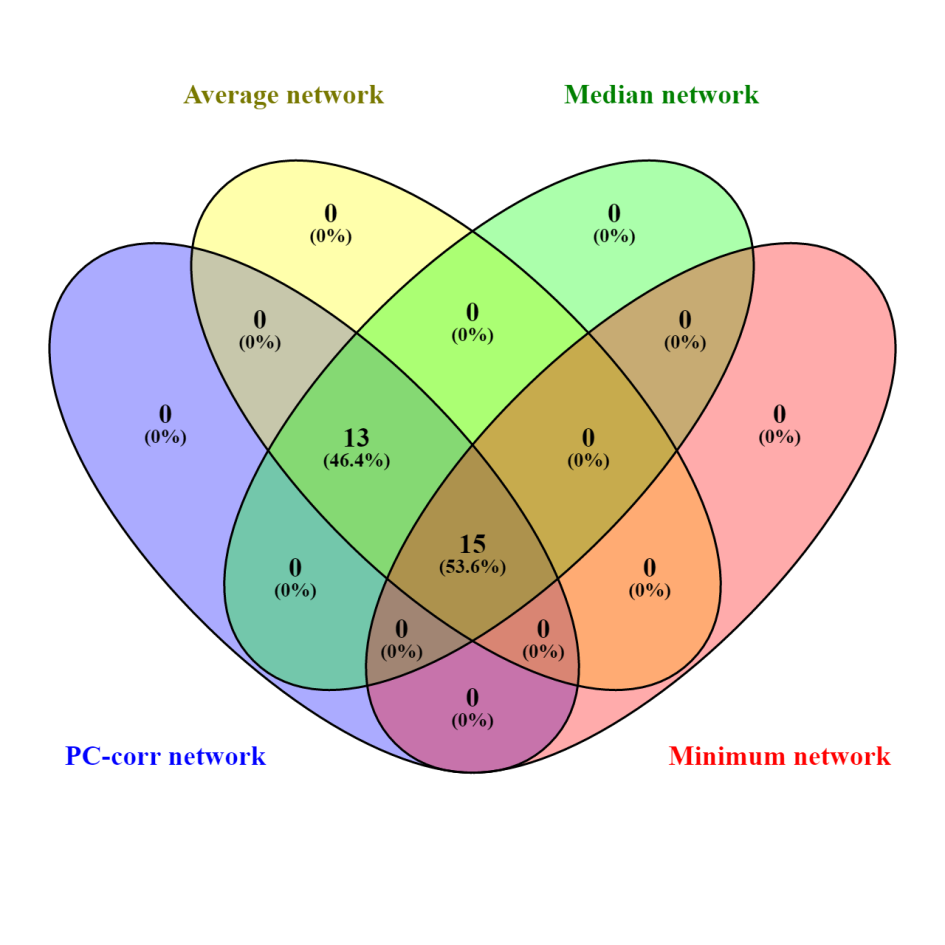
**

a

**Nodes**


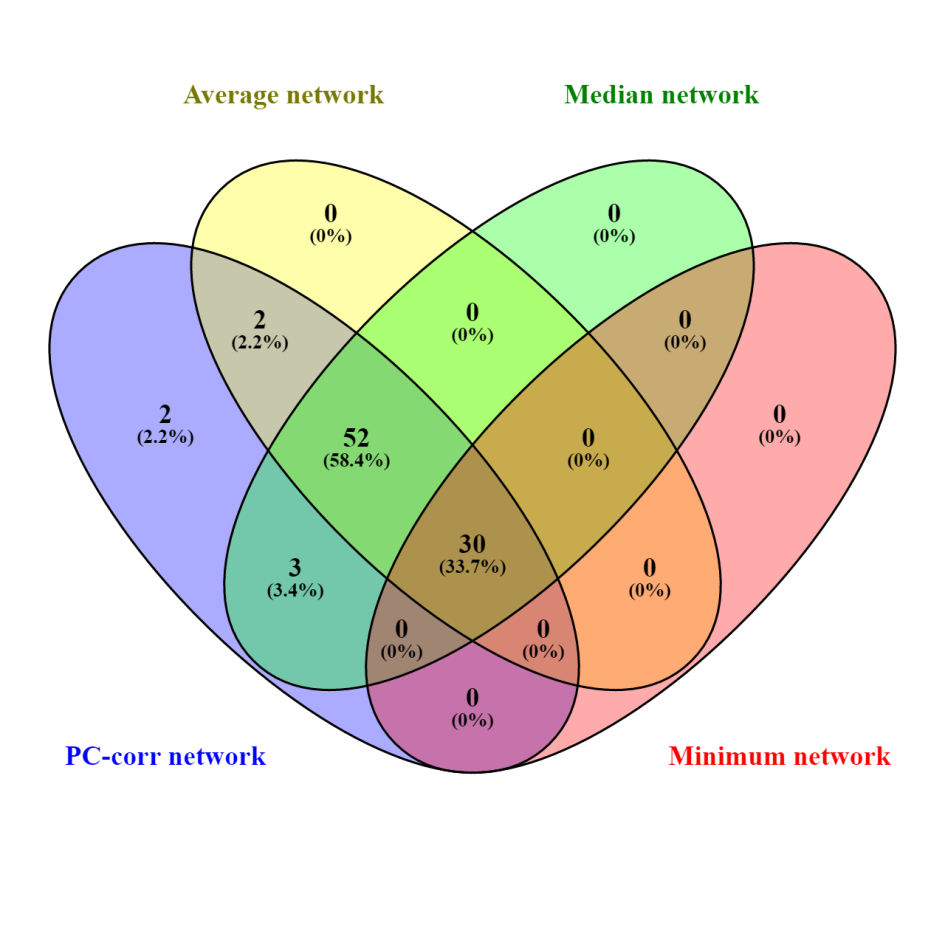


b

**Edges**

Figure S13. Comparison of the PC-corr network and the three networks obtained from LOOCV under cut-off 0.6. (a) Venn diagrams that show the node differences between the PC-corr network and the average, minimum and median networks obtained from LOOCV under cut-off 0.6. (b) Venn diagrams that show the edge differences between the PC-corr network and the average, minimum and median networks obtained from LOOCV under cut-off 0.6.

**Developmental genomics**

**
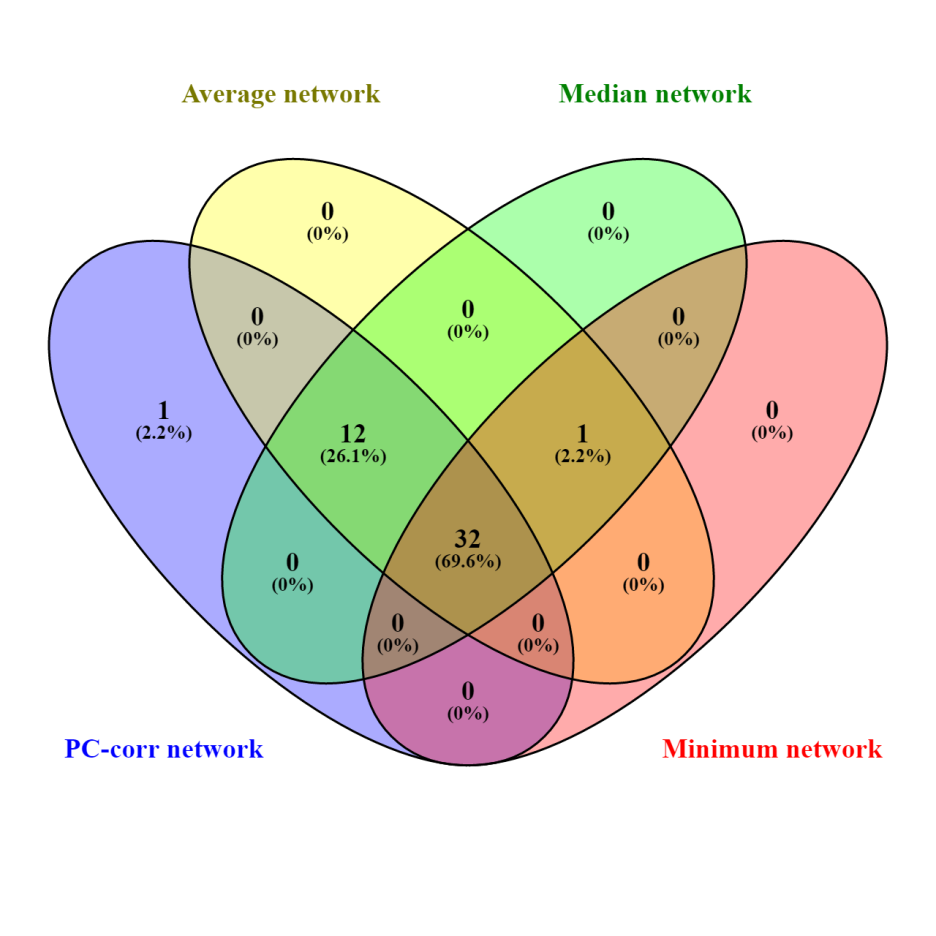
**

a

**Nodes**

**
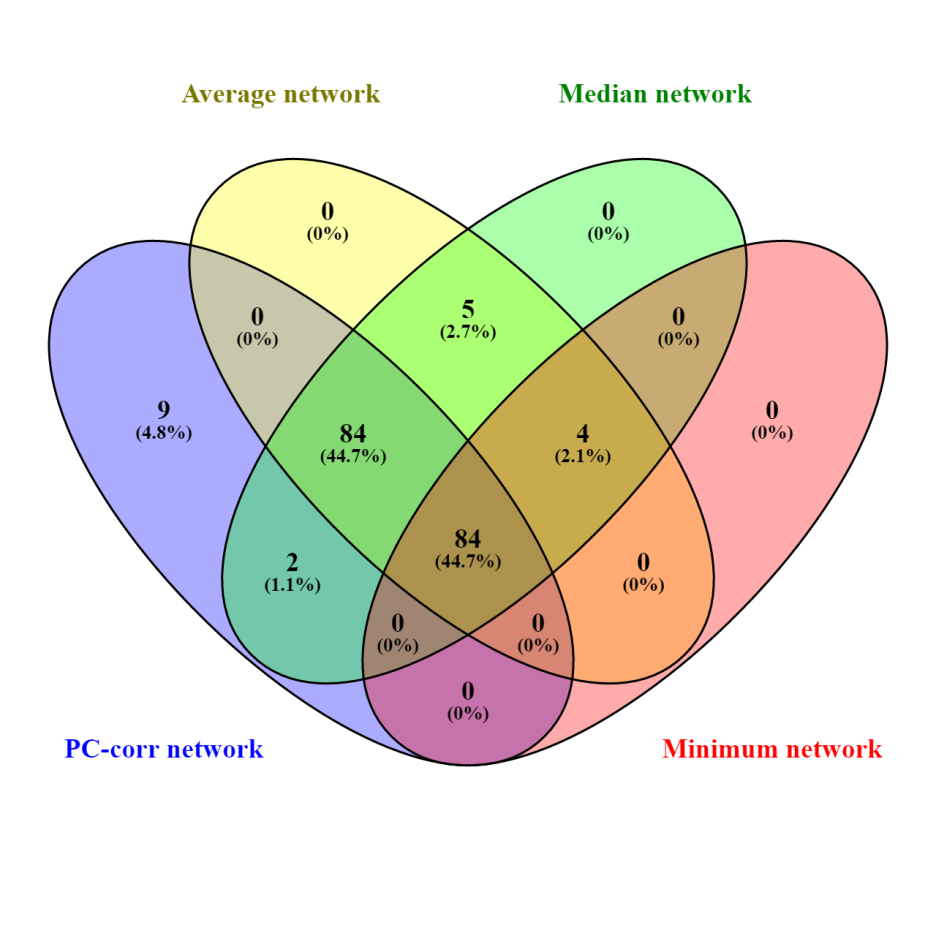
**

b

**Edges**

Figure S14. Comparison of the PC-corr network and the three networks obtained from LOOCV under cut-off 0.8. (a) Venn diagrams that show the node differences between the PC-corr network and the average, minimum and median networks obtained from LOOCV under cut-off 0.8. (b) Venn diagrams that show the edge differences between the PC-corr network and the average, minimum and median networks obtained from LOOCV under cut-off 0.8.

**Population genetics**

**
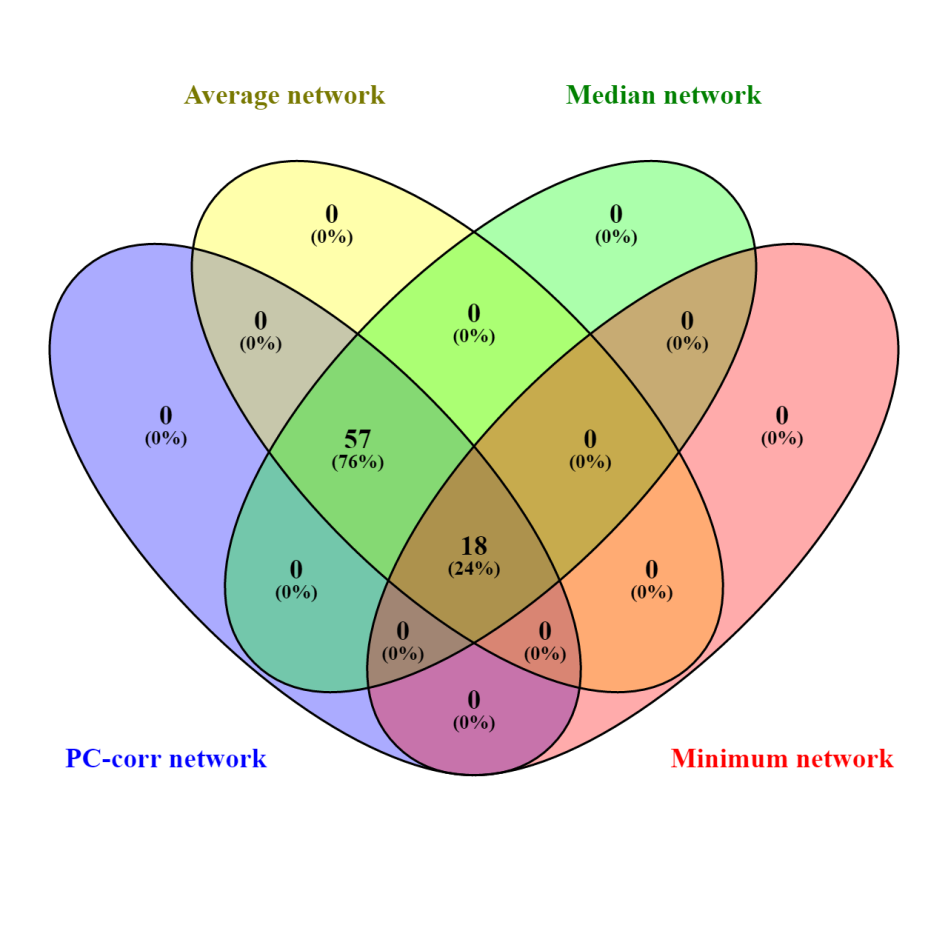
**

a

**Nodes**

**
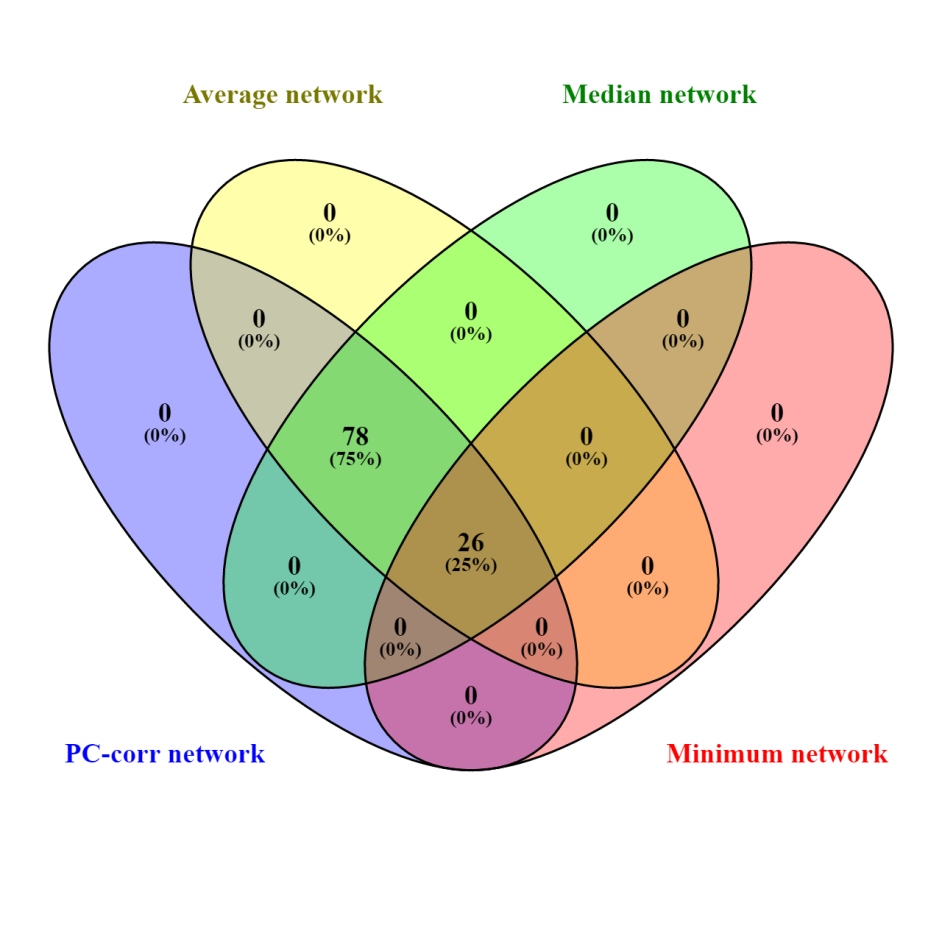
**

b

**Edges**

Figure S15. Comparison of the PC-corr network and the three networks obtained from LOOCV under cut-off 0.8. (a) Venn diagrams that show the node differences between the PC-corr network and the average, minimum and median networks obtained from LOOCV under cut-off 0.8. (b) Venn diagrams that show the edge differences between the PC-corr network and the average, minimum and median networks obtained from LOOCV under cut-off 0.8.

**Carcinoma cell-wide promoteromic dataset**

**
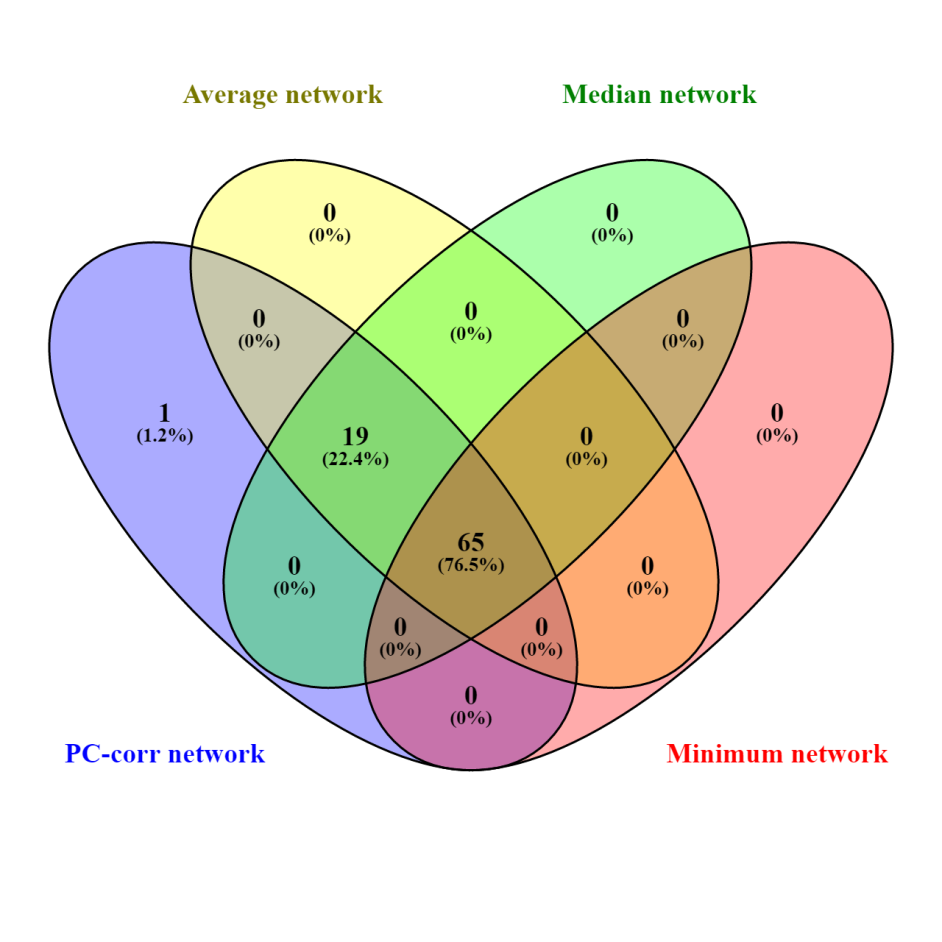
**

a

**Nodes**

**
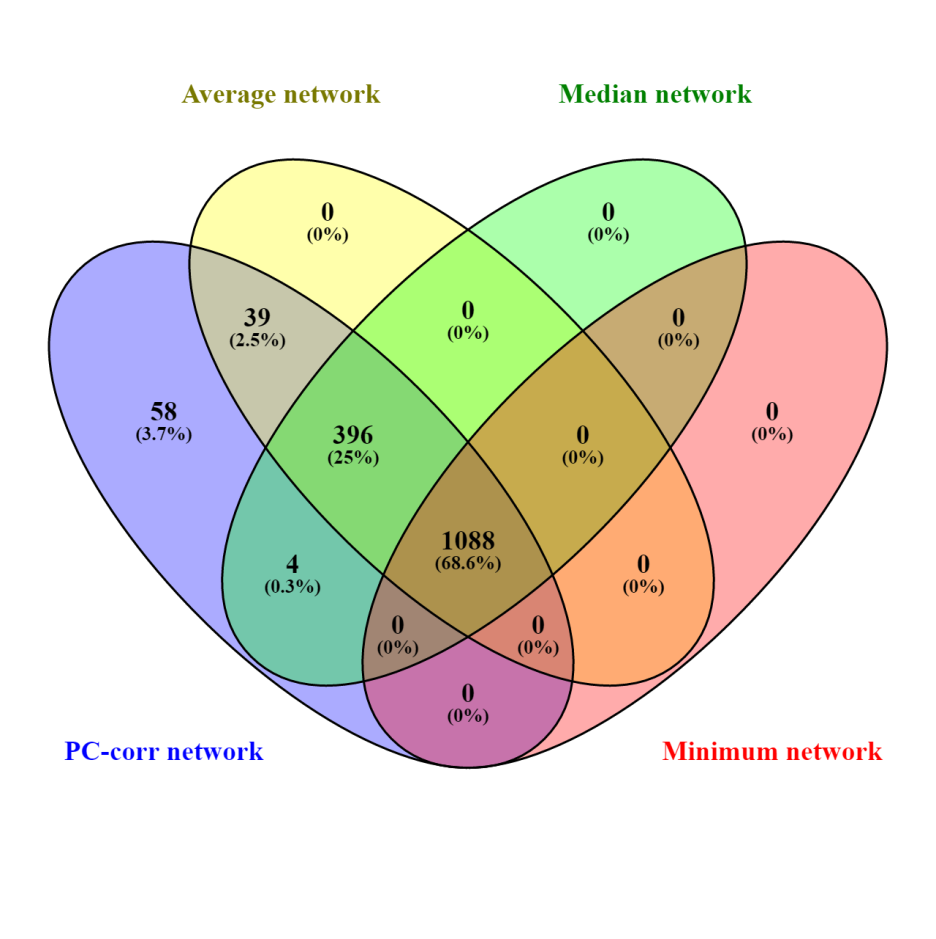
**

b

**Edges**

Figure S16. Comparison of the PC-corr network and the three networks obtained from LOOCV under cut-off 0.8. (a) Venn diagrams that show the node differences between the PC-corr network and the average, minimum and median networks obtained from LOOCV under cut-off 0.8. (b) Venn diagrams that show the edge differences between the PC-corr network and the average, minimum and median networks obtained from LOOCV under cut-off 0.8.

**Cancer stem cell mechanomics**

**
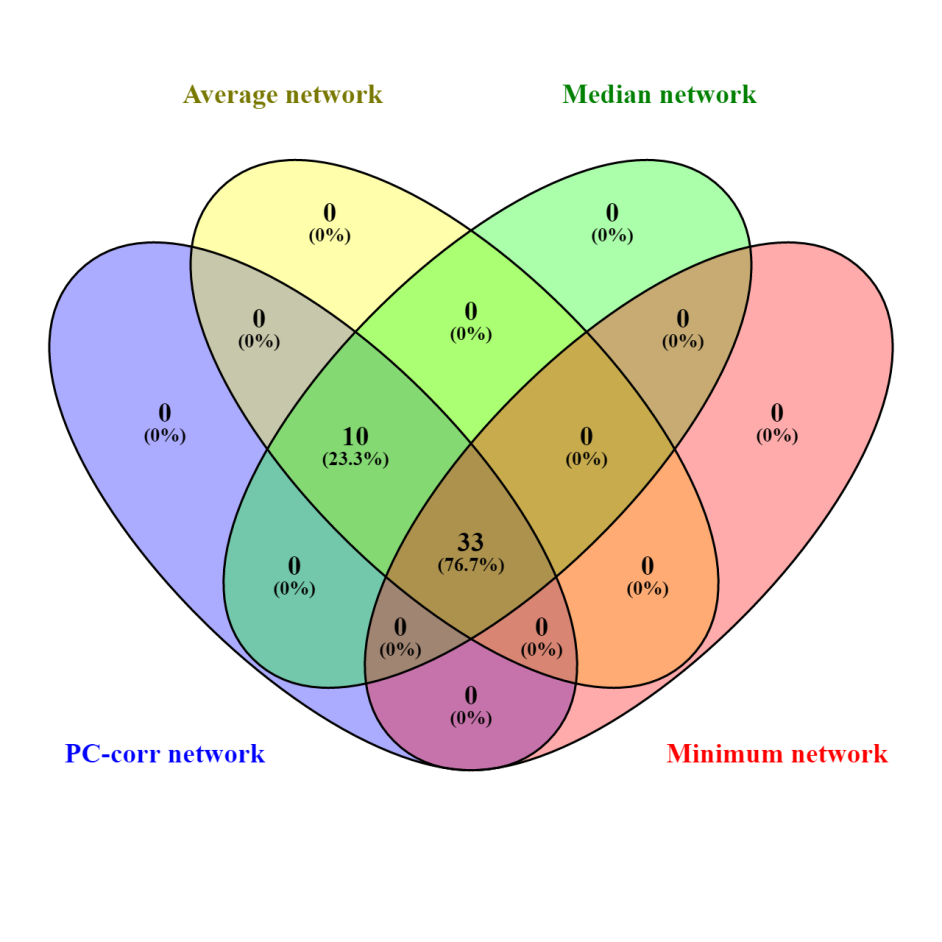
**

a

**Nodes**

**
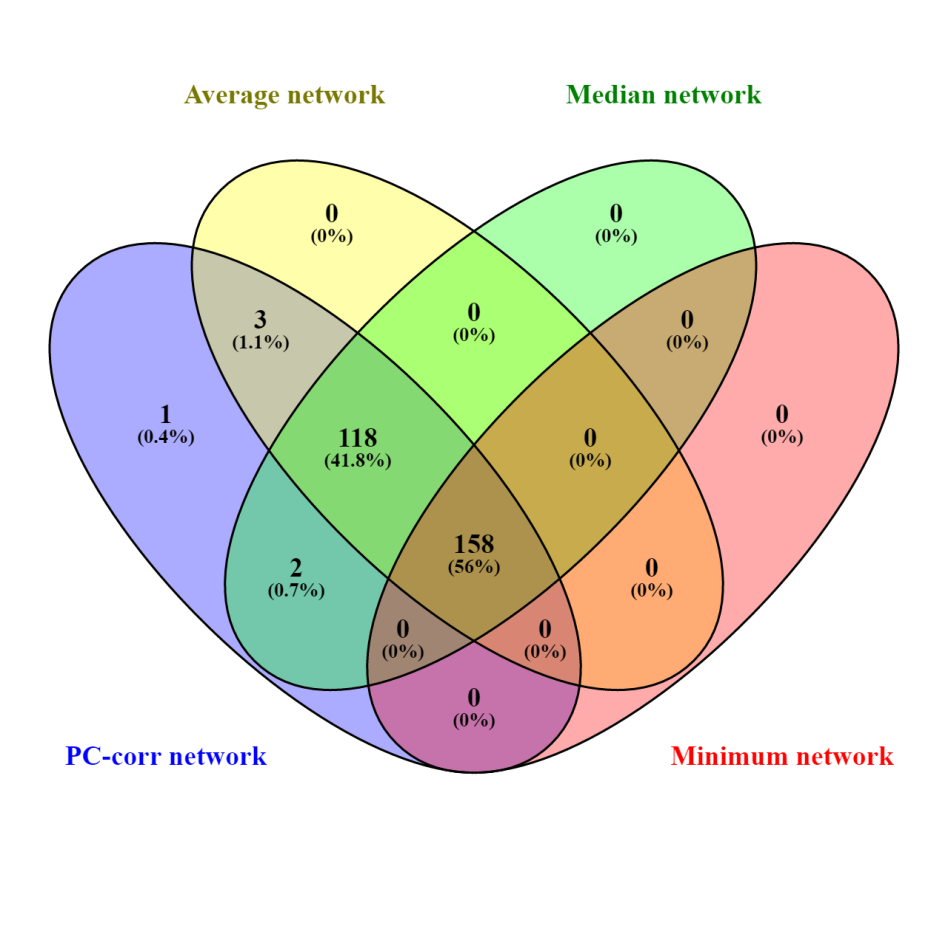
**

b

**Edges**

Figure S17. Comparison of the PC-corr network and the three networks obtained from LOOCV under cut-off 0.65. (a) Venn diagrams that show the node differences between the PC-corr network and the average, minimum and median networks obtained from LOOCV under cut-off 0.65. (b) Venn diagrams that show the edge differences between the PC-corr network and the average, minimum and median networks obtained from LOOCV under cut-off 0.65.
